# Polyoxometalates as Potential Next‐Generation Metallodrugs in the Combat Against Cancer

**DOI:** 10.1002/anie.201803868

**Published:** 2018-10-12

**Authors:** Aleksandar Bijelic, Manuel Aureliano, Annette Rompel

**Affiliations:** ^1^ Universität Wien Fakultät für Chemie Institut für Biophysikalische Chemie Althanstraße 14 1090 Wien Austria; ^2^ Universidade do Algarve Faculdade de Ciências e Tecnologia (FCT), CCMar 8005-139 Faro Portugal

**Keywords:** antitumor agents, biological activity, cancer, nanoparticles, polyoxometalates

## Abstract

Polyoxometalates (POMs) are an emerging class of inorganic metal oxides, which over the last decades demonstrated promising biological activities by the virtue of their great diversity in structures and properties. They possess high potential for the inhibition of various tumor types; however, their unspecific interactions with biomolecules and toxicity impede their clinical usage. The current focus of the field of biologically active POMs lies on organically functionalized and POM‐based nanocomposite structures as these hybrids show enhanced anticancer activity and significantly reduced toxicity towards normal cells in comparison to unmodified POMs. Although the antitumor activity of POMs is well documented, their mechanisms of action are still not well understood. In this Review, an overview is given of the cytotoxic effects of POMs with a special focus on POM‐based hybrid and nanocomposite structures. Furthermore, we aim to provide proposed mode of actions and to identify molecular targets. POMs are expected to develop into the next generation of anticancer drugs that selectively target cancer cells while sparing healthy cells.

## 1 Introduction

Cancer is a malignant disease in which abnormal cells divide in an uncontrolled way, leading to the formation of a solid mass referred to as tumor or to blood cancers.^1^ According to the WHO (http://www.who.int/cancer/en/), cancer is one of the leading causes of mortality worldwide and was responsible for 8.8 million deaths in 2015. The most prominent cytotoxic drug class is cisplatin (CDDP), which is still one of the most applied chemotherapeutic agents in clinics. Besides CDDP, a series of other drugs have shown their anticancer potential by temporarily alleviating symptoms, prolonging the lifespan of patients and in rare cases even curing the cancer.^2^ However, all of them suffer from major disadvantages such as severe side effects owing to lack of selectivity, low efficiency against some cancer types, and low bioavailability. Therefore, there is still the quest for alternative drugs selectively incapacitating cancer cells without severely damaging normal cells.

**Figure 1:**
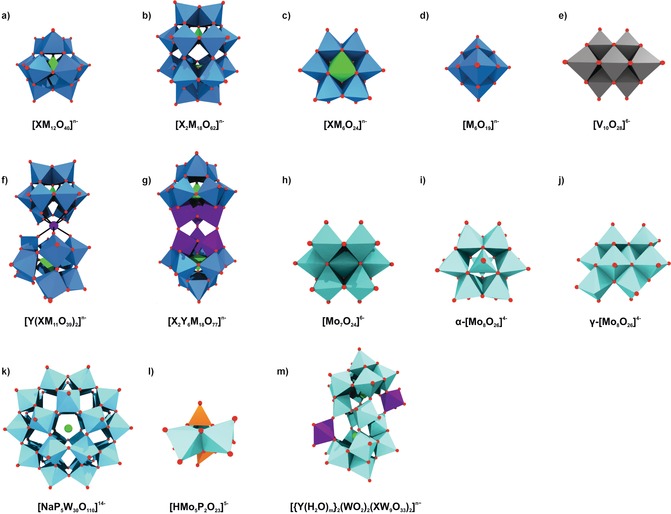
Overview of common POM archetypes. a) Keggin, b) Wells–Dawson, c) Anderson, d) Lindqvist, e) decavanadate, f) sandwich Keggin, g) double Keggin, h) heptamolybdate, i) α‐ and j) γ‐octamolybdate, k) Preyssler, l) Strandberg, and m) Krebs‐type structure. Blue polyhedra are {MO_6_} (M=any addenda atom), light green polyhedra {XO_*n*_} (X=heteroatom), light green spheres sodium, light blue polyhedra {WO_6_}, light cyan polyhedra {MoO_6_}, gray polyhedra {VO_6_}, purple polyhedra and spheres {YO_*n*_} and Y (Y=second heteroatom), orange polyhedra {PO_4_}, red spheres oxygen.

In this context, polyoxometalates (POMs), which are described as clusters of transition metal (W, Mo, V, Nb) and oxygen atoms, have in the last decades been found to be promising anticancer drug candidates. POMs exhibit an overwhelming diversity in size and structure (Figure 1) with outstanding properties and functions.^3^ They have been studied vigorously and are used in a wide range of applications such as catalysis,^4^ nanoscience,^5^ macromolecular crystallography,^6^, ^7^, ^8^ and medicine.^9^, ^10^ The anticancer activity of POMs was first mentioned in 1965, when Mukherjee described the in vivo application of a mixture named PTMC, a combination of H_3_[PW_12_O_40_], H_3_[PMo_12_O_40_] and caffeine, on patients suffering from gastrointestinal cancer.^11^ Despite leading to the complete disappearance of tumors in four patients, PTMC was not subjected to further clinical studies. Years later, in 1974, Jasmin et al. described the inhibitory effect of (NH_4_)_17_Na[NaSb_9_W_21_O_86_] against sarcoma virus‐induced tumors.^12^ Since then, considerable attention was paid to the development of biologically active POMs.^9^, ^13^ In this regard, especially Yamase and co‐workers performed some important pioneering work by synthesizing [NH_3_Pr^i^]_6_[Mo_7_O_24_] (PM‐8) that has been evaluated for its in vitro and in vivo anticancer activities.^14^, ^15^ PM‐8 was highly efficient in vivo by suppressing the tumor growth in different mice models being partially more active than approved drugs such as 5‐fluorouracil (5‐FU) and nimustine.^16^ In 1991, Fujita et al. tested 50 POMs for their anticancer activity, among them different variants of PM‐8, and found only four promising compounds, namely PM‐8, the reduced form of PM‐8 (PM‐17), [NH_3_Pr^i^]_6_[Mo_7_O_26_] (PM‐26), and Na_5_[IMo_6_O_24_] (PM‐32).^17^ Yamase noticed that the reduced form of PM‐8, namely PM‐17, which was later identified as [H_2_Mo^V^_12_O_28_(OH)_12_(Mo^VI^O_3_)_4_]^6−^,^18^ was highly toxic in comparison to PM‐8. Therefore, he proposed a mechanism for the anticancer activity of PM‐8 which involves its reduction to PM‐17 and its re‐oxidation in which course the tumor cells are reduced and thus killed.^19^ The proposed mechanism seemed reasonable as PM‐8 can be biologically reduced by FMN, an electron carrier responsible for the electron transport from NADH to coenzyme Q.^19^, ^20^ This process is coupled with the generation of ATP and therefore the proposed redox‐cycle mechanism is based on the inhibition of ATP formation.

Despite the success story of PM‐8 and others, purely inorganic POMs mostly suffer from high and long‐term toxicity, impeding their clinical application.^21^ Therefore, the research focus is switching from inorganic POMs to POM‐based organic‐inorganic hybrids as the functionalization and/or encapsulation of POMs with organic moieties not only reduced the toxicity of the POM in most cases but also increased its anticancer activity. In this Review, we report on the development and recent advances in the synthesis of POMs with proven antiproliferative activity. Our focus lies especially on organically modified POMs and POM‐based nanocomposites and their potential application as chemotherapeutic agents. This Review provides a comprehensive overview of proposed molecular targets and mode of actions. Moreover, we present an outlook discussing why POMs, despite their disadvantages, are still potential next‐generation metallodrugs in the combat against cancer.

## 2 Anticancer Activity of Polyoxometalates

### 2.1 Anticancer Activity of Purely Inorganic Polyoxometalates

After the auspicious results from PM‐8 and other polyoxomolybdates (POMos; Supporting Information, Table S1), interest in this type of compounds peaked, leading to a vast number of biologically active POMs (Supporting Information, Tables S1–S3). In 2005, Liu et al. tested the antiproliferative activity of in total 21 POMs against KB cells (HeLa derived human oral carcinoma). The results revealed that the structure has a major impact on the antitumoral activity as the polyoxovanadate (POV) K_7_[NiV_13_O_38_] (IC_50_=0.29–0.36 mg L^−1^) and the heptamolybdate PM‐8 (Figure 1 h, IC_50_=0.40 mg L^−1^) were the most active POMs followed by a series of Anderson POMs (Figure 1 c, IC_50_=0.45–0.53 mg L^−1^), whereas POMs exhibiting the Keggin (Figure 1 a, IC_50_=1.9–43.5 mg L^−1^) and Wells–Dawson structure (Figure 1 b, IC_50_=34.5–52.3 mg L^−1^) were by far the least active clusters.^22^ It is similar with the type of addenda atom as POVs were slightly more active than POMos followed by the least active polyoxotungstates (POTs). Mixed‐type POMs showed increased activity when a W atom was substituted by a V or Mo atom. This is in accordance to the proposed redox‐based mechanism of Yamase as the oxidation power follows the sequence POVs>POMos>POTs.^23^

The promising antitumor activity of POVs is not surprising, as the most prominent representative, decavanadate [V_10_O_28_]^6−^ (Figure 1 e), is known to be highly bioactive, including excellent antitumor activity,^24^, ^25^, ^26^ owing to its high affinity towards important enzymes such as kinases,^27^ actin,^28^ and P‐type ATPases.^29^, ^30^ A series of POVs exhibited strong antitumor activity against different cancer cell lines (Supporting Information, Table S3) such as K_12_[V_18_O_42_(H_2_O)], of which activity against the breast adenocarcinoma cell line MCF‐7 was superior to that of the approved drug 5‐FU (inhibitory rate at 250 μm ca. 70 % vs. ca. 20 %).^31^ The Co^II^ containing decavanadate Na_4_Co(H_2_O)_6_[V_10_O_28_] was the only POV that was tested in vivo inhibiting the tumor growth in murine liver cancer Hep‐A‐22 bearing mice by 47.1 % at a dose of 6 mg kg^−1^.^24^ The activity resembled that of 5‐FU (80.5 % at 20 mg kg^−1^), however, the POV was clearly better tolerated by mice, as the body weight was less‐affected by the polyanion. Besides its in vivo activity, the POM inhibited in vitro the proliferation of human hepatocellular (SSMC‐7721) and ovarian (SK‐OV‐3) carcinoma cell lines (IC_50_=0.3 and 0.2 μg mL^−1^). Comparison with Na_6_[V_10_O_28_] (IC_50_=9.9 and 18.9 μg mL^−1^) revealed that the introduction of Co^II^ as counterion has considerably increased the activity of decavanadate.

The anticancer activity of other POMs also benefited from the incorporation of Co^II^ or other transition metals (TMs). For example, the addition of Co^II^ to the trilacunary Keggin POM [SbW_9_O_33_]^9−^ led to the formation of the sandwich structure (Figure 1 f) Na_9_[{Na(H_2_O)_2_}_3_{Co(H_2_O)}_3_(α‐B‐SbW_9_O_33_)_2_], of which antiproliferative activity against human ovarian (SK‐OV‐3, IC_50_=33.3 μg mL^−1^), hepatocellular (SSMC‐7721, IC_50_=20.7 μg mL^−1^) and liver cancer cells (Hep‐G2, IC_50_=9.3 μg mL^−1^) was significantly higher than that of the parent POM (IC_50_=203.6, 219.6 and 214.0 μg mL^−1^).^32^ The increase in anticancer activity was explained by the Co^II^ induced changes in polarity, acidity, and redox properties of the POM unit facilitating the cell penetration and target interactions of synergistic Co^II^‐POM systems.

A series of Krebs‐type tungstobismuthates (Figure 1 m), (H_2_im)_2_[(W_0.5_Ni_0.5_(H_2_O))_2_(Ni(H_2_O)_3_)_2_(Na_4_(H_2_O)_14_)(BiW_9_O_33_)_2_], (H_2_im)_2_[(WO(OH))_2_(Zn(H_2_O)_3_)_2_(Na_4_(H_2_O)_13_)(BiW_9_O_33_)_2_], (Him)_2_[(W(OH)_2_)_2_(Mn(H_2_O)_3_)_2_(Na_3_(H_2_O)_14_)(BiW_9_O_33_)_2_] and (H_2_im)_2_[(W(OH)_2_)_2_(Co(H_2_O)_3_)_2_(Na_4_(H_2_O)_14_)(BiW_9_O_33_)_2_] (im=imidazole), showed promising anti‐liver cancer (Hep‐G2) activity (Supporting Information, Table S2).^33^ The Ni^II^ containing POM was the most active compound (IC_50_=25.6 μm), followed by the Mn^II^ and Zn^II^ containing clusters (IC_50_=30.1 and 32.3 μm), whereas the Co^II^‐POM (IC_50_=37.3 μm) was the least active one. All tested POMs performed significantly better than CDDP (IC_50_=66.1 μm) against Hep‐G2 cells. However, activity tests using human hepatocyte (QSG) cells revealed also a significant activity against normal cells (IC_50_=32.4 μm (Ni^II^), 43.2 μm (Mn^II^), 49.7 μm (Zn^II^), and 38.5 μm (Co^II^)). Based on this, the Zn^II^ containing Krebs‐POM was the most selective and thus clinically most suitable compound.^33^ The Mn^II[34]^ and Co^II[35]^ containing Krebs structures were also tested on other cancer cell lines performing partially better than clinically approved drugs and were shown to induce apoptosis (Supporting Information, Table S2).

Another POM inducing evidently apoptosis is the Cu^II^ containing double Keggin POT (Figure 1 g) K_7_Na_3_[Cu_4_(H_2_O)_2_(PW_9_O_34_)_2_], which showed inhibitory effects against both human (MG‐63) and rat (UMR‐106) bone osteosarcoma (IC_50_=22 and 81 μm).^36^ The POM increased the intracellular level of reactive oxygen species (ROS) while reducing that of the ROS‐scavenger glutathione (GSH) via GSH‐POM interactions leading to the dissipation of the mitochondrial membrane potential and finally to apoptosis. Moreover, the cytotoxic effect of the POT on MG‐63 was higher than that of CDDP (IC_50_=43 μm).

All of the reported inorganic POMs exhibiting antiproliferative activity are summarized in the Supporting Information, Tables S1–S3.

### 2.2 Anticancer Activity of Inorganic–Organic Hybrid Polyoxometalates

The functionalization of POMs with organic groups is the main focus of the field of bioactive POMs, as purely inorganic POMs generally exhibit toxic side effects and limited cell penetration owing to their surface characteristics. The introduction of organic moieties into the POM framework can change its surface, charge, polarity, and redox properties, leading to a completely new compound with reduced toxicity and increased cell penetration ability. Organically modified POMs are in general more stable in aqueous solutions and, depending on the attached functionality, their interaction with biological targets are enhanced and more specific.

#### 2.2.1 Organometallo‐Substituted Polyoxometalates

Many organometallic molecules possess promising anticancer activities and are thus a good choice for the hybridization with POMs to develop hybrids with enhanced biological activity.^37^ A series of studies have been published describing mainly the in vitro antiproliferative activity of organometallo‐substituted POTs containing organotin RSn (R=C_4_H_7_O_2_, C_5_H_9_O_2_, and NC_3_H_4_) or metal–cyclopentadienyl CpM^n+^ groups (Cp=η^5^‐C_5_H_5_, M=Ti^IV^, Zr^IV^, V^IV^, Fe^II^) against human cervical (HeLa) and liver (SSMC‐7721) cancer cells (Supporting Information, Table S4).^38^, ^39^, ^40^, ^41^, ^42^, ^43^, ^44^ The tested structures were Keggin‐, sandwich Keggin‐, and Wells–Dawson‐type structures with the general formulas [(RSn)_*m*_XW_12−*m*_O_40−*m*_]^*n*−^, [(RSn)_3_(XW_9_O_34_)_2_]^*n*−^, [(CpM)_*m*_XW_12−*m*_O_40−*m*_]^*n*−^, and [(CpM)_*m*_X_2_W_18−*m*_O_62−*m*_]^*n*−^ (X=heteroatom, *m*=1–3). Compounds with attached cyanoethyltin groups (NC_3_H_4_Sn) were more active than those containing estertin groups (C_4_H_7_O_2_Sn and C_5_H_9_O_2_Sn), sandwich‐Keggin were superior to Keggin structures, and with increasing RSn content the antitumor activity was enhanced. For very closely related compounds the antitumor activity correlates with their redox potential, that is, the higher the redox potential, the higher the cytotoxicity. In general, CpM^*n*+^ containing POMs exhibited higher antitumor activities than the RSn structures (Supporting Information, Table S4).^45^, ^46^ The influence of the metal in the CpM^*n*+^ group on the antitumor activity is cancer cell specific as given cells are more or less susceptible to a certain metal. K_6_H[(η^5^‐C_5_H_5_Ti)CoW_11_O_39_] was applied orally to human liver (SSMC‐7721), leukemia (HL‐60), and colon cancer (HLC) bearing mice (for 10 d) and significantly decreased the growth of all tumors exhibiting inhibitory rates of 41.9 % (dose=15 mg kg^−1^), 50.0 % (100 mg kg^−1^) and 48.9 % (100 mg kg^−1^), respectively.^47^ The POT performed better than the clinically approved drug cyclophosphamide (CP) against SMMC‐7721 (inhibitory rate=37.2 % at 36.4 mg kg^−1^) but was less active than 5‐FU against the other cell lines (56–57.4 % at 15–30 mg kg^−1^), however, it was by far the least toxic compound.^47^

The organotin substituted Keggin K_3_H[{(*n*‐Bu)Sn(OH)}_3_GeW_9_O_34_] inhibited the tumor growth in the H22 (murine liver cancer) mice model by 62.5 % after 14 d (dose=300 mg kg^−1^).^48^ The activity was significantly lower than that of CP (inhibitory rate=95.5 %), however, the drug also impaired the growth of mice, which is indicative of toxic side effects, whereas the POM did not. The hybrid showed also promising in vitro activity against a series of other cancer cells (Supporting Information, Table S4).

The antitumor activity of other organometallo substituted POMs is listed in the Supporting Information, Table S4.

#### 2.2.2 Polyoxometalate–Drug Hybrids

##### 2.2.2.1 Polyoxometalate‐5‐fluorouracil Hybrids

The combination of POMs with drug molecules is a promising strategy in cancer treatment, as POM–drug hybrids are multi‐functional systems capable of interacting with multiple targets. In this way, the hybrids are less prone to drug resistance and could exhibit improved activity and selectivity. The combination of the anticancer drug 5‐FU with the Keggin POTs [PW_12_O_40_]^3−^, [SiW_12_O_40_]^4−^, and [BW_12_O_40_]^5−^ led to POT‐5‐FU hybrids that were more active against the tumor cell lines HeLa, Hep‐G2, and SMMC‐7721 than 5‐FU alone, indicating synergistic effects (Supporting Information, Table S5).^49^, ^50^, ^51^ Furthermore, the hybrids were less toxic against normal human embryonic kidney cells (HEK‐293) than the free drug. The [PW_12_O_40_]‐5‐FU and [SiW_12_O_40_]‐5‐FU systems were later extended by the introduction of rare earth metals (Dy, Eu, Er, Gd, La, Nd, Pr, Sm, Y), which in most cases did not only lead to an increase in activity (against HeLa and Hep‐G2) but also to enhanced selectivity (Supporting Information, Table S5).^51^, ^52^, ^53^, ^54^, ^55^ However, POM‐rare earth metal complexes (without 5‐FU) like K_11_[L(PW_11_O_39_)_2_] (L=Dy, Er, Gd, La, Y) were remarkably less active than the corresponding 5‐FU containing hybrids, indicating no significant synergy between the POM and rare earth metals.

##### 2.2.2.2 Polyoxometalate–Bisphosphonate Hybrids

**Figure 2:**
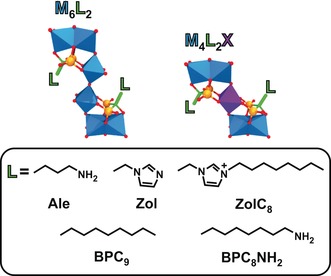
General structure of the POM‐BP compounds M_6_L_2_ and M_4_L_2_X. L=bisphosphonate side chains, which are depicted at the bottom. The phosphonate group is depicted in ball and stick mode (orange spheres, phosphorous; red spheres, oxygen). Blue polyhedra are {MO_6_} (M=V, Mo, W), purple polyhedra {XO_6_} (X=Fe^III^, Mn^II/III^), green sticks carbon, red spheres oxygen.

The Dolbecq group investigated the antitumor activity of a series of POM‐bisphosphonate complexes.^56^, ^57^, ^58^, ^59^ Bisphosphonates (BPs) are drugs used to treat osteoporosis and similar diseases but do also exhibit antitumor activity.^60^ BPs have the general formula H_2_O_3_PC(OH)(R)PO_3_H_2_ with R determining their drug efficacy, as, for example, the primary nitrogen containing alendronate (Ale, R=(CH_2_)_3_NH_2_) is 100–1000 times less active than zoledronate (Zol, R=(H_2_(C_3_H_3_N_2_)), which has a heterocyclic amine at position R.^61^ POM‐BP complexes are not classical POM‐based structures, and the most studied compounds exhibited the general formulas M_6_L_2_ and M_4_L_2_X (M={MoO_6_}, {WO_6_}, {VO_6_}; L=BP and X=Mn^II/III^, Fe^III^) (Figure 2). The hybrids were active on human non‐small cell lung cancer (NCI‐H460), glioblastoma (SF‐268), and breast cancer (MCF‐7) cells, whereby V‐based complexes were the most active (Supporting Information, Table S6). Molybdates and tungstates had only noticeable or good antitumor activity in combination with the most bioactive BP Zol. Thus, the antitumor activity of the POM‐BP systems correlated with the presence of either V or Zol. The most potent complexes, not containing V, were Mo_4_Zol_2_Mn^II/III^, indicating that the introduction of Mn^II/III^ as heteroatom has a significant effect on the antitumor activity of these complexes. Mo_4_Zol_2_Mn^III^ was the only POM‐BP complex that was studied in vivo.^59^ Applied to the mouse xenograft model bearing human Ewing sarcoma (SK‐ES‐1), Mo_4_Zol_2_Mn^III^ (5 μg/mouse for 28 d) decreased the tumor volume by about 85 % in comparison to the control (buffer). Furthermore, the complex did not reduce the body weight, indicating the absence of harmful effects on mice. Regarding the mechanism, amino group containing BPs are known to inhibit the prenylation of important proteins like small GTPases, which are involved in tumor growth, and therefore the POM‐BP complexes could follow a similar mechanism (see Section 3.2).^62^

##### 2.2.2.3 Polyoxometalate–Quinolone Hybrids

**Figure 3:**
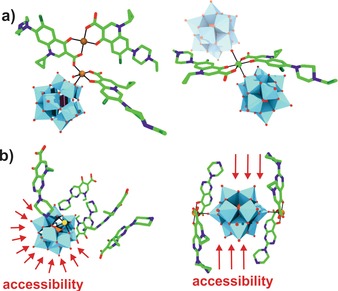
Structures of POM–quinolone hybrids. a) [Cu_2_(Enro)_3_H_2_O][SiW_12_O_40_] (left) and H_2_[Ni(Enro)_2_][SiW_12_O_40_] (right). The adjacent POM found in the crystal structure of H_2_[Ni(Enro)_2_][SiW_12_O_40_] is indicated by a transparent molecule; however, in solution this site is most probably occupied by a solvent molecule. b) [HPPA]_5_[PW_11_CdO_39_] (left) and [Cu(PPA)_2_]_2_[PW_12_O_40_] (right). Red arrows indicate the accessible interaction sites. Light blue polyhedra are {WO_6_}, orange polyhedra {PO_4_}, green sticks carbon, dark blue sticks nitrogen, dark green sticks fluorine, brown spheres copper, green sphere nickel, yellow sphere cadmium, red sticks and spheres oxygen.

Another example for POM‐drug complexes with antitumoral properties are POM–quinolone antibiotic structures.^63^, ^64^, ^65^, ^66^, ^67^, ^68^ Quinolone antibiotics such as pipemidic acid (PPA) inhibit the growth of gram‐negative bacteria by preventing DNA from unwinding and duplicating. Owing to their structure, which contains abundant O and N donors, quinolones are excellent multidentate ligands and thus ideal to modify POMs. The Keggin structures [PW_12_O_40_]^3−^, [PMo_12_O_40_]^3−^, and [SiW_12_O_40_]^4−^ and the octamolybdates β‐ and δ‐[Mo_8_O_26_]^4−^ were decorated with different quinolone antibiotics such as PPA, enrofloxacin (enro), norfloxacin (norf), and enoxacin (eno). In most cases a TM (Cu^II^, Zn^II^, Ni^II^, Co^II^) was additionally incorporated into the structures, leading to different POM‐TM‐quinolone complexes, where the POM acts as a mono‐ or bidentate inorganic ligand and the quinolone as an organic ligand for the TM. In POM–TM–quinolone systems, the POM is covalently linked to the TMs and quinolones, whereas in TM‐lacking compounds the quinolones are clustered around the POM via noncovalent interactions. The complexes exhibited mixed results against a series of cancer cell lines as only a couple of them showed good antitumor activity, whereas the vast majority showed no to moderate activity (Supporting Information, Table S7). Depending on the used constituents, complexes with different coordination modes and POM–quinolone interactions were obtained. In general, hybrids possessing a TM center with a five‐coordinate geometry were less active than those with a six‐coordinated TM (Figure 3 a), and systems with a bidentate POM ligand (one POM binding two TMs, Figure 3 b, right) were more active than those having a monodentate POM ligand. Thus, it was proposed that certain structural constellations might favor the delocalization of the whole electrons, which increases hybrid–tumor interactions, whereas other constellations lead to rather unsymmetrically polarized POMs with quenched activity.^64^ Furthermore, it was suggested that structures like [Cu(PPA)_2_]_2_[PW_12_O_40_], where the POM unit is surrounded by quinolones, are less active as the interaction of the POM with tumor cells is sterically hindered in comparison to complexes containing freely accessible interaction sites (Figure 3 b).

#### 2.2.3 Anderson Polyoxometalate–Biomolecule Hybrids

**Figure 4:**
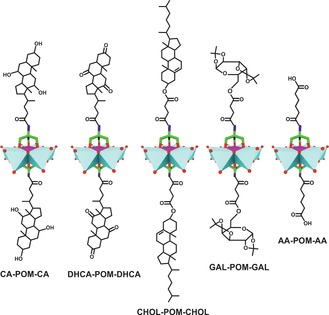
Biomolecule‐functionalized Anderson structures. Cyan polyhedra are {MoO_6_}, magenta polyhedra {MnO_6_}, green sticks carbon, dark blue sticks nitrogen, red spheres oxygen.

Regarding the organic modification of POMs, the Anderson archetype is one of the best studied systems as it can be easily tris‐functionalized (tris=tris(hydroxymethyl)aminomethane) leading to an amino group(s) bearing structure, which can be further modified by simple amidation.^69^, ^70^, ^71^, ^72^ Thus, the Anderson structure represents an ideal basis for the synthesis of versatile POM‐ligand complexes. Yang et al. synthesized a series of POM‐biomolecule conjugates by grafting different bioactive receptor ligands to the surface of the Anderson POM to improve the selectivity and thus the antitumor activity of the POM.^73^ The tris‐modified Anderson‐type molybdate {MnMo_6_O_18_[(OCH_2_)_3_CNH_2_]_2_}^3−^ (tris‐POM‐tris) was used as starting molecule to attach cholic acid (CA), dehydrocholic acid (DHCA), *O*‐succinyl‐cholesterol (CHOL), 6‐*O*‐(3‐carboxypropanoyl)‐1,2:3,4‐di‐*O*‐isopropylidene‐β‐d‐galactopyranose (GAL) and adipic acid (AA) to the POM (Figure 4).

CA and DHCA are bile acids that target the farnesoid X receptor (FXR), a nuclear receptor, which induces cell death in some breast cancer cell lines upon activation.^74^ CHOL is a precursor for the synthesis of bile acids and building block of the cell membrane, which might be helpful for cancer cell targeting. GAL is the diacetal form of galactose, which is recognized by some lectins of which expression is strongly associated with tumorigenesis and thus could enhance cancer cell selectivity.^75^ AA is an organic dicarboxylic acid, which was used for comparison reasons. The cytotoxicity of these hybrids was tested against the breast cancer cell lines MCF‐7 and MDA‐MB‐231 and the noncancerous breast cell cline MCF‐10A (Supporting Information, Table S8). As expected, the most effective complexes were CA‐POM‐CA (IC_50_=55.9, 37.9 and 278.2 μm) and DHCA‐POM‐DHCA (IC_50_=112.7, 149.0 and >400 μm) exerting the highest selectivity and antitumor activity. The remaining complexes were only weakly or moderately active (IC_50_=204–400 μm). The significant synergy between the POMo and CA/DHCA derives from the capability of all constituents to induce apoptosis, whereby the bile acids impart selectivity to the complex by targeting FXR.

#### 2.2.4 Polyoxometalate–Amino Acid Hybrids

Amino acids are promising organic units to functionalize POMs owing to their structural and chemical variety and biocompatibility. A string of amino acid functionalized POMs was synthesized and some of them showed promising anticancer activity (Supporting Information, Table S9).

The γ‐isomer of octamolybdate, γ‐[Mo_8_O_26_]^4−^ (Figure 1 j), was modified with different amino acids, yielding the hybrids Na_4_[Mo_8_O_26_(ala)_2_], Na_4_[Mo_8_O_26_(glygly)_2_], [Hmorph]_4_[Mo_8_O_24_(OH)_2_(met)_2_], and [Hmorph]_4_[Mo_8_O_24_(OH)_2_(ala)_2_] (ala=alanine, glygly=glycylglycine, morph=morpholine, met=methionine) that selectively inhibited the cell growth of human liver and breast cancer cells (Hep‐G2 and MCF‐7) while showing no significant effect on other cancer cell lines (Supporting Information, Table S9).^76^ The antiproliferative activity of these hybrids was not cell‐cycle related and thus not induced by apoptosis. Cartuyvels et al. later proposed that the antitumor activity of such hybrids and octamolybdate in general could be related to the hydrolysis of ATP as they observed ATP hydrolysis in the presence of Na_4_[Mo_8_O_26_(pro)_2_] (pro=proline) at acidic pH (<5.8).^77^ However, the role of ATP hydrolysis during cancer progression is elusive as ATP exhibits biphasic actions, that is, it has both tumor promoting and inhibitory effects.^78^

The glycine‐decorated POMo K_2_Na[AsMo_6_O_21_(gly)_3_] (gly=glycine) showed weak to moderate inhibitory effects against human lung carcinoma cells (A‐549, IC_50_=330.2 μm), which were still superior to that of 5‐FU (ca. 40 % inhibition at 1 mm).^79^ However, the arsenomolybdate was later found to be highly active on the human leukemia cell lines HL‐60 and U937 (IC_50_=8.6 and 14.5 μm; Supporting Information, Table S9) being more active than all‐*trans* retinoic acid (ATRA, IC_50_=20.8 μm vs. HL‐60 and 14.9 μm vs. U937), a clinical anticancer drug, but less active than the antileukemic agent As_2_O_3_ (IC_50_=6.4 μm vs. HL‐60 and 8.8 μm vs. U937).^80^ However, As_2_O_3_ is also highly cytotoxic towards normal cells such as human umbilical vein endothelial cells (HUVECs) for which it exhibits an alarming IC_50_ value of 5.6 μm, 81 while K_2_Na[AsMo_6_O_21_(gly)_3_] has no significant activity on HUVECs (IC_50_=889.2 μm). The selective antileukemic activity of the hybrid might arise from As^III^ but its low toxicity towards normal cells in comparison to As_2_O_3_ renders it a promising alternative for the treatment of leukemia.

Other amino acid functionalized POMs with antitumor activity are listed in the Supporting Information, Table S9.

#### 2.2.5 Organically Functionalized Strandberg‐type Polyoxometalates

The Strandberg‐type POM, [Mo_5_P_2_O_23_]^6−^ (Figure 1 l), is a small POM with a very high charge density, making it ideally suited to interact with cationic units such as organometallic compounds. The Strandberg POM–benzimidazole (biz) hybrid [Hbiz]_5_[HMo_5_P_2_O_23_] showed cytotoxic effects against human bone narrow neuroblastoma (SHY5Y) cells (IC_50_=43 μm).^82^ Biz is a well‐known pharmacophore of which derivatives are active against several cancer types.^83^ [Hbiz]_5_[HMo_5_P_2_O_23_] is highly selective towards neuroblastoma cells as it did not show any significant activity against other cancer or normal cell lines (Supporting Information, Table S10). The anti‐SHY5Y activity of the hybrid was inferior to that of pure biz (IC_50_=28.7 μm) but biz was also highly toxic on normal cells (IC_50_=21.5 μm vs. EVC‐304), making the hybrid considerably more valuable from a pharmacological point of view.

Another study reporting Strandberg‐type hybrids describes the antitumor activity of [Cu(pia)_2_(H_2_O)_2_]_2_H_2_[P_2_Mo_5_O_23_], [Cu(pia)_2_ (H_2_O)]H_2_[Cu(pia)_2_(P_2_Mo_5_O_23_)] and [Cd(pia)_2_(H_2_O)_2_]_2_H_2_[P_2_Mo_5_O_23_] (pia=pyridine‐2‐carboxamide; Supporting Information, Figure S1).^84^ All of the compounds exhibited promising activity against human hepatoma Hep‐G2 and SMMC‐7721 cells and colorectal carcinoma HCT‐116 cells (IC_50_=2.7–35.5 μm), with the exception of [Cu(pia)_2_(H_2_O)_2_]_2_H_2_[P_2_Mo_5_O_23_], which was not active on HCT‐116 (Supporting Information, Table S10). [Cu(pia)_2_(H_2_O)]H_2_[Cu(pia)_2_(P_2_Mo_5_O_23_)] was the most tumor selective compound in this study, as its activity against normal human liver cells (HL‐7702) was 3–17 times lower than that against the tested tumor cells. Despite having similar structures, [Cu(pia)_2_(H_2_O)]H_2_[Cu(pia)_2_(P_2_Mo_5_O_23_)] and [Cd(pia)_2_(H_2_O)_2_]_2_H_2_[P_2_Mo_5_O_23_] showed clearly distinct cytotoxic behavior emphasizing the importance of the TM type. Furthermore, the coordination mode of the TM is also highly important as the activity of the two Cu^II^ containing hybrids differed remarkably, which is in accordance with the results of the POM‐TM‐quinolone hybrids (Section 2.2.2.1).

#### 2.2.6 Organoimido‐ and Benzoyldiazenido‐Functionalized Hexamolybdates

**Figure 5:**
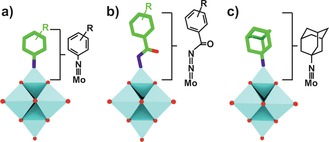
General structure of the aromatic organoimido (a), benzoyldiazenido (b), and aliphatic organoimido‐substituted hexamolybdates (c). Cyan polyhedra {MoO_6_}, green sticks carbon, dark blue sticks nitrogen, red sticks and spheres oxygen.

Organoimido and benzoyldiazenido substituted hexamolybdates (Figure 1 d) exerted inhibitory effects against human leucocythemia K‐562 cells (Figure 5 a,b; Supporting Information, Table S11). The former group has the general structure (TBA)_2_[Mo_6_O_18_(≡NAr)] (TBA=tetra‐*n*‐butyl ammonium, Ar=aryl group), whereas that of the latter is (TBA)_3_[Mo_6_O_18_(=N=NCOAr)]. The aromatic ring in both systems can be variously substituted providing the ability to attach a wide range of organic compounds to the POM core.^85^, ^86^, ^87^ The organoimido derivates were slightly more active than the benzoyldiazenido bearing POMs as the former group is more redox‐active. Another hybrid group, namely (TBA)_2_[Mo_6_O_19−*n*_(≡NC_10_H_15_)_*n*_] (NC_10_H_15_=amantadine, *n*=1–3, Figure 5 c) containing one or more aliphatic organoimido groups, showed moderate anti‐breast cancer activity (inhibition rate=48.3 % at 100 mg mL^−1^ vs. MCF‐7 cells) but promising effects against malignant glioma U‐251 cells (IC_50_=31.1 μm).^88^, ^89^

**Figure 6:**
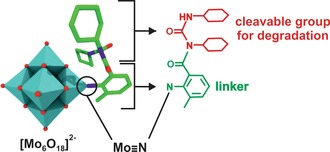
Structure of POM‐AMB‐acy. For clarity, the structural formula of AMB (green) and acy (red) is additionally depicted. Cyan polyhedra {MoO_6_}, green sticks carbon, dark blue sticks nitrogen, red sticks and spheres oxygen.

However, the most interesting organoimido substituted POM, regarding its anticancer activity, is POM‐AMB‐acy (AMB=2‐amino‐3‐methylbenzoxyl group, acy=N‐acylureido group) (Figure 6).^89^ The hybrid inhibited the growth of U‐251 cells (IC_50_=24.8 μm) and, even more importantly, was able to cross the blood–brain‐barrier in vivo. POM‐AMB‐acy represents a degradable compound, where the acy group was introduced for degradation reasons and AMB as linker. The hybrid complex stayed intact in medium for 40 minutes before decomposing into the bioactive species upon acy degradation. According to mass spectrometry the bioactive species is most likely monomeric MoO_4_^2−^. POM‐AMB‐acy induced apoptosis in U‐251 cells and was significantly more active than the clinically used antiglioma agent temozolomide (TMZ, IC_50_ ca. 500 μm).^90^

#### 2.2.7 Other Inorganic–Organic Hybrid Polyoxometalates

Some organically functionalized polyoxoniobates (PONbs) were reported to possess promising antitumor activity (Supporting Information, Table S12). For example, the two Cu^II^ containing undecaniobates, [{Cu(H_2_O)L}_2_{CuNb_11_O_35_H_4_}]^5−^ (L=1,10‐phenanthroline, 2,2′‐bipyridine; Supporting Information, Figure S2) exhibited remarkable antiproliferative activity against K‐562 (leukemia) cells (IC_50_=0.1–0.4 μm).^91^ Since pure PONbs are redox‐stable, a redox‐based mechanism for their activity could be excluded. Another example of PONb‐based hybrids are the vanadium cluster substituted structures {Ni(en)_3_}_5_H{V^V^Nb_8_V^IV^_8_O_44_} and (H_2_en)Na_2_[{Zn(en)_2_(Hen)}{Zn(en)_2_(H_2_O)}_2_{PNb_8_V^IV^_8_O_44_}] (en=1,2‐diaminoethane; Supporting Information, Figure S3), which showed partially high antiproliferative activity against the human gastric cancer cell lines SGC‐791, SC‐1680, and MG‐63 (IC_50_=0.7–20.5 μm).^92^

Hybridization of the photosensitizing agent pyridinium chlorin with [SiMo_12_O_40_]^4−^ led to a significant improvement of its phototoxicity against A‐549 cancer cells.^93^ The increased photoactivity of (chlorin)_4_[SiMo_12_O_40_] (IC_50_=6.6 μm) in comparison to sole chlorin (IC_50_ >20 μm) is based on the POM‐mediated increase in the intracellular photogeneration of ROS (^1^O_2_) and improved cellular uptake of the complex.

Zhang et al. synthesized the homochiral POM anion {CoSb_6_O_4_(H_2_O)_3_[Co(hmta)SbW_8_O_31_]_3_}^15−^ (hmta=hexamethylenetetramine) via chiral symmetry breaking and asymmetric autocatalysis obtaining both racemic and enantiopure (Δ‐ or Λ‐enantiomer) POM complexes.^94^ The racemic and enantiopure hybrids exhibited similar anticancer activity against eight cancer cell lines, which were significantly higher than that of the achiral precursor [NaSb_9_W_21_O_86_]^18−^ (Supporting Information, Table S12). The compounds showed high selectivity towards the ovarian cancer cell lines A2780 and OVCAR‐3 (IC_50_=0.8–4.5 μm) and were also active on the CDDP‐resistant cell line A2780cisR (IC_50_=4.4–4.5 μm).

The anticancer activity of other inorganic–organic hybrids are summarized in the Supporting Information, Table S12.

### 2.3 Anticancer Activity of Polyoxometalate‐based Nanocomposites

The encapsulation of drugs is an important area in biomedicine, as the resulting nanocomposites bring many advantages such as enhanced drug stability, delivery, and activity and the extension of the bioactivity by protecting the drug from premature degradation, which is also associated with minimal side effects.^95^ Using this system, the drug is in most cases released slowly and gradually leading to a prolonged time window within which the therapeutic level of the drug is sustained. In this way, the pharmacokinetic behavior of a series of POMs was partially dramatically improved.

#### 2.3.1 Polyoxometalate–Chitosan Nanocomposites

POM–chitosan hybrids are one of the most studied POM‐based nanocomposite systems.^26^ Chitosan (CT) is a linear polysaccharide obtained by the N‐deacetylation of chitin and one of the most widely applied natural polymers in drug‐delivery studies. CT‐based systems are ideal in terms of extended drug release, as CT is degraded in the human body by several enzymes, leading to a controlled release.^96^ Biocompatibility studies showed that the nanocomposites {Co_4_(H_2_O)_2_(PW_9_O_34_)_2_}–CMC and {Eu(SiW_11_O_39_)_2_}–CMC (CMC=carboxymethyl chitosan) did not show any activity against HeLa cells even at high concentrations (2 mg mL^−1^) and long incubation times (up to 48 h).^97^, ^98^ Furthermore, {Eu(SiW_11_O_39_)_2_}–CMC undergoes fast cellular uptake (within 1 h), confirming the toxicity reducing and cellular transport enhancing effect of CT. Comparison between the Keggin‐type {CoTiW_11_O_40_}–CMC and {CoTiW_11_O_40_}–TMC (TMC=trimethyl chitosan) hybrids revealed that the carrier properties of the positively charged TMC are superior to that of its negatively charged counterpart CMC.^99^ The TMC matrix had a circa 6 times higher POM content, owing to its positive charge and the associated direct electrostatic interactions, and it was more readily uptaken by cells. The reason for this was attributed to the smaller particle size of the POM‐TMC hybrid (61 vs. 131 nm), the different morphology and positive charge facilitating cell penetration. The release profile of {CoTiW_11_O_40_}–TMC showed a slow but steady POM release (ca. 15 %) over 24 h. Moreover, the TMC nanocomposite exhibited cytotoxic effects on HeLa cells (inhibitory effect=50 % at 12.5 μg mL^−1^, 5 h), whereas the parent POM was rather inactive (ca. 10 % at 50 μg mL^−1^, 24 h).

{Gd(W_5_O_18_)_2_}–CT_siRNA_ nanospheres possess high potential as radiosensitizers for synergistic radiotherapy and gene therapy as they remarkably reduced the radioresistance of human hepatocellular carcinoma cells (BEL‐7402) in vitro and in vivo.^100^ Radiotherapy is a clinically applied cancer treatment method, which utilizes high‐intensity ionizing radiation to inhibit tumor growth by the generation of cytotoxic ROS. However, cells in general possess high amounts of reducing agents such as GSH to combat the production of ROS and, even more important, tumor cells are highly hypoxic minimizing the available amount of activatable oxygen and thus limiting the therapeutic effect of radiotherapy. Hypoxia is lethal to all cells; therefore, tumors develop a set of responses to outstrip their blood supply to adapt to the stressful hypoxic environment.^101^ The master mediator of this response is the hypoxia‐inducible factor 1α (HIF‐1α).^102^ Therefore, the authors synthesized a CT‐based system consisting of the ROS‐level increasing [Gd(W_5_O_18_)_2_]^9−^ and HIF‐1α siRNA, which interferes with the expression of HIF‐1α, to reduce the radioresistance of cancer cells. {Gd(W_5_O_18_)_2_}–CT_siRNA_ in combination with X‐ray radiation induced significant cell late apoptosis/necrosis response in both HeLa and BEL‐7402 cells (33.6 and 28.8 %), whereas the same hybrid in absence of either X‐rays (10.9 and 14.4 %) or siRNA ({Gd(W_5_O_18_)_2_}–CT, 14.4 and 15.9 %) showed clearly lower apoptotic effects. The in vivo experiment using BALB/c mice inoculated with BEL‐7402 cells (injection every second day for 25 d) confirmed the in vitro results. Treatment with {Gd(W_5_O_18_)_2_}–CT_siRNA_ under X‐ray radiation did almost completely suppress the tumor growth as the relative tumor volume (the tumor volume measured/initial tumor volume=*V*/*V*_0_) was about 1 (*V*/*V*_0_ of control ca. 25). The hybrid had no reducing effects on the body weight, indicating no significant systemic toxicity. The suggested mechanism is described in Section 3.2.5.

**Figure 7:**
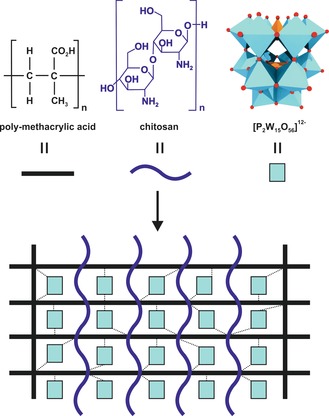
The pH‐responsive POM‐based hydrogels. Dashed lines represent POM–polymer interactions.

Electrostatically driven pH‐responsive supramolecular hydrogels consisting of the trilacunary Wells–Dawson [P_2_W_15_O_56_]^12−^, chitosan hydrochloride (CTCl),^103^ or CMC^104^ and poly(methacrylic acid) (PMMA) were antiproliferatively active on breast and cervical cancer cells (MCF‐7 and HeLa) with minimal effects on normal Vero cells (Figure 7; Supporting Information, Table S13). Hydrogels are highly hydrated polymeric networks, including both covalent and non‐covalent interactions, and are ideal to mimic native tissues owing to their soft consistency, porosity, and stability. At the maximum applied concentration of 35 mg mL^−1^ the hydrogels were slightly less active against both MCF‐7 and HeLa (inhibitory effect ca. 72–77 and 68–70 %) than the free POM (ca. 80 and 85 %) and the chemotherapy agent doxorubicin (DOX, ca. 77 % against both cells) but clearly more biocompatible. The incorporation of the pH responsive agent MMA led to a pH‐controlled swelling of the gel and thus to a sustained POM release. In vivo experiments using rabbits revealed a maximum tolerable dose of 4000 mg kg^−1^ and no significant histopathological effects on important organs.

Other POM‐CT hybrids with anticancer activity are summarized in the Supporting Information, Table S13.

#### 2.3.2 Polyoxometalate–Starch Nanocomposites

Starch is a polysaccharide consisting of glucose units linked by α‐1,4‐gylcosidic bonds that is extensively used in pharmaceutics as a drug carrier owing to its biocompatibility, biodegradation, and stability properties.^105^ The starch‐encapsulated POM (SEP) {CoTiW_11_O_40_}–SEP showed increased anti‐cervical (HeLa) and anti‐leukemia (HL‐60) activity compared to the free Keggin POM (Supporting Information, Table S14).^106^ The increase in the antitumoral effect was attributed to the enhanced cell penetration ability of the starch complex, which was quantified revealing a HeLa cell penetration efficiency of 83.1 % being more than three times higher than that of the parent POM (25.5 %). However, {CoTiW_11_O_40_}–SEP was stable for only 4 h (pH 7.4) before undergoing decomposition. Similar penetration efficiencies were obtained for the dimeric titanotungstosilicate [Si_2_Ti_6_W_18_O_77_]^14−^ and its starch encapsulated nanoparticles (24.6 vs. 87.2 %), confirming the starch‐mediated improvement in cell penetration.^107^ The in vitro release profile revealed an initial burst effect within the first 2–3 h, where weakly bound POMs at the nanoparticle surface are quickly released (ca. 40 % of POM), followed by a sustained release. Such an initial burst effect, which can lead to local and toxic overdoses of POM, was not observed for CT‐based nanoparticles. In vitro antitumor tests showed that the IC_50_ values of {Si_2_Ti_6_W_18_O_77_}–SEP against HeLa and HL‐60 cells (3.2 and 8.1 μg mL^−1^) were significantly lower than that of the free POM (46.4 and 60.5 μg mL^−1^). The in vivo activity of the SEP in H22 bearing rats (inhibitory rate=44.2 % at 96 mg kg^−1^) was also superior to that of the parent POM (40 % at 200 mg kg^−1^) but inferior to that of the clinical drug CP (44 % at 36.4 mg kg^−1^). The LD_50_ value of [Si_2_Ti_6_W_18_O_77_]^14−^ was increased from 1803 to at least 2855 mg kg^−1^ upon starch encapsulation.

The anticancer activity of further POM‐SEP systems is summarized in the Supporting Information, Table S14.

#### 2.3.3 Polyoxometalate–Liposome Nanoparticles

Liposomes are small spherical vesicles possessing at least one lipid bilayer. Owing to their ability to minimize the systemic toxicity of a hosted drug and protecting it from early degradation, liposomes are ideal to modify the pharmacokinetic behavior of POMs.^105^ The liposome‐encapsulated POM (LEP) {Si_2_Ti_6_W_18_O_77_}–LEP was synthesized in two different sizes, namely 60 and 150 mm, with the smaller nanoparticles having a slightly higher activity against HeLa and HL‐60 cells (IC_50_=3.2 and 4.6 μg mL^−1^ vs. 4.4 and 5.2 μg mL^−1^), which might be attributed to the facilitated cell penetration of the smaller particles.^108^ The antitumor activity of this LEP was comparable to that of its starch encapsulated counterpart {Si_2_Ti_6_W_18_O_77_}–SEP. Studies with other POMs like the Keggin structures [SiM_11_O_39_Co(H_2_O)]^6−^ (M=Mo, W) and [PV_2_Mo_10_O_40_]^5−^, which were encapsulated by both biomaterials, revealed that the corresponding LEPs (IC_50_=4.5–13.4 μm) and SEPs (IC_50_=5.2–14.5 μm) exhibit similar antitumor activities (Supporting Information, Tables S14, S15).^109^, ^110^, ^111^ Regarding the drug release, {CoTiW_11_O_40_}–LEP, which has anticancer activity in hepatocellular cancer and leukemia cells, SSMC‐7721 and HL‐60 (IC_50_=3.5 and 3.6 μm), showed a slow and sustained release (ca. 20 % POM release).^112^ In comparison, the same POM encapsulated by TMC showed a similar release profile but with a faster initial release, whereas the starch hybrid of this POM decomposes after several hours, indicating that the liposome matrix is the most suitable carrier for [CoTiW_11_O_40_]^8−^.

The penetration efficiency of LEPs is similar to that of the two biopolymers discussed before as about 81 % of {SiTiW_11_O_40_}–LEP was found inside HeLa cells and HUVECs, whereas the penetration efficacy of the POM alone was only about 25 %.^113^ The anticancer activity of {CoTiW_11_O_40_}–LEP against KB and HeLa cells (IC_50_=2.2 and 2.3 μg mL^−1^) was higher than that of the free POM (IC_50_=34.5 and 47.3 μg mL^−1^) and 5‐FU (Supporting Information, Table S15). However, its in vivo inhibitory effect on the tumor growth in HL‐60 bearing rats (42 % at 26.4 mg kg^−1^) was inferior to that of 5‐FU (58.3 % at 25 mg kg^−1^) but still remarkably higher than that of the pristine POM (13 % at 200 mg kg^−1^). The LD_50_ value of [SiTiW_11_O_40_]^6−^ was increased from 349 to 2003 mg kg^−1^ upon liposome encapsulation.

**Figure 8:**
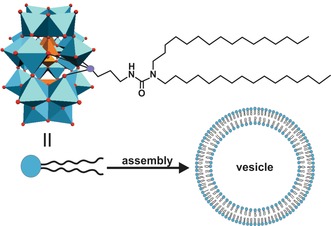
Illustration of the spontaneous assembly of the POM–lipid hybrid into a vesicle. Light blue polyhedra are {WO_6_}, orange polyhedra {PO_4_}, purple sphere silica, red spheres oxygen.

The hybrid POM [(C_16_H_33_)_2_NCONH(CH_2_)_3_SiNaP_5_W_29_O_110_]^14−^ (P_5_W_29_‐lipid), which is composed of the mono‐lacunary Preyssler anion [NaP_5_W_29_O_107_]^14−^ with an attached long‐chain organoalkoxysilane lipid, exhibited promising antiproliferative activity against human colon adenocarcinoma cells (HT‐29).^114^ The lipid covalently binds to the lacuna of the POM via its organosilicate functionality giving the hybrid an amphiphilic character. The hybrid spontaneously self‐assembles into a liposome with the POM forming the lipid bilayer (Figure 8). Although the parent [NaP_5_W_30_O_110_]^14−^ (Figure 1 k) has already a strong anti‐HT‐29 activity (IC_50_=3.6 μm), it was even improved by the lipid‐hybridization (IC_50_=2.1 μm). Owing to its special structure, the hybrid formed stable complexes with biotinylated sBLM, a natural membrane mimetic. Therefore, an uptake mechanism was proposed, where the P_5_W_29_‐lipid binds to the membrane and then intercalates into it forming a transient hybrid‐membrane complex, which finally releases the POM within the cytoplasmic space.

#### 2.3.4 Polyoxometalate–Silica Nanocomposites

**Figure 9:**
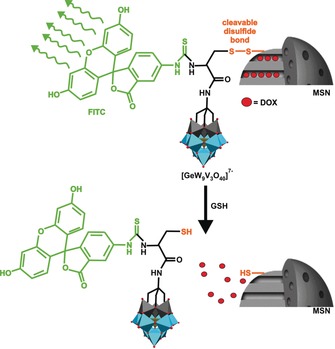
Representation of the POM‐MSN‐dye‐DOX system. At the top of the figure the disulfide bond (orange) is intact and the attached POM‐dye (dye=green) hybrid blocks the release of DOX molecules (big red spheres) from the MSN pores. After disulfide cleavage by GSH, the POM‐dye hybrid and DOX are released. Light blue polyhedra {WO_6_}, gray polyhedra {VO_6_}, tan polyhedra {GeO_4_}, small red spheres oxygen.

Silica‐based nanoparticles are promising candidates for the drug delivery in cancer therapy because their particle size can be finely tuned to ensure appropriate accommodation of guest molecules, which is especially true for mesoporous silica nanoparticles (MSNs).^115^ Karimian et al. reported on a complex delivery system consisting of a thiolated MSN as drug carrier that covalently binds the N‐Boc‐cysteine‐functionalized Keggin‐type POM [GeV_3_W_9_((CH_2_O)_3_N‐Boc‐Cys)O_37_]^4−^ (Boc=*tert*‐butyloxycarbonyl, Cys=cysteine) via a disulfide bond.^116^ The addition of the fluorescent dye fluorescein isothiocyanate (FITC) to the organic functionality of the POM yields the final POM‐MSN‐dye complex, where the pores of the MSN are capped by the POM and loaded with the anticancer drug DOX (Figure 9). The redox‐responsive disulfide bond was chosen for selective POM‐release as cancer cells contain elevated levels of GSH, which is able to cleave disulfide bonds, whereas FITC was attached for cellular tracking reasons. [GeV_3_W_9_O_40_]^7−^ was chosen as POM unit as it exhibited the highest antiproliferative activity against human glioblastoma cells (U‐87, inhibitory effect=44 % at 50 μg mL^−1^) among a series of tested POMs, [XM_3_W_9_O_40_]^*n*−^ (X=Si, Ge; M=V, W, Mo) (Supporting Information, Table S2). The POM‐MSN‐dye complex loaded with different concentrations of DOX exhibited selective anticancer activity against U‐87 cells (inhibitory effect=70 % at 2 mg mL^−1^ DOX) as it was significantly less active on normal cells (ca. 40 % at 2 mg mL^−1^ DOX). Compared to the multidrug complex, the antitumor activities of the sole POM and DOX (ca. 50 % at 2 mg mL^−1^) were clearly inferior. As expected the activity of the hybrid increased in a DOX concentration‐dependent manner with the DOX release being GSH concentration‐dependent.

The anticancer activity of other silica‐based POMs is summarized in the Supporting Information, Table S16.

#### 2.3.5 Other Polyoxometalate‐based Nanocomposites

{Mo_7_O_24_}–gelatin nanoparticles showed promising anticancer activity in vitro and in vivo.^117^ Gelatin is a natural polymer that is highly biocompatible and owing to its zwitterionic character it forms a stable hydrophobic complex with heptamolybdate (Figure 1 i). At high concentrations, the {Mo_7_O_24_}–gelatin nanoparticles exhibited cytotoxicity to human gastric cancer cells (BGC‐823) in vitro, which was significantly higher than that of the plain POM (inhibitory rate=75 vs. 20 % at 0.5 mg mL^−1^), however, at lower concentrations (<0.25 mg mL^−1^) the hybrid was inactive. The in vivo experiment using ICR mice inoculated with murine liver cancer cells (H22) confirmed the tumor‐inhibiting potential of the hybrid as the relative tumor volume (*V*/*V*_0_) was 14.5 upon treatment with 20 mg kg^−1^ of {Mo_7_O_24_}–gelatin for 9 d, whereas *V*/*V*_0_ of the control and the free POM (100 mg kg^−1^) was about 35 and about 27, respectively. All tumor‐bearing mice died within 31 days in the control group, whereas the hybrid group (100 mg kg^−1^) exhibited a survival rate of 60 % after 40 d (ca. 20 % in the heptamolybdate group, 100 mg kg^−1^).

The Pt^IV^ substituted Keggin‐type POM [PW_11_O_40_(SiC_3_H_6_NH_2_)_2_Pt^IV^(NH_3_)_2_Cl_2_]^3−^ was synthesized as a pharmacological prodrug that has to undergo Pt^IV^ to Pt^II^ reduction to be activated.^118^ However, the cell penetration properties of this organoplatinum substituted POM are limited leading to a low in vitro anticancer activity (inhibitory effect ca. 35 % at 20 μm vs. HT‐29). Therefore, the prodrug was encapsulated with 1,2‐distearoyl‐*sn*‐glycero‐3‐phosphoethanolamine‐N‐[methoxy(polyethylene glycol)‐2000] (DSPE‐PEG_2000_) yielding the nanocomposite Pt^IV^‐PW_11_‐DSPE‐PEG_2000_. As expected, the hybrid was readily internalized into HT‐29 cells, which was also reflected in its anti‐HT‐29 activity (inhibitory effect=85 % at 20 μm) being clearly superior to that of CDDP (ca. 45 %). The nanocomposite showed low toxicity towards normal HUVECs (inhibitory effect ca. 30 % at 50 μm), whereas CDDP in comparison was highly toxic (ca. 60 %). Upon reduction (Pt^IV^→Pt^II^) by GSH, Pt^II^‐PW_11_ interacts with DNA, leading to apoptosis. The hybrid exhibited also promising in vivo activity as it almost completely reduced the tumor size of HT‐29‐bearing BALB/c mice (dose=0.8–2 mg kg^−1^) performing better than CDDP without exhibiting body‐weight‐reducing effects. As Pt complexes are known for their nephrotoxicity, the Pt level in the rat kidney was determined, revealing that the treatment with Pt^IV^‐PW_11_‐DSPE‐PEG_2000_ led to considerably lower Pt levels than in the case of CDDP, rendering the hybrid an effective alternative for cancer therapy.

The anticancer activity of other POM‐based nanocomposites is summarized in the Supporting Information, Table S16.

## 3 Proposed Mechanisms of the Anticancer Activity of Polyoxometalates

### 3.1 Cell Penetration of Polyoxometalates

**Figure 10:**
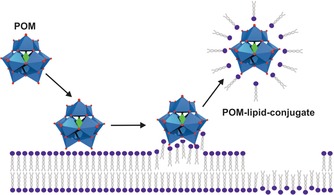
Representation of the destructive interaction between POMs and membranes. The POM adsorbs to the membrane surface and forms a stable POM–lipid conjugate that desorbs from the membrane.

The antiproliferative activity of an anticancer drug is directly associated with its degree of cellular uptake. It is well accepted that POMs are able to penetrate cancer cells, as many studies have experimentally confirmed their location within the cytoplasmic space.^46^, ^99^, ^109^, ^110^, ^111^, ^119^ However, no convincing data has been reported revealing the exact mechanism. Owing to their large size and negative charge, POMs are supposed to be rather unable to penetrate the largely negatively charged cell membranes of mammalian cells. POMs such as the Keggin‐type were characterized as super chaotropic agents with a surprisingly high tendency to adsorb on neutral and hydrophilic surfaces, whereby POMs with lower charge densities were more chaotropic.^120^ Owing to this feature, POMs exhibited destructive activity towards model cell membranes by adsorbing to the vesicle surface, followed by the formation of a stable POM–lipid conjugate, which finally desorbs from the membrane (Figure 10).^121^, ^122^ These desorption processes led to leaky membrane structures. Studies with anionic lipids revealed that depending on the charge density, the POM–lipid interactions can switch from electrostatic to hydrophobic nature, owing to the charge neutralization by cationic counterions.^123^

Nevertheless, it is still generally assumed that POMs are mainly taken up by cells by some form of endocytosis. Different POTs were traced within murine macrophage J774 cells revealing POM containing vacuoles within the cells, wherefore, it was suggested that POMs might bind to scavenger receptors and enter the cell by scavenger receptor‐mediated endocytosis.^124^ Similarly, huge POMo nanoparticles such as (NH_4_)_42_[{(Mo^VI^)Mo_5_^VI^O_21_(H_2_O)_6_}_12_{Mo_2_^V^O_4_(CH_3_COO)_30_}] were found to be located within endosomes that were evenly distributed in the cytoplasm of Hep‐G2 cells, supporting internalization by an endocytotic pathway.^119^

Monitoring of the FITC labeled {Eu(SiW_11_O_39_)_2_}–CMC within HeLa cells showed that the nanoparticles were preferably located in the perinuclear region in close proximity to the nuclei.^98^ Clathrin‐mediated endocytosis was proposed as POM‐free chitosan nanoparticles are internalized by this pathway; however, this was later excluded as the cellular uptake of POM‐CT was not inhibited by the clathrin inhibitor chlorpromazine indicating a clathrin‐independent pathway.^99^ Thus, macropinocytosis and caveolae‐mediated endocytosis were suggested as internalization pathways for, at least, POM‐CT hybrids as chitosan‐DNA‐poly(γ‐glutamic acid) complexes are uptaken by these pathways.^125^

### 3.2 Proposed Modes of Action and Biological Targets of Antitumoral Polyoxometalates

**Figure 11:**
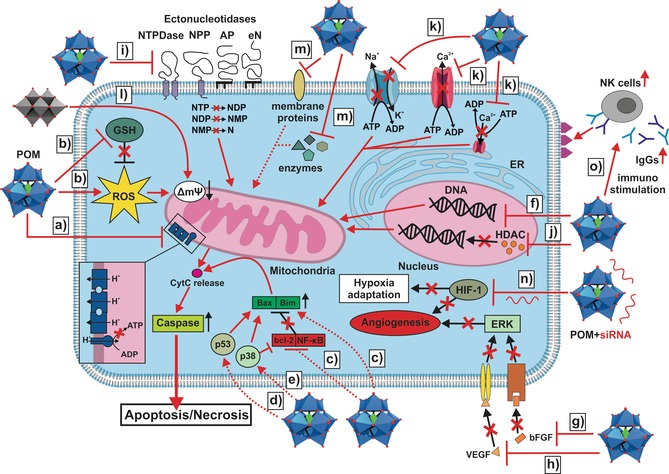
Illustration of most of the proposed modes of action of antitumoral POMs. a) POM induced inhibition of ATP synthesis by interfering with the electron transfer chain (represented as dark blue entities, the inset shows a zoomed view of the chain). b) POM induced increase in ROS‐level (for example by oxidizing cell components) and depletion of the GSH pool (by GSH oxidation). c) POM induced enhancement of the expression of pro‐apoptotic components (Bax and Bim) and the reduction of the expression of anti‐apoptotic components (bcl‐2 and NF‐κB). d),e) Activation of the p53 and/or p38 pathway by POMs. Please note: the circles reading p38 and p53 do not represent the respective protein but the pathways. f) Induction of apoptosis by direct DNA damage. g),h) POM‐mediated inhibition of angiogenesis via interaction with bFGF and VEGF leading to the disruption of the VEGF/bFGF‐receptor interactions (receptors are indicated as yellow and brownish channels). Without VEGF/bFGF‐receptor binding, the ERK pathway cannot be activated leading to the breakdown of angiogenesis. i) Inhibition of ectonucleotidases by POMs leads to a distortion in the concentrations of nucleotides (NTP, NDP, and NMP) and nucleosides (Ns), which negatively affects the functioning of cancer cells. j) Inhibition of HDAC by POMs leads to the accumulation of acetylated histones, causing fatal changes in the expression of genes. k) Inhibition of P‐type ATPases has fatal effects on the cellular ion homeostasis. l) Decavanadate‐induced mitochondria membrane depolarization. m) Inhibition of other proteins that affect cell viability (for more details, see text of Section 2.3.1 and 3.2.5, and references therein). n) POM hybrids loaded with siRNA (siRNA is depicted as red RNA structure) downregulate HIF‐1, leading to the impairment of angiogenesis and the adaptation of cancer cells to the hypoxic environment. o) Immunostimulating activity of POMs by promoting the expression of antibodies and immune‐related components (for example, NK cells). The figure depicts the activation of NK cells via antibody binding enhancing the recognition of tumor cells (antigens are depicted as purple triangles) by NK cells. Dotted lines indicate that the reason of activation/deactivation (for example, enhanced/decreased expression) of certain components is not known. The release of cytochrome c (purple circle) triggers the apoptotic machinery of the cell, which ultimately activate the final executers of apoptosis (caspases).

Figure 11 shows most of the putative mechanisms of antitumoral POMs in a comprehensive Scheme. The first suggested mechanism for the antitumor activity of POMs was that by Yamase, which was already mentioned in the introduction.^19^ Briefly, repeated reduction/oxidation cycles between the POM and cell components, most likely members of the electron transport chain, are supposed to interfere with ATP generation, which finally leads to apoptosis (Figure 11 a). The theory is widely accepted, as a series of studies revealed a putative correlation between the cytotoxicity and the redox potential of bioactive POMs.^38^, ^39^, ^40^, ^41^, ^42^, ^43^, ^44^ This correlation is a weak one and only applies to very closely related structures, as other factors like size, structure, and composition play also a significant role. In general, there is no unambiguous correlation between the anticancer activity of POMs and parameters such as POM size, total net charge, charge density, oxidation power, and archetype.

#### 3.2.1 Activation of Cell Death Pathways

Apoptosis, necrosis, and autophagy are types of cell death, which are generally highly sophisticated processes including complex signaling cascades that are tightly regulated. According to Section 2, a number of POMs induced apoptosis by the mitochondrial (intrinsic) pathway as no study describes, for example, the involvement of a death receptor, which is a hallmark for extrinsic apoptosis. The reason for this is that most POMs mainly induced “internal damage” by oxidative stress, which is signaled to mitochondria (Figure 11 b). Some POMs like the Wells–Dawson POM are able to induce apoptosis by affecting the expression of cell death regulators, for example, by increasing the amount of the pro‐apoptotic proteins Bax and Bim (Figure 11 c).^126^, ^127^ Other POMs, such as Krebs‐type, do also reduce the expression of the anti‐apoptotic protein bcl‐2 and the transcriptional factor NF‐κB (Figure 11 c).^33^, ^34^ Active NF‐κB is responsible for the expression of genes that protect the cell from undergoing apoptosis, however, many tumor types have a constantly active NF‐κB and therefore its inhibition represents a promising approach in cancer therapy. Furthermore, Krebs‐type tungstobismuthates are able to increase the expression of p53 activating apoptosis partially by this pathway (Figure 11 d).^33^ The tumor suppressor p53 is a transcription factor that induces anti‐carcinogenesis events such as cell growth arrest and inhibition of angiogenesis, making it the guardian of the genome and one of the most promising targets as about 50 % of human cancers are thought to be related to p53 mutations.^128^ Some POMs like the Co^II^ containing Krebs‐type (H_2_im)_2_[(W(OH)_2_)_2_(Co(H_2_O)_3_)_2_(Na_4_(H_2_O)_14_)(BiW_9_O_33_)_2_] are able to enhance the activation of caspase‐3, the final executioner of apoptosis.^35^, ^127^ Na_7_[Cr^III^Cu^II^W_11_O_39_] was observed to induce the upregulation of both cytochrome c and activated p38 in human ovarian SK‐OV‐3 cancer cells (Figure 11 e), despite its very low anticancer activity (IC_50_=1.87 mm).^127^ Cytochrome c is an essential component of the electron transport chain but plays also a decisive role in apoptosis as its release from the mitochondria into the cytosol upon apoptotic stimuli triggers apoptosis.^129^ Thus, POM‐induced overexpression of cytochrome c can promote cell death. p38 is a mitogen‐activated protein kinase (MAPK) that has oncogenesis suppressing properties as it is required for dormancy, that is, inhibition of the cell proliferation upon certain stress stimuli.^130^ Furthermore, Na_7_[Cr^III^Cu^II^W_11_O_39_] is also able to trigger autophagy, which means that two cell death pathways can run in parallel. The same was observed for PM‐17 as it induced apoptotic and autophagic cell death in vitro and in vivo.^18^ The sandwich POM [H_4_{Cu^II^_9_As^III^_6_O_15_(H_2_O)_6_}(As^III^W_9_O_33_)_2_]^8−^ exhibited remarkable activity against cancerous K‐562 (leukemia) and Hep‐G2 (liver) cells (IC_50_=0.4 μm for both) by affecting lysosomes in vitro, which led to the induction of both apoptosis and autophagy.^131^ The autophagy inducing property might originate from the As^III^ containing unit as As_2_O_3_ is known to induce autophagy in leukemia cells by increasing the level of Beclin‐1, a critical regulator of autophagy.^132^

#### 3.2.2 DNA Interaction

One of the main stimuli inducing intrinsic apoptosis is DNA damage. Therefore, the anticancer activity of a series of POMs and POM‐based hybrids was associated with DNA lesions but mainly induced indirectly as the negative charge of both molecules is supposed to prevent direct electrostatic interactions. However, some POM‐based structures were found to directly interact with DNA (Figure 11 f), for example, [(CpTi)_3_SiW_9_O_37_]^7−^ interacts strongly with DNA, most likely via its CpTi moiety.^43^, ^47^ These results were confirmed by the group of Habibi, which investigated the behavior of different CpM‐substituted Keggin POTs (M=Zr^IV^, Ti^IV^, Fe^II^) towards ctDNA and suggested a direct but noncovalent groove or outside stacking binding mode for the POM.^46^ Later, the number of POMs directly interacting with ctDNA was extended, including [PV_2_Mo_10_O_40_]^5−^ and [V_18_O_42_(H_2_O)]^12−^.^31^, ^109^, ^110^, ^111^ Despite the suggested groove or outside stacking interaction, the exact POM binding mechanism remains elusive. Another study investigating the interaction between heptamolybdate [Mo_7_O_24_]^6−^ and the DNA model bis(*p*‐nitrophenyl)phosphate showed that the POM was able to cleave the phosphodiester bond by a yet unknown mechanism.^133^

#### 3.2.3 Inhibition of Angiogenesis

**Figure 12:**
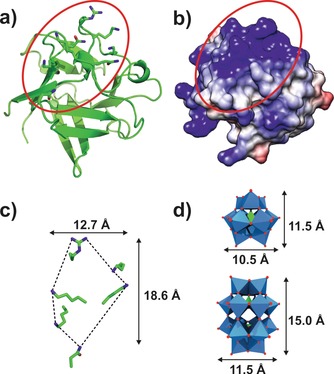
Putative POM‐binding site of bFGF. a) Crystal structure of human bFGF (PDB entry 1BFF^137^) with the putative binding site being marked by a red circle. Side chains of amino acids that potentially contribute to heparin binding are shown as sticks. b) Coulombic surface representation of bFGF is illustrated with blue surfaces representing regions exhibiting a positive potential, whereas gray and red surfaces possess neutral and negative potentials, respectively. c) Zoom view and dimensions of the putative POM‐binding site. d) Polyhedra structures and dimensions of the Keggin and Wells–Dawson anions for easier comparison. Blue polyhedra are {MO_6_}, green polyhedra {XO_4_}, green sticks carbon, dark blue sticks nitrogen, red sticks and spheres oxygen.

Angiogenesis describes the formation of new blood vessels, a vital process in cell growth.^134^ As cancer cells are rapidly dividing, tumors need special blood supply to provide oxygen and other nutrients to continue their abnormal growth. Thus, they induce angiogenesis by activating various growth factors such as the vascular endothelial growth factor (VEGF) and the basic fibroblast growth factor (bFGF), which stimulate the formation of blood vessels. The inhibition of angiogenesis promoting factors is a potential approach in cancer therapy. A series of POMs, including lacunary and fully saturated Keggin, sandwich Keggin, and Wells–Dawson structures, interact strongly with bFGF leading to the inhibition of its proliferation promoting activity in HUVECs.^135^, ^136^ Independent of the charge, Wells–Dawson POMs were most efficient in binding bFGF and therefore it was assumed that the POM structure determines the affinity towards bFGF. Competition assays with suramin, which is known for its angiosuppressive properties, and heparin, an activity enhancer and inhibitor of bFGF, revealed that POMs bind in the vicinity of the heparin binding site, which is rich in positively charged amino acids. According to the dimensions of the proposed binding site, Wells–Dawson structures are supposed to fit better into it than the smaller Keggin ions, which might explain the differences in binding affinity (Figure 12). POM binding might interfere with the ability of bFGF to interact with its receptors, which is required for its angiogenesis promoting function (Figure 11 g).

Mo‐based giant POMs like (NH_4_)_42_[{(Mo^VI^)Mo_5_^VI^O_21_(H_2_O)_6_}_12_{Mo_2_^V^O_4_(CH_3_COO)_30_}] exhibited astonishing anticancer activities against some cancer cell lines with high selectivity towards Hep‐G2 cells (IC_50_=9–55 μg mL^−1^) mainly by inhibiting angiogenesis (Supporting Information, Table S1, described as Mo‐compounds 1–3).^119^ The Mo‐nanoparticles impaired the formation of blood vessels by inhibiting key processes of angiogenesis, namely VEGF‐induced proliferation, migration, and tube formation of endothelial cells (HUVECs) and neovascularization on CAM (chorio‐allantoic membrane). Furthermore, the level of NO, a mediator of angiogenesis, was also significantly reduced in the presence of the POMos as was the VEGF‐induced phosphorylation of signal‐regulated kinase 1 and 2 (ERK1/2) and phosphoinositide 3‐kinase (AKT), signaling processes normally associated with the promotion of angiogenesis (Figure 11 h).

#### 3.2.4 Interaction with Proteins

**Figure 13:**
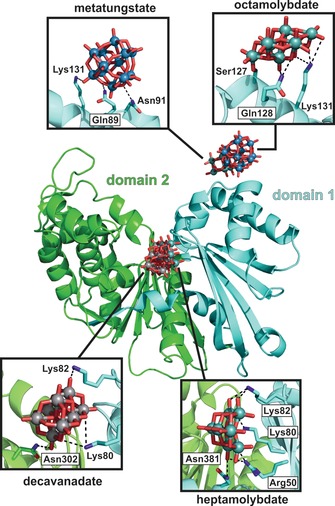
POM‐binding sites of NTPDase1 from *Legionella pneumophila*. The structure of bacterial NTPDase1 consists of two domains (cyan and green cartoon) with the interface forming the active site cleft. The depicted structure is taken from PDB entry 4BVO^142^ as model example. The interacting amino acid residues are shown as sticks. Light blue spheres are tungsten, cyan spheres molybdenum, gray spheres vanadium, cyan/green sticks carbon, dark blue sticks nitrogen, red sticks oxygen. Dashed lines represent POM–protein interactions. Note that octamolybdate forms a covalent bond with a serine (Ser 127).

Owing to their negative charge and their tendency to bind to neutral or hydrophilic surfaces, POMs interact with an array of proteins.^138^ Ectonucleotidases are membrane‐associated enzymes that hydrolyze extracellular nucleotides to the respective nucleosides and are thus involved in numerous physiological and pathological processes.^139^ They can be subdivided into four families, ectonucleoside triphosphate diphosphohydrolases (NTPDases), ectonucleotide pyrophosphatases (NPPs), alkaline phosphatases (APs), and ecto‐5′‐nucleotidase (eN). POMs are potent inhibitors of ectonucleotidases, with some of them being superior to known inhibitors (Figure 11 i; Supporting Information, Table S17).^140^ Crystallographic studies investigating the interaction of decavanadate, metatungstate, octa‐, and heptamolybdate with rat and bacterial NTPDase1 revealed that decavanadate and heptamolybdate bind either at the periphery (rat NTPDase) or deep inside the active site cleft (bacterial NTPDase) of the enzyme at positively charged patches, where they interact with substrate binding residues (Figure 13).^141^, ^142^ Therefore, it was suggested that enzyme inhibition occurs by blocking the nucleotide‐active site interaction. Octamolybdate (Figure 1 i) is located at the entrance of the active site cleft and could also interfere with substrate binding. Interestingly, one of the most potent inhibitors, metatungstate, binds distantly from the active site and therefore the inhibitory effect might result from POM‐mediated destabilization of the entire enzyme structure.

The inhibition of human NTPDase1, also known as CD39, is of particular pharmacological interest as it converts ATP into adenosine, which is an important molecule in the suppression of the antitumor T cell response and therefore it is exploited by tumors to overcome immune response.^143^ NTPDase1 inhibition by metatungstate led to a significant in vivo suppression of murine B16‐F10 melanoma (inhibition rate ca. 86.1 %, 5 mg kg^−1^) and to a moderate reduction of murine colon adenocarcinoma (MCA‐38, ca. 27.3 %, 5 mg kg^−1^) in mice.^144^ Metatungstate was not active in NTPDase1 deficient tumor‐bearing mice confirming NTPDase1 inhibition as the mode of action. Further studies investigating the effect of several POMs on human NTPDase1‐3, NPP1‐3, and rat eN revealed that POMs were most efficient in inhibiting NTPDase1‐3 and NPP1, with some of them exhibiting inhibition constants in the nanomolar range (Supporting Information, Table S17).^145^, ^146^ [Co_4_(H_2_O)_2_(PW_9_O_34_)_2_]^10−^ was identified as a very potent inhibitor of human NTPDase1‐3 (*K*_i_=4, 20 and 100 nm), being the most potent inhibitor of human NTPDase1 described to date.

POMs are also potent inhibitors of tissue specific alkaline phosphatase (TSAP, from calf intestine) and of tissue non‐specific alkaline phosphatase (TNSAP, from pork and human), with some of them being active at nanomolar concentrations (Supporting Information, Table S17).^145^, ^147^ Regarding human TNSAP, [P_6_W_18_O_79_]^20−^ (*K*_i_=2.3 μm) is the most potent inhibitor.^145^ APs are highly expressed by osteoblasts to hydrolyze inorganic pyrophosphate for bone mineralization. This osteoblastic activity is remarkably increased in prostate cancer, which preferentially metastasizes to bone, leading to dysregulated bone formation.^148^ Therefore, the inhibition of APs by POMs could be a promising strategy in the treatment of metastatic prostate cancer.

A series of POMs were tested on several kinases showing high specificity for protein kinase CK2.^149^ Protein kinases catalyze the phosphorylation of proteins leading to their modification, that is, change in enzymatic activity, cellular location, or interaction behavior to regulate cellular processes such as signal transduction. Protein kinase CK2 is upregulated in many cancer types and thus associated with their increased proliferation rate and ability to suppress apoptosis.^150^ Derivates of the Wells–Dawson (IC_50_=1–70 μm) and Preyssler archetype (IC_50_=1–5 μm), and giant POM anions (IC_50_=8–70 μm), showed moderate to high inhibition of CK2. In contrast, smaller POMs such as Keggin derivates (IC_50_=60–1000 μm) were less active indicating that the POM structure roughly determines the inhibitory effect. Further analysis revealed that the POM binding site is located at an exposed site outside the ATP/peptide binding pocket and the catalytic cleft from where the POM could however interfere with the activation loop of CK2 (Supporting Information, Figure S4).^151^

A high‐throughput screening study assaying 400 POMs was performed to identify potential histone deacetylase (HDAC) inhibitors revealing [{(*n*‐Bu)Sn(OH)}_3_GeW_9_O_34_]^4−^ as the most efficient HDAC‐inhibitor (IC_50_=1.1 μm) (Figure 11 j).^48^ HDAC is an enzyme that is responsible for the removal of the acetyl group from an acetylated lysine making histones to wrap DNA more tightly. This is an essential process as DNA expression is regulated by the acetylation and deacetylation of histones. HDACs are involved in cell‐cycle progression and differentiation and therefore disturbances in HDAC encoding genes are linked to tumor development rendering them promising targets.^152^ This rationale was confirmed by the in vitro and in vivo anticancer activity of [{(*n*‐Bu)Sn(OH)}_3_GeW_9_O_34_]^4−^, which were discussed in Section 2.2.1.

Several POMs like [V_10_O_28_]^6−^ or [P_2_W_18_O_62_]^6−^, which are known to exhibit antitumor activity, have also been described as potent P‐type ATPase inhibitors (Figure 11 k).^153^, ^154^, ^155^ P‐type ATPases are a large group of ion pumps that play a crucial role in maintaining the ionic balance in cells and therefore have been described as potential molecular targets for the treatment of several diseases. P‐type ATPases like Na^+^/K^+^‐ATPase also act as signal transducer and their activity was found to be significantly changed in tumors making Na^+^/K^+^‐ATPase inhibitors particularly interesting as anticancer drugs.^156^ Therefore, the reported anticancer activity of some POMs, especially that of decavanadate and other POVs, may partially derive from their ability to inhibit P‐type ATPases. However, POM‐mediated P‐type ATPase inhibition is not tumor‐cell‐selective. Besides this, decavanadate induces mitochondria membrane depolarization and inhibits mitochondrial oxygen consumption in vivo rendering mitochondria the main toxicological target for decavanadate (Figure 11 l).^157^

The mechanism behind the anticancer activity of POM‐bisphosphonate (POM‐BP) complexes, which were discussed in Section 2.2.2, is supposed to be the inhibition of the prenylation of important proteins like the Ras subfamily, which belong to the class of small GTPase. The Ras genes are the most common oncogenes in human cancer, and point mutations within these genes result in the accumulation of activated Ras, which in turn causes a permanent cell growth‐promoting signaling, ultimately leading to cancer.^158^ Therefore, the Ras pathway is a promising biological target. BPs like Zol are known to inhibit farnesyl diphosphate synthase, an enzyme supplying precursors for the biosynthesis of isoprenoids that are essential for prenylation. In the same way, POM‐BP complexes could inhibit the prenylation of (mutated) Ras, preventing it from reaching its full functionality, and thus impairing tumor growth. This mechanism was supported by the finding that the addition of geranylgeraniol (GGOH), an isoprenol that is also used for protein prenylation, impaired the antitumor activity of Mo‐based POM‐BP complexes as prenylation was restored. Interestingly, the activity of V‐containing BP complexes was not affected by GGOH, suggesting that the activity of these complexes is governed by the POV unit and thus follows a POV‐based mode of action.

Besides the discussed biomacromolecules, POMs interact with an array of other important proteins including phosphatases, kinases, polymerases, proteases, actin, sulfotransferases, and sialyltransferases (Figure 11 m).^28^, ^138^, ^159^, ^160^, ^161^, ^162^ POM‐mediated inhibition of certain enzymes has a specific effect on the cell viability and may thus contribute to pharmacological activities. Some of these enzymes are membrane‐associated proteins of which targeting is facilitated as the POM does not has to enter into the cytoplasmic space for inhibition.

#### 3.2.5 Other Mechanisms

The anticancer activity of the {Gd(W_5_O_18_)_2_}–CT_siRNA_ system is on the one hand based on the radiosensitizing effect of the Gd‐POM, leading to the cellular generation of ROS upon X‐ray irradiation and on the other hand by the HIF‐1α down‐regulating effect of the siRNA (Figure 11 n). Besides increasing the ROS level, the POM oxidizes GSH preventing the cancer cell from counteracting the oxidative stress. The inhibition of HIF‐1α leads to the depletion of downstream proteins, which makes the cancer cell more amenable to the lethal hypoxic environment. The expression of the angiogenesis promoting factors VEGF and c‐Met were remarkably decreased upon HIF‐1α inhibition, leading to an increased vulnerability of the cells owing to the detriment of the development of new blood vessels.^100^

The Fe^III^ containing sandwich POM [Fe(HPW_7_O_28_)_2_]^13−^ exhibited moderate in vitro cytotoxicity to a series of cancer cells (Supporting Information, Table S2) but showed promising in vivo activity by inhibiting the tumor growth in S180 sarcoma bearing mice (inhibitory rate=45.7 % at 80 mg kg^−1^).^163^ The in vivo activity derived mainly from the ability of the POM to enhance the immune response in tumor‐bearing mice. The activation of the host immune response has been recognized as a promising approach in the combat against tumors.^164^ The POM significantly increased the proliferation of splenocytes, the activity of natural killer (NK) cells and cytotoxic T lymphocytes (CTLs), and promoted the production of the Type 1 helper cytokines interleukin‐2 (IL‐2) and interferon‐γ (IFN‐γ). Furthermore, the serum antigen‐specific antibody level (IgG2a and IgG2b) was enhanced upon POM treatment. All these results indicate that [Fe(HPW_7_O_28_)_2_]^13−^ has immunomodulatory activity, making this POM a potential drug for immunotherapy (Figure 11 o).

## 4 Summary and Outlook

A series of POMs and POM‐based hybrid systems possess considerable potential as metallodrugs in the treatment of cancer as evidenced by in vitro and in vivo studies. Owing to the lack of clear correlations between the observed anticancer activities and any structural or chemical POM feature, it is quite impossible to anticipate the bioactivity of a given POM. In general, the bioactivity of purely inorganic POVs or vanadium‐containing POMs was higher than that of POMos and POTs. However, the clinical application of POMs is restricted because of some major drawbacks: POMs are generally highly toxic^17^, ^18^, ^22^ and most POM‐archetypes are thermodynamically and kinetically unstable under physiological conditions.^77^, ^106^, ^165^ Furthermore, naked POMs possess rather low cell penetration ability and their promiscuous protein binding leads to low selectivity. Despite these limitations, POMs are still considered valuable bioactive agents, as nearly every molecular property that affects their biological reactivity can be altered and their surface can be organically modified or encapsulated by biocompatible molecules, enabling the synthesis of multifunctional and cell‐penetrating hybrid POMs that overcome most of the aforementioned drawbacks. Grafting of organic molecules onto the POM core or incorporation of bioactive transition metals into the POM framework had partially immense effects on the bioactivity of the system. However, the incorporation of POMs into nanocomposites is clearly more effective in terms of increasing the POM stability and decreasing its inherent toxicity. POM‐based hybrids and nanocomposites containing a cell targeting molecule (for example, a receptor agonist) or a further bioactive compound were especially efficient in most cases. In general, the biological and pharmacokinetic properties of POM‐based nanocomposites seem to be superior to that of just organically functionalized POMs.

Fundamental questions regarding the mechanisms behind the POM‐mediated antiproliferative effects remain largely unanswered. Although a series of possible mechanisms and potential targets were proposed, the exact mode of action remains unclear. With the existing methods it is almost impossible to pinpoint the POM‐induced effect that finally leads to cell death, as cell death is associated with a variety of signaling pathways that crosstalk heavily and involve a plethora of signaling molecules. Due to their multifunctional character, POM‐based systems possess a large repertoire of possibilities to inhibit tumors and therefore their mode of action will probably not be explained by one strict mechanism but rather by multiple interactions interfering with a number of cellular processes. Owing to the low stability of most POM archetypes, more and more researchers propose that monomeric species or unidentified POM fragments are responsible for the observed biological effects.^79^, ^80^, ^92^ According to this, the POM might rather act as a carrier of these active species that upon reaching the site of action releases the toxic payload. Therefore, it is important to develop reliable methods that allow the unambiguous identification of the bioactive species. In this way, whether the activity of POMs correlates with their propensity to dissociate could be investigated. In general, this field requires an immense experimental effort and the development of novel and improved methods to elucidate the mechanism behind the anticancer activity of POMs. Future research will focus on the identification of new targets and the design of novel POM hybrids as they are currently the most efficient (POM‐containing) compounds regarding the biological activity, toxicity, and pharmacokinetic properties. To meet all of these upcoming tasks and to pave the way for POMs as next‐generation anticancer drugs, interdisciplinary cooperation between chemists, biochemists, crystallographers, pharmacists, and physicians is required.

## Conflict of interest

The authors declare no conflict of interest.

## Biographical Information

*Aleksandar Bijelic is currently a postdoctoral research fellow at the Department of Biophysical Chemistry at the University of Vienna (Austria). He received his master's degree in Molecular Life Science in 2012 from the University of Erlangen‐Nürnberg (Germany) and a Ph.D. in Chemistry from the University of Vienna in 2016 (Austria). His research interests include the X‐ray structure analysis of diverse metalloenzymes and the investigation of polyoxometalate‐protein interactions*.

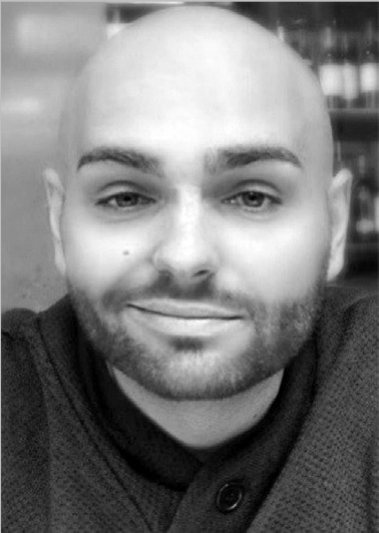

## Biographical Information

*Manuel Aureliano obtained his doctorate at the University of Coimbra. He is an Associate Professor of Biochemistry (Habilitation) and was Director of the Biochemistry degree at the University of Algarve*, *Faro, Portugal (1998–2013). He has published about 80 peer‐reviewed journal articles, reviews and book chapters. He is editor of the book “Vanadium Biochemistry”. His research topics include the role of decavanadate in biology*, *proteins as POM targets, vanadium and diabetes, and antioxidants: toxic and/or beneficial effects*.

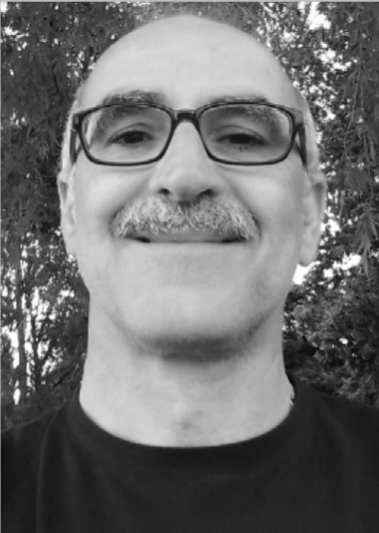

## Biographical Information

*Annette Rompel studied Chemistry at the Westfälische Wilhelms University of Münster where she received her doctoral degree. Besides research at the University of California, Berkeley, and the Lawrence Berkeley National Laboratory, she was a visiting scientist at the RIKEN, Institute of Physical and Chemical Research, Sendai, Japan, and the University of Southern Denmark, Odense. Since 2008, she is the Head of the Department of Biophysical Chemistry at the University of Vienna. Her main research interests are the structure/function elucidation of metalloenzymes and the synthesis and characterization of biologically active polyoxometalates*.

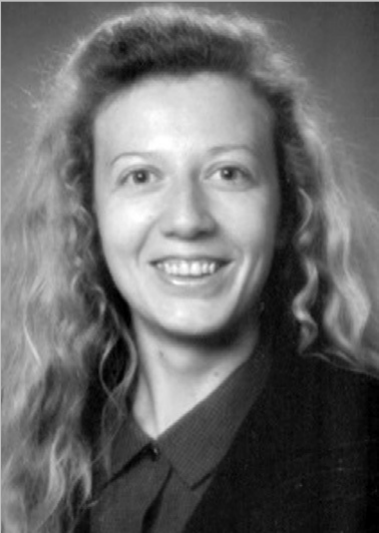

## Supporting information

As a service to our authors and readers, this journal provides supporting information supplied by the authors. Such materials are peer reviewed and may be re‐organized for online delivery, but are not copy‐edited or typeset. Technical support issues arising from supporting information (other than missing files) should be addressed to the authors.

Supplementary
