# Supplementary material for: Polyoxometalates as Potential Next‐Generation Metallodrugs in the Combat Against Cancer
Source: Angew Chem Int Ed Engl. 2018 Oct 12;58(10):2980–99. doi: 10.1002/anie.201803868 (PMC6391951; doi:10.1002/anie.201803868)
Supplement: Supplementary file 1 — Supplementary [file ANIE-58-2980-s001.pdf]

Angewandte Chemie
Eine Zeitschrift der Gesellschaft Deutscher Chemiker

Supporting Information

Polyoxometalates as Potential Next-Generation Metallodrugs in the Combat Against Cancer

*Aleksandar Bijelic, Manuel Aureliano, and Annette Rompel**

anie_201803868_sm_miscellaneous_information.pdf

Table of contents

1. Definition and overview of used cell lines 2
   1.1 Cancerous cell lines 2
   1.2 Normal cell lines 2
2. Anticancer activity of POMs and POM-based hybrids and nanocomposites 3
   2.1 Anticancer activity of purely inorganic POMs 3
   2.2. Anticancer activity of inorganic-organic POM hybrids 14
   2.3. Anticancer activity of POM-based nanocomposites 33
3. Supporting figures 39
4. Potential of polyoxometalates as ectonucleotidase inhibitors 41
5. References 43

1. Definition and overview of used cell lines

1.1 Cancerous cell lines

A2780	human ovarian cancer	MG-63	human bone osteosarcoma
A2780cisR	cisplatin-resistant A2780	MGC-803	human gastric cancer
A-375	human skin melanoma	MKN-45	human gastric cancer
A-549	human lung carcinoma	MM-231	human breast cancer
AsPC-1	human pancreatic cancer	MM-46	murine mammary carcinoma
B16	murine skin melanoma	MX-1	human breast cancer
BCAP	human breast cancer	NCI-H460	human lung cancer
BGC-823	human gastric cancer	OAT	human lung cancer
CADO-ES-1	human Ewing’s sarcoma	OVCAR-3	human ovarian carcinoma
CNE-2	human nasopharyngeal carcinoma	P388	murine leukemia
CO-4	human colon cancer	PC-3	human prostate cancer
H-157	human buccal/mucosa epdidermoid cancer	PC-3*m*	Metastasis-derived variant of PC-3
H22	murine liver cancer	S180	murine sarcoma
HCT-116	human colon cancer	SC-1680	Human gastric cancer
HeLa	Human cervical cancer	SF-268	human glioblastoma
Hep-A-22	murine liver cancer	SGC-7901	human gastric cancer
Hep-G2	human liver cancer	SHEP-SF	human neuroblastoma
HL-60	human promyelocytic leukemia	SHY5Y	human bone narrow neuroblastoma
HLC	human colon cancer	SK-ES-1	human Ewing’s sarcoma
HT-29	human colon adenocarcinoma	SK-OV-3	human ovarian cancer
K-562	human myelogenous leukemia	SMMC-7721	human hepatocellular cancer
KB	oral carcinoma (HeLa derivate)	SW-620	human colon cancer
KCN	human neuroblastoma	U-251	human malignant glioma
MCA-38	murine colon adenocarcinoma	U-87	human brain-like glioblastoma
MCF-7	human breast cancer	U937	human leukemia
MDA-MB-231	human breast adenocarcinoma	UMR-106	rat bone osteosarcoma
Meth A	murine skin sarcoma		

1.2 Normal cell lines

6T CEM[a]	human lymphocytes (leukemia)	HUVEC	human umbilical vein endothelial cells
BMS2	murine none narrow cells	L-02	human hepatic cells
C2C12	murine myoblast cells	MC3T3	murine osteoblast precursor cells
EVC-304	human endothelial cells	MCF-10A	human mammary gland cells
HCEC	human corneal epithelial cells	QSG-7701	human hepatic cells
HEK-293	human embryonic cells	Vero	kidney epithelial cells from grivet
HL-7702	human hepatic cells	WI38	human lung cells

[a] The study using this cell line describes them as normal lymphocytes, however, per definition this cell line is an acute leukemia cell line.

2. Anticancer activity of POMs and POM-based hybrids and nanocomposites

2.1 Anticancer activity of purely inorganic POMs

Table S1. Anticancer activity of purely inorganic polyoxomolybdates (POMos).

POMs	Cell line	Exp.	Activity	Dose (time)[a]	Ref.
$[NH_3Pr^i]_6[Mo_7O_{24}]$ (PM-8)	**Meth A**	**in vivo**	**TWI[b] = 83.0 %**	**100.0 mgkg^{-1} (14 d)**	**[1]**
	**MM-46**	**in vivo**	**TWI[b] = 80.0 %**	**100.0 mgkg^{-1} (14 d)**	**[1]**
	**CO-4**	**in vivo**	**TWI[b] = 54.3 %**	**200.0 mgkg^{-1} (6 d)**	**[2]**
	**MX-1**	**in vivo[c]**	**TWI[b] = 49.0 %**	**200.0 mgkg^{-1} (6 d)**	**[3]**
	**MX-1**	**in vivo[d]**	**TWI[b] = 70.0 %**	**200.0 mgkg^{-1} (30 d)**	**[3]**
	**OAT**	**in vivo[c]**	**TWI[b] = 38.1 %**	**200.0 mgkg^{-1} (6 d)**	**[3]**
	**OAT**	**in vivo[d]**	**TWI[b] = 63.0 %**	**200.0 mgkg^{-1} (30 d)**	**[3]**
	**M-45**	**in vivo**	**TWI[b] ~ 50.0 %**	**200.0 mgkg^{-1} (70 d)**	**[4]**
	M-45	in vitro	IC_{50}[e] = 500.0 µgmL^{-1} (~ 340.0 µM)*	- (48 h)	[4]
	AsPC-1	in vitro	IC_{50}[e] = 500.0 µgmL^{-1} (~ 340.0 µM)*	- (48 h)	[5]
	KB	in vitro	IC_{50}[e] = 0.4 µgmL^{-1} (~ 0.3 µM)*	- (72 h)	[6]
$(NH_4)_6[Mo_7O_{24}]$	**Meth A**	**in vivo**	**TWI[b] = 31.0 %**	**100.0 mgkg^{-1} (14 d)**	**[1]**
	**H22**	**in vivo**	**TWI[b] ~ 25.0 %[h]**	**100.0 mgkg $^{-1}$ (9 d)**	**[7]**
	BGC-823	in vitro	IE[f] = 35.0 %	1.0 mgmL^{-1} (44 h)	[7]
	NCI-H460	in vitro	IC_{50}[e] = 199.0 µM	- (96 h)	[8]
	MCF-7	in vitro	IC_{50}[e] = 283.0 µM	- (96 h)	[8]
	SF-268	in vitro	IC_{50}[e] = 183.0 µM	- (96 h)	[8]
$K_6[Mo_7O_{24}]$	**Meth A**	**in vivo**	**TWI[b] = 50.0 %**	**100.0 mgkg^{-1} (14 d)**	**[1]**
$[(CH_3)_3NH]_6[H_2Mo^{V}_{12}O_{28}(OH)_{12}(Mo^{VI}O_3)_4]$ (PM-17)	**CO-4**	**in vivo**	**TWI[b] = 42.4 %**	**25.0 mgkg^{-1} (6 d)**	**[2]**
	**Meth A**	**in vivo**	**TWI[b] = 45.0 %**	**25.0 mgkg^{-1} (14 d)**	**[1]**
	**AsPC-1**	**in vivo**	**TWI[b] = 33.5 %**	**125.0 µg/body (41 d)**	**[9]**
	AsPC-1	in vitro	IC_{50}[e] = 175.0 µgmL^{-1} (~ 63.0 µM)*	- (24 h)	[9]
	MKN-45	in vitro	IC_{50}[e] = 40.0 µgmL^{-1} (~ 14.4 µM)*	- (24 h)	[9]
$[NH_3Pr^i]_6[Mo_7O_{26}]$ (PM-26)	**CO-4**	**in vivo**	**TWI[b] = 26.5 %**	**50.0 mgkg^{-1} (6 d)**	**[2]**
$(NH_4)_{12}[Fe_2(AsMo_7O_{27})_2]$	Hep-G2	in vitro	IC_{50}[e] = 310.0 µgmL^{-1} (~ 106.8 µM)*	- (n.d.)	[10]
$[Ce(H_2O)_7Cr(OH)_6Mo_6O_{18}]$	Hep-G2	in vitro	IC_{50}[e] = 55.0 µM	- (72 h)	[11]
	MCF-7	in vitro	IC_{50}[e] = 75.0 µM	- (72 h)	[11]
	SK-OV-3	in vitro	IC_{50}[e] = 60.0 µM	- (72 h)	[11]
$[Sm(H_2O)_7Cr(OH)_6Mo_6O_{18}]$	Hep-G2	in vitro	IC_{50}[e] = 130.0 µM	- (72 h)	[11]
	MCF-7	in vitro	IC_{50}[e] = 110.0 µM	- (72 h)	[11]

	SK-OV-3	in vitro	IC_{50}[e] = 240.0 µM	- (72 h)	[11]
$[Eu(H_2O)_7Cr(OH)_6Mo_6O_{18}]$	Hep-G2	in vitro	IC_{50}[e] = 170.0 µM	- (72 h)	[11]
	MCF-7	in vitro	IC_{50}[e] = 165.0 µM	- (72 h)	[11]
	SK-OV-3	in vitro	IC_{50}[e] = 260.0 µM	- (72 h)	[11]
$Na_5[IMo_6O_{24}]$ (PM-32)	**CO-4**	**in vivo**	**TWI[b] = 29.7 %**	**100.0 mgkg^{-1} (6 d)**	[2]
$(NH_4)_3H_6[CoMo_6O_{24}]$	KB	in vitro	IC_{50}[e] = 0.5 µgmL^{-1} (~ 0.4 µM)*	- (72 h)	[6]
$(NH_4)_3H_6[PtMo_6O_{24}]$	KB	in vitro	IC_{50}[e] = 0.5 µgmL^{-1} (~ 0.4 µM)*	- (72 h)	[6]
$(NH_4)_4H_6[NiMo_6O_{24}]$	KB	in vitro	IC_{50}[e] = 0.6 µgmL^{-1} (~ 0.5 µM)*	- (72 h)	[6]
$(NH_4)_2H_6[RhMo_6O_{24}]$	KB	in vitro	IC_{50}[e] = 0.5 µgmL^{-1} (~ 0.4 µM)*	- (72 h)	[6]
$(TBA)_4[Mo_8O_{26}]$	SGC-7901	in vitro	IC_{50}[e] = 100.0 - 400.0 µgmL^{-1} (~ 70.1 - 280.5 µM)*	- (72 h)	[12,13]
	SMMC-7721	in vitro	no activity	200.0 µgmL^{-1} (72 h)	[13]
$Na_3[PMo_{12}O_{40}]$	KB	in vitro	IC_{50}[e] = 3.0 µgmL^{-1} (~ 1.6 µM)*	- (72 h)	[6]
	SGC-7901	in vitro	IC_{50}[e] = 78.0 µgmL^{-1} (~ 41.2 µM)*	- (48 h)	[14]
$Na_4[SiMo_{12}O_{40}]$	KB	in vitro	IC_{50}[e] = 1.9 µgmL^{-1} (~ 1.0 µM)*	- (72 h)	[6]
	A-549	in vitro	IE[f] = 11.0 %	20.0 µM (48 h)	[15]
$Na_4[GeMo_{12}O_{40}]$	KB	in vitro	IC_{50}[e] = 2.7 µgmL^{-1} (~ 1.4 µM)*	- (72 h)	[6]
$Na_3[AsMo_{12}O_{40}]$	KB	in vitro	IC_{50}[e] = 3.5 µgmL^{-1} (~ 1.8 µM)*	- (72 h)	[6]
$Na_4[PVMo_{11}O_{40}]$	KB	in vitro	IC_{50}[e] = 2.1 µgmL^{-1} (~ 0.8 µM)*	- (72 h)	[6]
$Na_5[SiVMo_{11}O_{40}]$	KB	in vitro	IC_{50}[e] = 1.4 µgmL^{-1} (~ 0.7 µM)*	- (72 h)	[6]
$K_6[SiCo(H_2O)Mo_{11}O_{39}]$	MCF-7	in vitro	IC_{50}[e] = 16.2 µM	- (24 h)	[16]
	HEK-293	in vitro	IC_{50}[e] = 11.0 µM	- (24 h)	[16]
	NHF[g]	in vitro	IE[f] = 1.7 %	100.0 µM (24 h)	[16]
$Na_7[CuPMo_{11}O_{40}]$	B16	in vitro	IE[f] = 48.4 %	200.0 µM (24 h)	[17]
$Na_4[AsVMo_{11}O_{40}]$	KB	in vitro	IC_{50}[e] = 2.8 µgmL^{-1} (~ 1.5 µM)*	- (72 h)	[6]

$Na_5[PV_2Mo_{10}O_{40}]$	MCF-7	in vitro	IC_{50}[e] = 20.6 µM	- (24 h)	[18]
	HEK-293	in vitro	IC_{50}[e] = 12.9 µM	- (24 h)	[18]
$K_5[PV_2Mo_{10}O_{40}]$	MCF-7	in vitro	IC_{50}[e] = 199.5 µM	- (24 h)	[19]
$Na_3[PMo_6W_6O_{40}]$	KB	in vitro	IC_{50}[e] = 9.3 µgmL^{-1} (~ 3.8 µM)*	- (72 h)	[6]
$Na_6[P_2Mo_{18}O_{62}]$	KB	in vitro	IC_{50}[e] = 34.5 µgmL^{-1} (~ 11.8 µM)*	- (72 h)	[6]
$Na_6[As_2Mo_{18}O_{62}]$	KB	in vitro	IC_{50}[e] = 52.3 µgmL^{-1} (~ 17.3 µM)*	- (72 h)	[6]
$Na_{12}[P_2W_{15}O_{56}]$	MCF-7	in vitro	IE[f] = 79.0 %	35.0 mgmL^{-1} (24 h)	[20]
	HeLa	in vitro	IE[f] = 85.0 %	35.0 mgmL^{-1} (24 h)	[20]
	Vero[g]	in vitro	IE[f] ~ 37.0 %[h]	35.0 mgmL^{-1} (24 h)	[20]
$(NH_4)_{15}[Na\{(Mo^{V}{}_2O_4)_3(\mu_2\text{-}O)_3(\mu_2\text{-}SO_3)_3(\mu_6\text{-}SO_3)\}_2]$	B16	in vitro	IE[f] = 19.0 %	23.3 µgmL^{-1} (24 h)	[21]
	Vero[g]	in vitro	IE[f] = 14.0 %	23.3 µgmL^{-1} (24 h)	[21]
$Na_6[P_2Mo_5O_{23}]$	SHY5Y	in vitro	IC_{50}[e] = 93.8 µM	- (24 h)	[22]
	EVC-304[g]	in vitro	IC_{50}[e] = 80.7 µM	- (24 h)	[22]
$[K_3Ca(H_2O)_4(HP_2Mo_5O_{23})]$	Hep-G2	in vitro	IC_{50}[e] > 100 µM	- (n.d.)	[23]
	HCT-116	in vitro	IC_{50}[e] > 100 µM	- (n.d.)	[23]
	SMMC-7721	in vitro	IC_{50}[e] > 100 µM	- (n.d.)	[23]
	HL-7702[g]	in vitro	IC_{50}[e] > 100 µM	- (n.d.)	[23]
Mo-compound 1[i]	Hep-G2	in vitro	IC_{50}[e] = 55.0 µgmL^{-1} (~ 2.6 µM)*	- (48 h)	[24]
	A-375	in vitro	IC_{50}[e] = 95.0 µgmL^{-1} (~ 4.5 µM)*	- (48 h)	[24]
	MCF-7	in vitro	IC_{50}[e] > 100.0 µgmL^{-1} (> 4.7 µM)*	- (48 h)	[24]
	CNE-2	in vitro	IC_{50}[e] > 100.0 µgmL^{-1} (> 4.7 µM)*	- (48 h)	[24]
	HUVEC[g]	in vitro	IC_{50}[e] = 43.0 µgmL^{-1} (~ 2.0 µM)*	- (48 h)	[24]
Mo-compound 2[i]	Hep-G2	in vitro	IC_{50}[e] = 13.0 µgmL^{-1} (~ 0.4 µM)*	- (48 h)	[24]
	A-375	in vitro	IC_{50}[e] > 100.0 µgmL^{-1} (> 3.4 µM)*	- (48 h)	[24]
	MCF-7	in vitro	IC_{50}[e] = 86.0 µgmL^{-1} (~ 2.9 µM)*	- (48 h)	[24]
	CNE-2	in vitro	IC_{50}[e] > 100.0 µgmL^{-1} (> 3.4 µM)*	- (48 h)	[24]
	HUVEC[g]	in vitro	IC_{50}[e] = 24.0 µgmL^{-1} (~ 0.8 µM)*	- (48 h)	[24]

Mo-compound 3[i]	Hep-G2	in vitro	IC_{50}[e] = 9.0 μgmL^{-1} (~ 0.5 μM)*	- (48 h)	[24]
	A-375	in vitro	IC_{50}[e] = 78.0 μgmL^{-1} (~ 4.1 μM)*	- (48 h)	[24]
	MCF-7	in vitro	IC_{50}[e] = 78.0 μgmL^{-1} (~ 4.1 μM)*	- (48 h)	[24]
	CNE-2	in vitro	IC_{50}[e] = 83.0 μgmL^{-1} (~ 4.4 μM)*	- (48 h)	[24]
	HUVEC[g]	in vitro	IC_{50}[e] = 17.0 μgmL^{-1} (~ 0.9 μM)*	- (48 h)	[24]

[a] the value in parentheses indicates at which time (after addition of POM) the antitumor effect was measured, that is, in in vivo studies it is the time at which the tumor size was measured (d = days) and in in vitro studies it is the time at which the inhibitory effect was determined (h = hours). [b] TWI = % tumor weight inhibition (in comparison to the control). [c] antitumor effect against human cancer xenografts implanted under subrenal capsule in mice. [d] antitumor effect against human cancer xenografts in nude mice. [e] IC_{50} = the dose required to inhibit or kill 50% of the tested cells. [f] IE = inhibitory effect describing the % inhibition of cells in in vitro studies. [g] these cells are normal noncancerous cells to validate toxicity. [h] only the graph plotting the antitumor activity was provided by the authors and therefore the values were read from the graph. [i] Mo-compound 1 = $(NH_4)_{42}[\{(Mo^{VI})Mo_5^{VI}O_{21}(H_2O)_6\}_{12}\{Mo_2^{V}O_4(CH_3COO)_{30}\}]$, Mo-compound 2 = $(NH_4)_{72-n}[(H_2O)_{81-n}+(NH_4)_n\subset\{(Mo^{VI})Mo_5^{VI}O_{21}(H_2O)_6\}_{12}\{Mo_2^{V}O_4(SO_4)_{30}\}]$ and Mo-compound 3 = $(CH_6N_3)_{22}[(CH_6N_3)_{20}\subset\{(Mo^{VI})Mo_5^{VI}O_{21}(H_2O)_6\}_{12}\{Mo_2^{V}O_4(CH_3COO)_{30}\}]$. * the values in parentheses indicate the IC_{50} value in μM unit, note that this is a rough estimation as in most cases the water content of the structure was not provided by the authors and therefore the molecular weight of the respective POM could not be defined accurately. NH_3Pr^i = isopropylammonium, Pr = propyl, TBA = tetra-*n*-butyl ammonium, n.d. = not defined. **In vivo results are highlighted in green**.

Table S2. Anticancer activity of purely inorganic polyoxotungstates (POTs), polyoxovanadotungstates and polyoxomolybdotungstates.

POMs	Cell line	Exp.	Activity	Dose (time)[a]	Ref.
$Na_6[H_2W_{12}O_{40}]$	**B16-F10**	**in vivo**	**TWI[b] ~ 86.1 %[f]**	**5.0 mgkg^{-1} (10 d)**	**[25]**
	**MCA-38**	**in vivo**	**TWI[b] ~ 27.3 %[f]**	**5.0 mgkg^{-1} (10 d)**	**[25]**
	H-157	in vitro	IE[c] = 38.0 %	0.1 µM (24 h)	[26]
	HCEC[d]	in vitro	IE[c] < 10.0 %	100.0 µM (24 h)	[26]
$Na_{10}[H_2W_{12}O_{42}]$	H-157	in vitro	IE[c] ~ 77.0 %[f]	0.1 µM (24 h)	[26]
	HCEC[d]	in vitro	IE[c] < 10.0 %	100.0 µM (24 h)	[26]
$Na_6[Cu_2(H_2O)_2(H_2W_{12}O_{42})]$	SHEP-SF	in vitro	IC_{50}[e] = 25.0 µM	- (72 h)	[27]
	KCN	in vitro	IC_{50}[e] = 8.4 µM	- (72 h)	[27]
	CADO-ES	in vitro	IC_{50}[e] = 31.8 µM	- (72 h)	[27]
$H_3[PW_{12}O_{40}]$	PC-3	in vitro	IE[c] ~ 55.0 %[f]	50.0 µgmL^{-1} (72 h)	[28]
	Hep-G2	in vitro	no activity	n.d. (72 h)	[28]
	HeLa	in vitro	no activity	n.d. (72 h)	[28]
$Na_3[PW_{12}O_{40}]$	KB	in vitro	IC_{50}[e] = 43.5 µgmL^{-1} (~ 14.8 µM)*	- (72 h)	[6]
$K_5[BW_{12}O_{40}]$	SMMC-7721	in vitro	IC_{50}[e] = 858.0 µM	- (n.d.)	[29]
$(TBA)_4[SiW_{12}O_{40}]$	U-87	in vitro	IE[c] ~ 13.0 %[f]	50.0 µgmL^{-1} (48 h)	[30]
$(TBA)_4[GeW_{12}O_{40}]$	U-87	in vitro	IE[c] = 25.0 %	50.0 µgmL^{-1} (48 h)	[30]
$K_6H_2[CoTiW_{11}O_{40}]$	SSMC-7721	in vitro	IC_{50}[e] = 7.1 µM	- (72 h)	[31]
	HeLa	in vitro	IC_{50}[e] = 14.1 µM	- (72 h)	[31]
	HeLa	in vitro	IC_{50}[e] = 19.8 µgmL^{-1} (~ 6.1 µM)*	- (72 h)	[32]
	HeLa	in vitro	IE[e] ~ 10.0 %[f]	50.0 µg (24 h)	[33]
	HL-60	in vitro	IC_{50}[e] = 20.9 µgmL^{-1} (~ 6.5 µM)*	- (72 h)	[32]
$Na_6[TeW_6O_{24}]$	HeLa	in vitro	IE[d] = 69.0 %	10.0 µM (48 h)	[49]
	Vero	in vitro	IE[d] = 16.0 %	10.0 µM (48 h)	[49]
$K_6Na_2[SiW_{11}O_{39}]$	S180	in vitro	IE[e] = 45.6 %	54.3 µgmL^{-1} (n.d.)	[34]
	leucocythemia	in vitro	IE[e] = 69.0 %	54.3 µgmL^{-1} (n.d.)	[34]
$K_6[Co(H_2O)SiW_{11}O_{39}]$	MCF-7	in vitro	IC_{50}[e] = 6.4 µM	- (24 h)	[35]
	HEK-293	in vitro	IC_{50}[e] = 6.2 µM	- (24 h)	[35]
$Na_7[CrCuW_{11}O_{39}]$	SK-OV-3	in vitro	IC_{50}[e] = 1900.0 µM	- (12 h)	[36]
α-$Na_{10}[PV(H_2O)W_{11}O_{37}]$	MCF-7	in vitro	IC_{50}[e] > 1000.0 µM	- (24 h)	[19]
α-$K_5[SiVW_{11}O_{40}]$	MCF-7	in vitro	IC_{50}[e] > 1000.0 µM	- (24 h)	[19]
$Na_3[PMo_3W_9O_{40}]$	KB	in vitro	IC_{50}[e] = 25.6 µgmL^{-1}	- (72 h)	[6]

			(~ 9.5 µM)*		
α-1,2,3,-$K_6H[SiV_3W_9O_{40}]$	MCF-7	in vitro	IC_{50}[e] > 1000.0 µM	- (24 h)	[19]
α-$Na_{10}[PV_3(H_2O)W_9O_{37}]$	MCF-7	in vitro	IC_{50}[e] = 315.8 µM	- (24 h)	[19]
$(TBA)_4[SiMo_3W_9O_{40}]$	U-87	in vitro	IE[c] ~ 19.0 %[f]	50.0 µgmL^{-1} (48 h)	[30]
$(TBA)_4[GeMo_3W_9O_{40}]$	U-87	in vitro	IE[c] = 29.0 %	50.0 µgmL^{-1} (48 h)	[30]
$(TBA)_4H_3[SiV_3W_9O_{40}]$	U-87	in vitro	IE[c] ~ 29.0 %[f]	50.0 µgmL^{-1} (48 h)	[30]
$(TBA)_4H_3[GeV_3W_9O_{40}]$	U-87	in vitro	IE[c] = 44.0 %	50.0 µgmL^{-1} (48 h)	[30]
α-$Na_9[AsW_9O_{33}]$	K-562	in vitro	IE[c] = 29.4 %	10.0 µM (48 h)	[37]
β-$Na_9[AsW_9O_{33}]$	HeLa	in vitro	IC_{50}[e] = 166.0 µgmL^{-1} (~ 39.8 µM)*	- (48 h)	[38]
$Na_5K_7[\{β\text{-}SiCo_2W_{10}O_{36}(OH)_2(H_2O)\}_2]$	SMMC-7721	in vitro	IC_{50}[e] = 104.9 µgmL^{-1} (~ 16.6 µM)*	- (n.d.)	[39]
	SK-OV-3	in vitro	IC_{50}[e] = 76.4 µgmL^{-1} (~ 12.1 µM)*	- (n.d.)	[39]
	6T CEM[d]	in vitro	IC_{50}[e] = 1768.0 µgmL^{-1} (~ 278.0 µM)*	- (n.d.)	[39]
	BMS2[d]	in vitro	IC_{50}[e] = 2878.0 µgmL^{-1} (~ 455.7 µM)*	- (n.d.)	[39]
$K_{11}[Y(PW_{11}O_{39})_2]$	HeLa	in vitro	IC_{50}[e] = 128.0 µM	- (72 h)	[40]
	Hep-G2	in vitro	IC_{50}[e] = 55.5 µM	- (72 h)	[40]
	HEK-293[d]	in vitro	IC_{50}[e] = 71.3 µM	- (72 h)	[40]
$K_{11}[La(PW_{11}O_{39})_2]$	HeLa	in vitro	IC_{50}[e] = 378.0 µM	- (72 h)	[40]
	Hep-G2	in vitro	IC_{50}[e] = 62.1 µM	- (72 h)	[40]
	HEK-293[d]	in vitro	IC_{50}[e] = 74.6 µM	- (72 h)	[40]
$K_{10}[Co_4(H_2O)_2(PW_9O_{34})_2]$	HeLa	in vitro	IC_{50}[e] = 160.0 µM	- (24 h)	[41]
$Na_9[SbW_9O_{33}]$	SK-OV-3	in vitro	IC_{50}[e] = 203.6 µgmL^{-1} (~ 48.2 µM)*	- (72 h)	[42]
	SMMC-7721	in vitro	IC_{50}[e] = 219.6 µgmL^{-1} (~ 52.0 µM)*	- (72 h)	[42]
	Hep-G2	in vitro	IC_{50}[e] = 214.0 µgmL^{-1} (~ 50.7 µM)*	- (72 h)	[42]
$Na_9[\{Na(H_2O)_2\}_3\{Co(H_2O)\}_3(α\text{-}B\text{-}SbW_9O_{33})_2]$	SK-OV-3	in vitro	IC_{50}[e] = 33.3 µgmL^{-1} (~ 5.8 µM)*	- (72 h)	[42]
	SMMC-7721	in vitro	IC_{50}[e] = 20.7 µgmL^{-1} (~ 3.6 µM)*	- (72 h)	[42]
	Hep-G2	in vitro	IC_{50}[e] = 9.3 µgmL^{-1} (~ 1.6 µM)*	- (72 h)	[42]
	L02[d]	in vitro	IC_{50}[e] = 63.2 µgmL^{-1}	- (72 h)	[42]

			(~ 11.0 µM)*		
$Na_{12}[Cu_3(H_2O)_3(\alpha\text{-}AsW_9O_{33})_2]$	K-562	in vitro	IC_{50}[e] = 2.7 µM	- (48 h)	[37]
	Hep-G2	in vitro	IC_{50}[e] = 4.3 µM	- (48 h)	[37]
$Na_8[H_4\{Cu_9As_6O_{15}(H_2O)_6\}(\alpha\text{-}AsW_9O_{33})_2]$	K-562	in vitro	IC_{50}[e] = 0.4 µM	- (48 h)	[37]
	Hep-G2	in vitro	IC_{50}[e] = 0.4 µM	- (48 h)	[37]
	QSG-7701[d]	in vitro	IC_{50}[e] = 1.7 µM	- (48 h)	[37]
$K_7Na_3[Cu_4(H_2O)_2(PW_9O_{34})_2]$	MG-63	in vitro	IC_{50}[e] = 22.0 µM	- (24 h)	[43]
	UMR-106	in vitro	IC_{50}[e] = 81.0 µM	- (24 h)	[43]
	MC3T3-E1[d]	in vitro	IC_{50}[e] = 92.0 µM	- (24 h)	[43]
$\alpha\text{-}K_8H_6[Si_2Ti_6W_{18}O_{77}]$	**H22**	**in vivo**	**TWI[b] = 40.0 %**	**200.0 $mgkg^{-1}$ (11 d)**	**[44]**
	HL-60	in vitro	IC_{50}[e] = 60.5 $µgmL^{-1}$ (~ 11.6 µM)*	- (n.d.)	[44]
	HL-60	in vitro	IC_{50}[e] = 23.3 $µgmL^{-1}$ (~ 4.5 µM)*	- (n.d.)	[45]
	HeLa	in vitro	IC_{50}[e] = 46.4 $µgmL^{-1}$ (~ 8.9 µM)*	- (n.d.)	[44]
	HeLa	in vitro	IC_{50}[e] = 26.3 $µgmL^{-1}$ (~ 5.1 µM)*	- (n.d.)	[45]
α-and β-$[Ge_2Ti_6W_{18}O_{77}]^{14-}$	SSMC-7721	in vitro	no activity	10.0 $µgmL^{-1}$ (72 h)	[31]
	HeLa	in vitro	no activity	10.0 $µgmL^{-1}$ (72 h)	[31]
$(Him)_2[(W(OH)_2)_2(Mn(H_2O)_3)_2(Na_3(H_2O)_{14})(BiW_9O_{33})_2]$	SGC-7901	in vitro	IC_{50}[e] = 81.0 µM	- (24 h)	[46]
	Hep-G2	in vitro	IC_{50}[e] = 30.1 µM	- (24 h)	[47]
	QSG-7701[d]	in vitro	IC_{50}[e] = 43.2 µM	- (24 h)	[47]
$(H_2im)_2[(W(OH)_2)_2(Co(H_2O)_3)_2Na_4(H_2O)_{14})(BiW_9O_{33})_2]$	Hep-G2	in vitro	IC_{50}[e] = 37.3 µM	- (24 h)	[47]
	QSG-7701[d]	in vitro	IC_{50}[e] = 38.5 µM	- (24 h)	[47]
$(H_2im)_2[(W_{0.5}Ni_{0.5}(H_2O))_2(Ni(H_2O)_3)_2Na_4(H_2O)_{14})(BiW_9O_{33})_2]$	Hep-G2	in vitro	IC_{50}[e] = 25.6 µM	- (24 h)	[47]
	QSG-7701[d]	in vitro	IC_{50}[e] = 32.4 µM	- (24 h)	[47]
$(H_2im)_2[(WO(OH))_2(Zn(H_2O)_3)_2Na_4(H_2O)_{13}(BiW_9O_{33})_2]$	Hep-G2	in vitro	IC_{50}[e] = 32.3 µM	- (24 h)	[47]
	QSG-7701[d]	in vitro	IC_{50}[e] = 49.7 µM	- (24 h)	[47]
$[(CH_3)_4N]_2Na_{6.5}(NH_4)_2[Sn_{1.5}(WO_2(OH))_{0.5}(WO_2)_2(SbW_9O_{33})_2]$	SHEP-SF	in vitro	IC_{50}[e] = 24.2 µM	- (72 h)	[27]
	KCN	in vitro	IC_{50}[e] = 0.8 µM	- (72 h)	[27]
	CADO-ES	in vitro	IC_{50}[e] = 1.4 µM	- (72 h)	[27]
$Na_{11}(NH_4)[(Mn^{II}(H_2O))_3(SbW_9O_{33})_2]$	SHEP-SF	in vitro	IC_{50}[e] = 9.8 µM	- (72 h)	[27]
	KCN	in vitro	IC_{50}[e] = 8.1 µM	- (72 h)	[27]
	CADO-ES	in vitro	IC_{50}[e] = 6.9 µM	- (72 h)	[27]
$Na_5K_7[(V^{IV}O)_3(AsW_9O_{33})_2]$	SHEP-SF	in vitro	IC_{50}[e] = 30.6 µM	- (72 h)	[27]
	KCN	in vitro	IC_{50}[e] = 6.4 µM	- (72 h)	[27]
	CADO-ES	in vitro	IC_{50}[e] = 6.7 µM	- (72 h)	[27]
$[(CH_3)_4N]_2Na_7[(Mn^{III}(H_2O))_3(SbW_9O_{33})_2]$	SHEP-SF	in vitro	IC_{50}[e] = 34.7 µM	- (72 h)	[27]

	KCN	in vitro	IC_{50}[e] = 8.1 µM	- (72 h)	[27]
	CADO-ES	in vitro	IC_{50}[e] = 75.0 µM	- (72 h)	[27]
$Na_{10}[Mn^{II}_2(H_2O)_6(WO_2)_2(SbW_9O_{33})_2]$	SHEP-SF	in vitro	IC_{50}[e] = 27.6 µM	- (72 h)	[27]
	KCN	in vitro	IC_{50}[e] = 10.0 µM	- (72 h)	[27]
	CADO-ES	in vitro	IC_{50}[e] = 6.7 µM	- (72 h)	[27]
$K_6[P_2W_{18}O_{62}]$	Hep-G2	in vitro	IC_{50}[e] = 24.0 µM	- (72 h)	[11]
	MCF-7	in vitro	IC_{50}[e] = 22.0 µM	- (72 h)	[11]
	SK-OV-3	in vitro	IC_{50}[e] = 19.0 µM	- (72 h)	[11]
$Na_6[TeW_6O_{24}]$	H-157	in vitro	IE[c] ~ 80.0 %[f]	0.1 µM (24 h)	[26]
	HCEC[d]	in vitro	IE[c] < 10.0 %	100.0 µM (24 h)	[26]
$M_{14}[NaP_5W_{30}O_{110}]$	HT-29	in vitro	IC_{50}[e] = 3.6 µM	- (36 h)	[48]
(M_{14} = $K_{12.5}Na_{1.5}$ [48], $(NH_4)_{14}$ [49])	HeLa	in vitro	IE[c] = 74.0 %	10.0 µM (48 h)	[49]
	HUVEC[d]	in vitro	IC_{50}[e] = 34.2 µM	- (36 h)	[48]
	Vero[d]	in vitroTi6	IE[c] = 15.0 %	10.0 µM (48 h)	[49]
$Na_{12}H[Fe(HPW_7O_{28})_2]$	**S180**	**in vivo**	**TWI[b] = 38.9 %**	**40.0 mgkg^{-1} (9 d)**	**[50]**
	A-549	in vitro	IC_{50}[e] = 150.0 µM	- (44 h)	[50]
	HCI-H460	in vitro	IC_{50}[e] = 141.5 µM	- (44 h)	[50]
	Hep-G2	in vitro	IC_{50}[e] = 63.8 µM	- (44 h)	[50]
	K-562	in vitro	IC_{50}[e] = 114.6 µM	- (44 h)	[50]
$Na_{16}[(O_3POPO_3)_4W_{12}O_{36}]$	H-157	in vitro	IE[c] = 65.0 %	0.1 µM (24 h)	[26]
	HCEC[d]	in vitro	IE[c] < 10.0 %	100.0 µM (24 h)	[26]
$Na_{20}[P_6W_{18}O_{79}]$	H-157	in vitro	IE[c] = 56.0 %	0.1 µM (24 h)	[26]
	HCEC[d]	in vitro	IE[c] < 10.0 %	100.0 µM (24 h)	[26]
$(Him)_2[(W(OH)_2)_2Co_2(H_2O)_6Na_4(H_2O)_{14}(BiW_9O_{33})_2]$	HT-29	in vitro	IE[c] = 46.9 %	80.0 µM (24 h)	[51]
	SGC-7901	in vitro	IE[c] ~ 72.0 %[f]	80.0 µM (24 h)	[51]
	Hep-G2	in vitro	IE[c] ~ 40.0 %[f]	80.0 µM (24 h)	[51]
$(NH_4)_{18}[NaSb_9W_{21}O_{86}]$	HT-29	in vitro	IC_{50}[e] > 50.0 µM	- (72 h)	[52]
	A2780	in vitro	IC_{50}[e] = 4.4 µM	- (72 h)	[52]
	A2780cisR	in vitro	IC_{50}[e] = 29.0 µM	- (72 h)	[52]
	OVGAR-3	in vitro	IC_{50}[e] = 8.8 µM	- (72 h)	[52]
	SK-OV-3	in vitro	IC_{50}[e] > 50.0 µM	- (72 h)	[52]
	CT-26	in vitro	IC_{50}[e] > 50.0 µM	- (72 h)	[52]
	A-549	in vitro	IC_{50}[e] > 50.0 µM	- (72 h)	[52]
	MCF-7	in vitro	IC_{50}[e] = 43.7 µM	- (72 h)	[52]
	HEK-293[d]	in vitro	IC_{50}[e] = 34.5 µM	- (72 h)	[52]
$K_{23}H_2[TbAs_4W_{40}O_{140}]$	**S180**	**in vivo**	**TWI[b] = 37.2 %**	**1.0 mgkg^{-1} (11 d)**	**[53]**
	B16	in vitro	IE[c] = 67.5 %	125.0 µgmL^{-1} (n.d.)	[53]
	H22	in vitro	IE[c] = 71.4 %	125.0 µgmL^{-1} (n.d.)	[53]
	HL-60	in vitro	IE[c] = 35.0 %	20.0 µgmL^{-1} (n.d.)	[53]
	Rectum cancer	in vitro	IE[c] = 65.0 %	20.0 µgmL^{-1} (n.d.)	[53]

$K_{23}H_2[EuAs_4W_{40}O_{140}]$	Breast cancer	in vitro	IE[c] = 57.0 %	5.0 µgmL^{-1} (n.d.)	[53]
$Na_{22}Cs_3[Cs\subset Eu_6As_6W_{63}O_{218}(H_2O)_{14}(OH)_4]$	MCF-7	in vitro	IC_{50}[e] = 608.0 µgmL^{-1} (~ 34.2 µM)*	- (24 h)	[54]
	KB	in vitro	IC_{50}[e] = 522.0 µgmL^{-1} (~ 29.3 µM)*	- (24 h)	[54]
	PC-3	in vitro	IC_{50}[e] = 613.0 µgmL^{-1} (~ 34.5 µM)*	- (24 h)	[54]
	A-549	in vitro	IC_{50}[e] = 629.0 µgmL^{-1} (~ 35.4 µM)*	- (24 h)	[54]

[a] the value in parentheses indicates at which time (after addition of POM) the antitumor effect was measured, that is, in in vivo studies it is the time at which the tumor size was measured (d = days) and in in vitro studies it is the time at which the inhibitory effect was determined (h = hours). [b] TWI = % tumor weight inhibition (in comparison to the control). [c] IE = inhibitory effect describing the % inhibition of cells in in vitro studies. [d] these cells are normal noncancerous cells to validate toxicity. [e] IC_{50} = the dose required to inhibit or kill 50% of the tested cells. [f] only the graph plotting the antitumor activity was provided by the authors and therefore the values were read from the graph. * the values in parentheses indicate the IC_{50} value in µM unit, note that this is a rough estimation as in most cases the water content of the structure was not provided by the authors and therefore the molecular weight of the respective POM could not be defined accurately. TBA = tetra-*n*-butyl ammonium, im = imidazole., n.d. = not defined. **In vivo results are highlighted in green**.

Table S3. Anticancer activity of purely inorganic polyoxovanadates (POVs) and polyoxoniobates (PONbs)

POMs	Cell line	Exp.	Activity	Dose (time)[a]	Ref.
$Na_6[V_{10}O_{28}]$	NCI-H460	in vitro	IC_{50}[a] = 0.2 µM	- (96 h)	[55]
	MCF-7	in vitro	IC_{50}[b] = 0.3 µM	- (96 h)	[55]
	SF-268	in vitro	IC_{50}[b] = 0.3 µM	- (96 h)	[55]
	SMMC-7721	in vitro	IC_{50}[b] = 9.6 µgmL^{-1} (~ 7.5 µM)*	- (72 h)	[56]
	SK-OV-3	in vitro	IC_{50}[b] = 18.9 µgmL^{-1} (~ 14.7 µM)*	- (72 h)	[56]
	HeLa	in vitro	IE[c] = 66.0 %	10.0 µM (n.d.)	[49]
	Vero[d]	in vitro	IE[c] = 16.0 %	10.0 µM (n.d.)	[49]
$Na_4Co(H_2O)_6[V_{10}O_{28}]$	**Hep-A-22**	**in vivo**	**TWI[e] = 47.1 %**	**6.0 mgkg^{-1} (10 d)**	**[56]**
	SMMC-7721	in vitro	IC_{50}[b] = 0.3 µgmL^{-1} (~ 0.2 µM)*	- (72 h)	[56]
	SK-OV-3	in vitro	IC_{50}[b] = 0.2 µgmL^{-1} (~ 0.1 µM)*	- (72 h)	[56]
$K_7[NiV_{13}O_{38}]$	KB	in vitro	IC_{50}[b] = 0.4 µgmL^{-1} (~ 0.2 µM)*	- (72 h)	[6]
	HCT	in vitro	IC_{50}[b] = 0.6 µgmL^{-1} (~ 0.4 µM)*	- (72 h)	[6]
	BEL	in vitro	IC_{50}[b] = 0.8 µgmL^{-1} (~ 0.5 µM)*	- (72 h)	[6]
	B16	in vitro	IC_{50}[b] = 0.9 µgmL^{-1} (~ 0.6 µM)*	- (72 h)	[6]
	BCAP	in vitro	IC_{50}[b] = 0.9 µgmL^{-1} (~ 0.6 µM)*	- (72 h)	[6]
	ESCL	in vitro	IC_{50}[b] = 0.7 µgmL^{-1} (~ 0.4 µM)*	- (72 h)	[6]
$Pr_2H[NiV_{13}O_{38}]$	KB	in vitro	IC_{50}[b] = 0.3 µgmL^{-1} (~ 0.2 µM)*	- (72 h)	[6]
	HCT	in vitro	IC_{50}[b] = 0.4 µgmL^{-1} (~ 0.3 µM)*	- (72 h)	[6]
	BEL	in vitro	IC_{50}[b] = 0.8 µgmL^{-1} (~ 0.6 µM)*	- (72 h)	[6]
	B16	in vitro	IC_{50}[b] = 0.8 µgmL^{-1} (~ 0.6 µM)*	- (72 h)	[6]
	BCAP	in vitro	IC_{50}[b] = 0.9 µgmL^{-1} (~ 0.6 µM)*	- (72 h)	[6]
$K_7[MnV_{13}O_{38}]$	KB	in vitro	IC_{50}[b] = 0.5 µgmL^{-1} (~ 0.3 µM)*	- (72 h)	[6]
$K_9[PV_{14}O_{42}]$	KB	in vitro	IC_{50}[b] = 1.3 µgmL^{-1} (~ 0.7 µM)*	- (72 h)	[6]

$K_{12}[V_{18}O_{42}(H_2O)]$	MCF-7	in vitro	IC_{50}[b] = 12.0 µM	- (48 h)	[19]
	MDA-MB-231	in vitro	IC_{50}[b] = 360.3 µM	- (48 h)	[19]
$K_7H[Nb_6O_{19}]$	K-562	in vitro	IC_{50}[b] > 100.0 µM	- (48 h)	[57]
	SGC-7901	in vitro	IC_{50}[b] = 14.4 µM	- (48 h)	[58]

[a] the value in parentheses indicates at which time (after addition of POM) the antitumor effect was measured, that is, in in vivo studies it is the time at which the tumor size was measured (d = days) and in in vitro studies it is the time at which the inhibitory effect was determined (h = hours). [b] IC_{50} = the dose required to inhibit or kill 50% of the tested cells. [c] IE = inhibitory effect describing the % inhibition of cells in in vitro studies. [d] these cells are normal noncancerous cells to validate toxicity. [e] TWI = % tumor weight inhibition (in comparison to the control). * the values in parentheses indicate the IC_{50} value in µM unit, note that this is a rough estimation as in most cases the water content of the structure was not provided by the authors and therefore the molecular weight of the respective POM could not be defined accurately. Pr = propyl, n.d. = not defined. **In vivo results are highlighted in green**.

2.2. Anticancer activity of inorganic-organic POM hybrids

Table S4. Anticancer activity of organometallo substituted POMs.

Organometallo subst. POM	Cell line	Exp.	Activity	Dose (time)[a]	Ref.
α-$K_3H_2[(C_4H_7O_2Sn)SiW_{11}O_{39}]$	SSMC-7721	in vitro	IC_{50}[b] = 53.3 µgmL^{-1}<br>(~ 16.9 µM)[c]	- (72 h)	[59]
	HeLa	in vitro	IC_{50}[b] = 49.6 µgmL^{-1}<br>(~ 15.7 µM)[c]	- (72 h)	[59]
β$_2$-$K_3H_2[(C_4H_7O_2Sn)SiW_{11}O_{39}]$	SSMC-7721	in vitro	IC_{50}[b] = 76.2 µgmL^{-1}<br>(~ 23.8 µM)[c]	- (72 h)	[59]
	HeLa	in vitro	IC_{50}[b] = 77.1 µgmL^{-1}<br>(~ 24.1 µM)[c]	- (72 h)	[59]
α-$K_3H_2[(C_5H_9O_2Sn)SiW_{11}O_{39}]$	SSMC-7721	in vitro	IC_{50}[b] = 49.9<br>(~ 15.6 µM)[c]	- (72 h)	[59]
	HeLa	in vitro	IC_{50}[b] = 47.5<br>(~ 14.9 µM)[c]	- (72 h)	[59]
β$_2$-$K_3H_2[(C_5H_9O_2Sn)SiW_{11}O_{39}]$	SSMC-7721	in vitro	IC_{50}[b] = 76.8 µgmL^{-1}<br>(~ 23.9 µM)[c]	- (72 h)	[59]
	HeLa	in vitro	IC_{50}[b] = 75.6 µgmL^{-1}<br>(~ 23.5 µM)[c]	- (72 h)	[59]
$K_4[(C_4H_7O_2Sn)PW_{11}O_{39}]$	SSMC-7721	in vitro	IC_{50}[b] = 70.1 µgmL^{-1}<br>(~ 21.5 µM)[c]	- (72 h)	[60]
	HeLa	in vitro	IC_{50}[b] = 82.6 µgmL^{-1}<br>(~ 25.4 µM)[c]	- (72 h)	[60]
$K_4[(C_5H_9O_2Sn)PW_{11}O_{39}]$	SSMC-7721	in vitro	IC_{50}[b] = 68.3 µgmL^{-1}<br>(~ 20.8 µM)[c]	- (72 h)	[60]
	HeLa	in vitro	IC_{50}[b] = 82.0 µgmL^{-1}<br>(~ 24.9 µM)[c]l	- (72 h)	[60]
$[(C_4H_7O_2Sn)GaW_{11}O_{39}]^{9-}$	SSMC-7721	in vitro	IC_{50}[b] = 15.7 µM	- (72 h)	[31]
$[(C_4H_7O_2Sn)BW_{11}O_{39}]^{9-}$	SSMC-7721	in vitro	IC_{50}[b] = 17.7 µM	- (72 h)	[31]
$[(C_4H_7O_2Sn)GeW_{11}O_{39}]^{8-}$	SSMC-7721	in vitro	IC_{50}[b] = 26.2 µM	- (72 h)	[31]
γ-$((CH_3)_4N)_4H_2[(C_4H_7O_2Sn)_2SiW_{10}O_{38}]$	SSMC-7721	in vitro	IC_{50}[b] = 64.4 µgmL^{-1}<br>(~ 20.2 µM)[c]	- (72 h)	[61]
	HeLa	in vitro	IC_{50}[b] = 78.1 µgmL^{-1}<br>(~ 24.5 µM)[c]	- (72 h)	[61]
γ-$((CH_3)_4N)_4H_2[(C_5H_9O_2Sn)_2SiW_{10}O_{38}]$	SSMC-7721	in vitro	IC_{50}[b] = 60.9 µgmL^{-1}<br>(~ 19.0 µM)[c]	- (72 h)	[61]
	HeLa	in vitro	IC_{50}[b] = 74.4 µgmL^{-1}<br>(~ 23.2 µM)[c]	- (72 h)	[61]

γ-$((CH_3)_4N)_4H_2[(NC_3H_4Sn)_2SiW_{10}O_{38}]$	SSMC-7721	in vitro	IC_{50}[b] = 50.1 µgmL^{-1} (~ 15.7 µM)[c]	- (72 h)	[61]
	HeLa	in vitro	IC_{50}[b] = 56.7 µgmL^{-1} (~ 17.8 µM)[c]	- (72 h)	[61]
K_8γ-$[SiW_{10}O_{36}]$	SSMC-7721	in vitro	IC_{50}[b] = 287.8 µM	- (n.d.)	[39]
	SK-OV-3	in vitro	IC_{50}[b] = 244.6 µM	- (n.d.)	[39]
$K_5[(C_4H_7O_2Sn)_2PW_{10}O_{38}]$	SSMC-7721	in vitro	IC_{50}[b] = 56.2 µgmL^{-1} (~ 17.2 µM)[c]	- (72 h)	[62]
	HeLa	in vitro	IC_{50}[b] = 70.9 µgmL^{-1} (~ 21.7 µM)[c]	- (72 h)	[62]
$K_5[(C_5H_9O_2Sn)_2PW_{10}O_{38}]$	SSMC-7721	in vitro	IC_{50}[b] = 55.7 µgmL^{-1} (~ 16.7 µM)[c]	- (72 h)	[62]
	HeLa	in vitro	IC_{50}[b] = 70.1 µgmL^{-1} (~ 21.1 µM)[c]	- (72 h)	[62]
$K_5[(NC_3H_4Sn)_2PW_{10}O_{38}]$	SSMC-7721	in vitro	IC_{50}[b] = 34.2 µgmL^{-1} (~ 10.4 µM)[c]	- (72 h)	[62]
	HeLa	in vitro	IC_{50}[b] = 50.8 µgmL^{-1} (~ 15.5 µM)[c]	- (72 h)	[62]
α-$K_4H_3[(C_4H_7O_2Sn)_3SiW_9O_{37}]$	SSMC-7721	in vitro	IC_{50}[b] = 90.9 µgmL^{-1} (~ 28.1 µM)[c]	- (72 h)	[63]
	HeLa	in vitro	IC_{50}[b] = 93.2 µgmL^{-1} (~ 28.8 µM)[c]	- (72 h)	[63]
β-$K_4H_3[(C_4H_7O_2Sn)_3SiW_9O_{37}]$	SSMC-7721	in vitro	IC_{50}[b] = 61.7 µgmL^{-1} (~ 19.1 µM)[c]	- (72 h)	[63]
	HeLa	in vitro	IC_{50}[b] = 80.6 µgmL^{-1} (~ 24.9 µM)[c]	- (72 h)	[63]
α-$K_4H_3[(C_5H_9O_2Sn)_3SiW_9O_{37}]$	SSMC-7721	in vitro	IC_{50}[b] = 90.0 µgmL^{-1} (~ 27.5 µM)[c]	- (72 h)	[63]
	HeLa	in vitro	IC_{50}[b] = 93.0 µgmL^{-1} (~ 28.4 µM)[c]	- (72 h)	[63]
β-$K_4H_3[(C_5H_9O_2Sn)_3SiW_9O_{37}]$	SSMC-7721	in vitro	IC_{50}[b] = 61.1 µgmL^{-1} (~ 18.7 µM)[c]	- (72 h)	[63]
	HeLa	in vitro	IC_{50}[b] = 79.3 µgmL^{-1} (~ 24.2 µM)[c]	- (72 h)	[63]
α-$K_4H_3[(NC_3H_4Sn)_3SiW_9O_{37}]$	SSMC-7721	in vitro	IC_{50}[b] = 43.2 µgmL^{-1} (~ 13.8 µM)[c]	- (72 h)	[63]
	HeLa	in vitro	IC_{50}[b] = 76.2 µgmL^{-1} (~ 24.3 µM)[c]	- (72 h)	[63]
β-$K_4H_3[(NC_3H_4Sn)_3SiW_9O_{37}]$	SSMC-7721	in vitro	IC_{50}[b] = 29.9 µgmL^{-1} (~ 9.5 µM)[c]	- (72 h)	[63]

	HeLa	in vitro	IC_{50}[b] = 68.3 µgmL^{-1} (~ 21.7 µM)[c]	- (72 h)	[63]
α-$K_7H_4[(C_4H_7O_2Sn)_3(SiW_9O_{34})_2]$	SSMC-7721	in vitro	IC_{50}[b] = 60.7 µgmL^{-1} (~ 10.8 µM)[c]	- (72 h)	[63]
	HeLa	in vitro	IC_{50}[b] = 87.6 µgmL^{-1} (~ 15.6 µM)[c]	- (72 h)	[63]
β-$K_7H_4[(C_4H_7O_2Sn)_3(SiW_9O_{34})_2]$	SSMC-7721	in vitro	IC_{50}[b] = 52.1 µgmL^{-1} (~ 9.4 µM)[c]	- (72 h)	[63]
	HeLa	in vitro	IC_{50}[b] = 77.5 µgmL^{-1} (~ 14.0 µM)[c]	- (72 h)	[63]
α-$K_7H_4[(NC_3H_4Sn)_3(SiW_9O_{34})_2]$	SSMC-7721	in vitro	IC_{50}[b] = 28.7 µgmL^{-1} (~ 5.2 µM)[c]	- (72 h)	[63]
	HeLa	in vitro	IC_{50}[b] = 61.1 µgmL^{-1} (~ 11.1 µM)[c]	- (72 h)	[63]
β-$K_7H_4[(NC_3H_4Sn)_3(SiW_9O_{34})_2]$	SSMC-7721	in vitro	IC_{50}[b] = 19.8 µgmL^{-1} (~ 3.6 µM)[c]	- (72 h)	[63]
	HeLa	in vitro	IC_{50}[b] = 54.9 µgmL^{-1} (~ 10.0 µM)[c]	- (72 h)	[63]
$K_4H_5[(C_4H_7O_2Sn)_3(PW_9O_{34})_2]$	SSMC-7721	in vitro	IC_{50}[b] = 47.6 µgmL^{-1} (~ 8.7 µM)[c]	- (72 h)	[60]
	HeLa	in vitro	IC_{50}[b] = 49.9 µgmL^{-1} (~ 9.1 µM)[c]	- (72 h)	[60]
$K_4H_5[(C_5H_9O_2Sn)_3(PW_9O_{34})_2]$	SSMC-7721	in vitro	IC_{50}[b] = 40.3 µgmL^{-1} (~ 7.3 µM)[c]	- (72 h)	[60]
	HeLa	in vitro	IC_{50}[b] = 45.7 µgmL^{-1} (~ 8.3 µM)[c]	- (72 h)	[60]
α-$K_4H_3[(C_4H_7O_2Sn)_3GeW_9O_{37}]$	SSMC-7721	in vitro	IC_{50}[b] = 122.6 µgmL^{-1} (~ 37.4 µM)[c]	- (72 h)	[59]
	HeLa	in vitro	IC_{50}[b] = 163.6 µgmL^{-1} (~ 49.9 µM)[c]	- (72 h)	[59]
β-$K_4H_3[(C_4H_7O_2Sn)_3GeW_9O_{37}]$	SSMC-7721	in vitro	IC_{50}[b] = 97.7 µgmL^{-1} (~ 29.7 µM)[c]	- (72 h)	[59]
	HeLa	in vitro	IC_{50}[b] = 100.4 µgmL^{-1} (~ 30.5 µM)[c]	- (72 h)	[59]
$K_4H_3[(C_4H_7O_2Sn)P_2W_{17}O_{61}]$	SSMC-7721	in vitro	IC_{50}[b] = 86.3 µgmL^{-1} (~ 18.3 µM)[c]	- (72 h)	[60]
	HeLa	in vitro	IC_{50}[b] = 90.2 µgmL^{-1} (~ 19.1 µM)[c]	- (72 h)	[60]
$K_4H_3[(C_5H_9O_2Sn)P_2W_{17}O_{61}]$	SSMC-7721	in vitro	IC_{50}[b] = 79.1 µgmL^{-1} (~ 16.7 µM)[c]	- (72 h)	[60]

	HeLa	in vitro	IC_{50}[b] = 86.7 μgmL^{-1} (~ 18.3 µM)[c]	- (72 h)	[60]
$(TBA)_4[(\eta^5\text{-}C_5H_5V)PW_{11}O_{39}]$	HL-60	in vitro	IE[f] = 42.2 %	51.1 μgmL^{-1} (n.d.)	[64]
	B16	in vitro	IE[f] = 26.1 %	51.1 μgmL^{-1} (n.d.)	[64]
$((CH_3)_4N)_3H_2[(\eta^5\text{-}C_5H_5Ti)GeW_{11}O_{39}]$	SSMC-7721	in vitro	IC_{50}[b] = 47.8 μgmL^{-1} (~ 15.7 µM)[c]	- (72 h)	[65]
	HeLa	in vitro	IC_{50}[b] = 54.4 μgmL^{-1} (~ 17.8 µM)[c]	- (72 h)	[65]
$((CH_3)_4N)_3H_2[(\eta^5\text{-}C_5H_5Ti)GaW_{11}O_{39}]$	SSMC-7721	in vitro	IC_{50}[b] = 42.6 μgmL^{-1} (~ 14.0 µM)[c]	- (72 h)	[65]
	HeLa	in vitro	IC_{50}[b] = 48.3 μgmL^{-1} (~ 15.8 µM)[c]	- (72 h)	[65]
$((CH_3)_4N)_3H_2[(\eta^5\text{-}C_5H_5Ti)BW_{11}O_{39}]$	SSMC-7721	in vitro	IC_{50}[b] = 38.9 μgmL^{-1} (~ 12.9 µM)[c]	- (72 h)	[65]
	HeLa	in vitro	IC_{50}[b] = 43.6 μgmL^{-1} (~ 14.6 µM)[c]	- (72 h)	[65]
$K_6H[(\eta^5\text{-}C_5H_5Ti)CoW_{11}O_{39}]$	**HL-60**	**in vivo**	**TWI[d] = 50.0 %**	**100.0 $mgkg^{-1}$ (11 d)**	**[31]**
	**HLC**	**in vivo**	**TWI[d] = 48.9 %**	**100.0 $mgkg^{-1}$ (11 d)**	**[31]**
	**SSMC-7721**	**in vivo**	**TWI[d] = 41.9 %**	**15.0 $mgkg^{-1}$ (11 d)**	**[31]**
	SSMC-7721	in vitro	IC_{50}[b] = 3.2 µM	- (72 h)	**[31]**
	HeLa	in vitro	IC_{50}[b] = 11.5 µM	- (72 h)	[31]
	MCF-7	in vitro	IC_{50}[b] = 9.3 µM	- (24 h)	[66]
	HEK-293	in vitro	IC_{50}[b] = 11.8 µM	- (24 h)	[66]
	A-549	in vitro	IC_{50}[b] = 900.0 µM	- (24 h)	[67]
	C2C12[d]	in vitro	IC_{50}[b] = 4800.0 µM	- (24 h)	[67]
$K_6H[(\eta^5\text{-}C_5H_5Zr)CoW_{11}O_{39}]$	MCF-7	in vitro	IC_{50}[b] = 15.4 µM	- (24 h)	[66]
	HEK-293	in vitro	IC_{50}[b] = 15.4 µM	- (24 h)	[66]
$K_7H_2[(\eta^5\text{-}C_5H_5Fe)CoW_{11}O_{39}]$	MCF-7	in vitro	IC_{50}[b] = 6.3 µM	- (24 h)	[66]
	HEK-293	in vitro	IC_{50}[b] = 8.8 µM	- (24 h)	[66]
$\gamma\text{-}(TBA)_6[(\eta^5\text{-}C_5H_5Ti)_2SiW_{10}O_{38}]$	SSMC-7721	in vitro	IC_{50}[b] = 13.2 μgmL^{-1} (~ 4.4 µM)[c]	- (72 h)	[61]
	HeLa	in vitro	IC_{50}[b] = 26.4 μgmL^{-1} (~ 8.8 µM)[c]	- (72 h)	[61]
$\gamma\text{-}(TBA)_6[(\eta^5\text{-}C_5H_5Zr)_2SiW_{10}O_{38}]$	SSMC-7721	in vitro	IC_{50}[b] = 40.6 μgmL^{-1} (~ 13.2 µM)[c]	- (72 h)	[61]
	HeLa	in vitro	IC_{50}[b] = 63.3 μgmL^{-1} (~ 20.5 µM)[c]	- (72 h)	[61]
$\alpha\text{-}K_4H_3[(\eta^5\text{-}C_5H_5Ti)_3SiW_9O_{37}]$	SSMC-7721	in vitro	IC_{50}[b] = 21.6 μgmL^{-1} (~ 7.8 µM)[c]	- (72 h)	[68]
	HeLa	in vitro	IC_{50}[b] = 38.7 μgmL^{-1}	- (72 h)	[68]

			(~ 14.0 μM)[c]		
β-$K_4H_3[(\eta^5\text{-}C_5H_5Ti)_3SiW_9O_{37}]$	SSMC-7721	in vitro	IC_{50}[b] = 18.6 μgmL^{-1} (~ 6.7 μM)[c]	- (72 h)	[68]
	HeLa	in vitro	IC_{50}[b] = 33.6 μgmL^{-1} (~ 12.1 μM)[c]	- (72 h)	[68]
α-$K_4H_3[(\eta^5\text{-}C_5H_5Ti)_3GeW_9O_{37}]$	SSMC-7721	in vitro	IC_{50}[b] = 11.2 μgmL^{-1} (~ 4.0 μM)[c]	- (72 h)	[68]
	HeLa	in vitro	IC_{50}[b] = 21.3 μgmL^{-1} (~ 7.6 μM)[c]	- (72 h)	[68]
β-$K_4H_3[(\eta^5\text{-}C_5H_5Ti)_3GeW_9O_{37}]$	SSMC-7721	in vitro	IC_{50}[b] = 23.7 μgmL^{-1} (~ 8.4 μM)[c]	- (72 h)	[68]
	HeLa	in vitro	IC_{50}[b] = 25.6 μgmL^{-1} (~ 9.1 μM)[c]	- (72 h)	[68]
$K_7H_2[(\eta^5\text{-}C_5H_5Ti)_3P_2W_{15}O_{59}]$	SSMC-7721	in vitro	IC_{50}[b] = 20.8 μgmL^{-1} (~ 4.7 μM)[c]	- (72 h)	[69]
	HeLa	in vitro	IC_{50}[b] = 47.2 μgmL^{-1} (~ 10.6 μM)[c]	- (72 h)	[69]
$(TBA)_9[(\eta^5\text{-}C_5H_5Zr)_3P_2W_{15}O_{59}]$	SSMC-7721	in vitro	IC_{50}[b] = 40.6 μgmL^{-1} (~ 6.3 μM)[c]	- (72 h)	[69]
	HeLa	in vitro	IC_{50}[b] = 63.3 μgmL^{-1} (~ 9.9 μM)[c]	- (72 h)	[69]
$K_4H_5[(C_4H_7O_2Sn)_3P_2W_{15}O_{59}]$	SSMC-7721	in vitro	IC_{50}[b] = 30.6 μgmL^{-1} (~ 6.5 μM)[c]	- (72 h)	[69]
	HeLa	in vitro	IC_{50}[b] = 50.1 μgmL^{-1} (~ 10.6 μM)[c]	- (72 h)	[69]
$K_4H_5[(C_5H_9O_2Sn)_3P_2W_{15}O_{59}]$	SSMC-7721	in vitro	IC_{50}[b] = 30.1 μgmL^{-1} (~ 6.3 μM)[c]	- (72 h)	[69]
	HeLa	in vitro	IC_{50}[b] = 48.3 μgmL^{-1} (~ 10.1 μM)[c]	- (72 h)	[69]
$K_3H[\{(n\text{-}Bu)Sn(OH)\}_3GeW_9O_{34}]$	**H22**	**in vivo**	**TWI[d] = 62.5 %**	**300.0 $mgkg^{-1}$ (14 d)**	**[70]**
	H22	in vitro	IE[e] ~ 90.0 %[f]	40.0 μgmL^{-1} (72 h)	[70]
	SW-620	in vitro	IE[e] ~ 90.0 %[f]	40.0 μgmL^{-1} (72 h)	[70]
	MGC-803	in vitro	IE[e] ~ 50.0 %[f]	40.0 μgmL^{-1} (72 h)	[70]
	A-549	in vitro	IE[e] ~ 85.0 %[f]	40.0 μgmL^{-1} (72 h)	[70]
	MM-231	in vitro	IE[e] ~ 77.0 %[f]	40.0 μgmL^{-1} (72 h)	[70]
	Hep-G2	in vitro	IC_{50}[b] = 19.2 μgmL^{-1} (~ 5.6 μM)[c]	- (72 h)	[70]
	L-02[d]	in vitro	IC_{50}[b] = 42.8 μgmL^{-1} (~ 12.5 μM)[c]	- (72 h)	[70]
$((CH_3)_4N)_5[(C_3H_5O_2Ge)SiW_{11}O_{39}]$	S180	in vitro	IE[e] = 97.2 %	53.3 μgmL^{-1} (n.d.)	[34]
	leucocythemia	in vitro	IE[e] = 88.9 %	53.3 μgmL^{-1} (n.d.)	[34]

$((CH_3)_4N)_5[(C_3H_4O_2(C_6H_4N\text{-}3\text{-}NO_2)Ge)SiW_{11}O_{39}]$	S180	in vitro	IE[e] = 96.6 %	51.6 µgmL^{-1} (n.d.)	[34]
	leucocythemia	in vitro	IE[e] = 83.1 %	51.6 µgmL^{-1} (n.d.)	[34]
$((CH_3)_4N)_4[(C_3H_5O_2Ge)PW_{11}O_{39}]$	S180	in vitro	IE[e] = 97.2 %	53.3 µgmL^{-1} (n.d.)	[34]
	leucocythemia	in vitro	IE[e] = 88.9 %	53.3 µgmL^{-1} (n.d.)	[34]
$((CH_3)_4N)_4[(C_3H_4O_2(C_6H_4N\text{-}3\text{-}NO_2)Ge)PW_{11}O_{39}]$	S180	in vitro	IE[e] = 87.4 %	51.5 µgmL^{-1} (n.d.)	[34]
	leucocythemia	in vitro	IE[e] = 98.5 %	51.5 µgmL^{-1} (n.d.)	[34]
$((CH_3)_4N)_4[(C_3H_5O_2Ge)GeW_{11}O_{39}]$	S180	in vitro	IE[e] = 88.2 %	55.5 µgmL^{-1} (n.d.)	[34]
	leucocythemia	in vitro	IE[e] = 96.7 %	55.5 µgmL^{-1} (n.d.)	[34]
$((CH_3)_4N)_4[(C_3H_4O_2(C_6H_4\text{-}3\text{-}NO_2)Ge)GeW_{11}O_{39}]$	S180	in vitro	IE[e] = 88.1 %	54.0 µgmL^{-1} (n.d.)	[34]
	leucocythemia	in vitro	IE[e] = 92.6 %	54.0 µgmL^{-1} (n.d.)	[34]
$Na_{15}[(Mn(COOH))_3(AsW_9O_{33})_2]$	HeLa	in vitro	IC_{50}[e] = 8.9 µgmL^{-1} (~ 1.6 µM)[c]	- (48 h)	[38]

[a] the value in parentheses indicates at which time (after addition of POM) the antitumor effect was measured, that is, in in vivo studies it is the time at which the tumor size was measured (d = days) and in in vitro studies it is the time at which the inhibitory effect was determined (h = hours). [b] IC_{50} = the dose required to inhibit or kill 50% of the tested cells. [c] these values were converted from µgmL^{-1} units (original reference) into µM units by us or by reference [71] for comparison reasons. [d] TWI = % tumor weight inhibition (in comparison to the control). [e] IE = inhibitory effect describing the % inhibition of cells in in vitro studies. [f] only the graph plotting the antitumor activity was provided by the authors and therefore the values were read from the graph. [g] these cells are normal noncancerous cells to validate toxicity. $(CH_3)_4N$ = tetramethylammonium, TBA = tetra-*n*-butyl ammonium, *n*-Bu = *n*-butyl, n.d. = not defined. **In vivo results are highlighted in green**.

Table S5. Anticancer activity of POM-drug, POM-rare earth metal, POM-5-FU-rare earth metal complexes and 5-FU.

POM-drug hybrid	Cell line	Exp.	Activity	Dose (time)[a]	Ref.
$(C_4H_4FN_2O_2H_3)[SiW_{12}O_{40}]$	Hep-G2	in vitro	IC_{50}[b] = 59.1 µM	- (n.d.)	[72]
	HEK-293[c]	in vitro	IC_{50}[b] = 28.8 µM	- (n.d.)	[72,73]
$(C_4H_4FN_2O_2H_2)[PW_{12}O_{40}]$	HeLa	in vitro	IC_{50}[b] = 7.3 µM	- (72 h)	[40]
	Hep-G2	in vitro	IC_{50}[b] = 8.3 µM	- (72 h)	[40]
	HEK-293[c]	in vitro	IC_{50}[b] = 13.9 µM	- (72 h)	[40]
$(C_4H_4FN_2O_2H_4)[BW_{12}O_{40}]$	SMMC-7721	in vitro	IC_{50}[b] = 1.3 µM	- (n.d.)	[29]
$K_{26}[(C_4H_4FN_2O_2)_8Nd(SiW_{11}O_{39})_4]$	Hep-G2	in vitro	IC_{50}[b] = 11.0. µM	- (n.d.)	[72]
	HEK-293[c]	in vitro	IC_{50}[b] = 45.9 µM	- (n.d.)	[72]
$K_{26}[(C_4H_4FN_2O_2)_8Pr(SiW_{11}O_{39})_4]$	Hep-G2	in vitro	IC_{50}[b] = 19.4 µM	- (72 h)	[74]
	HEK-293[c]	in vitro	IC_{50}[b] = 14.7 µM[d]	- (72 h)	[74]
$K_{26}[(C_4H_4FN_2O_2)_8Sm(SiW_{11}O_{39})_4]$	Hep-G2	in vitro	IC_{50}[b] = 13.2 µM	- (72 h)	[74]
	HEK-293[c]	in vitro	IC_{50}[b] = 20.9 µM[d]	- (72 h)	[74]
$K_{10}[(C_4H_4FN_2O_2)Y(PW_{11}O_{39})_2]$	HeLa	in vitro	IC_{50}[b] = 5.1 µM	- (72 h)	[40]
	Hep-G2	in vitro	IC_{50}[b] = 3.1 µM	- (72 h)	[40]
	HEK-293[c]	in vitro	IC_{50}[b] = 10.4 µM	- (72 h)	[40]
$K_{11}[Y(PW_{11}O_{39})_2]$	HeLa	in vitro	IC_{50}[b] = 128.0 µM	- (72 h)	[40]
	Hep-G2	in vitro	IC_{50}[b] = 55.5 µM	- (72 h)	[40]
	HEK-293[c]	in vitro	IC_{50}[b] = 71.3 µM	- (72 h)	[40]
$K_9[(C_4H_4FN_2O_2)_2La(PW_{11}O_{39})_2]$	HeLa	in vitro	IC_{50}[b] = 4.2 µM	- (72 h)	[40]
	Hep-G2	in vitro	IC_{50}[b] = 7.2 µM	- (72 h)	[40]
	HEK-293[c]	in vitro	IC_{50}[b] = 12.9 µM	- (72 h)	[40]
$K_{11}[La(PW_{11}O_{39})_2]$	HeLa	in vitro	IC_{50}[b] = 378.0 µM	- (72 h)	[40]
	Hep-G2	in vitro	IC_{50}[b] = 62.1 µM	- (72 h)	[40]
	HEK-293[c]	in vitro	IC_{50}[b] = 74.6 µM	- (72 h)	[40]
$K_9[(C_4H_4FN_2O_2)_2Er(PW_{11}O_{39})_2]$	HeLa	in vitro	IC_{50}[b] = 6.0 µM	- (72 h)	[75]
	Hep-G2	in vitro	IC_{50}[b] = 6.9 µM	- (72 h)	[75]
	HEK-293[c]	in vitro	IC_{50}[b] = 10.8 µM	- (72 h)	[75]
$K_{11}[Er(PW_{11}O_{39})_2]$	HeLa	in vitro	IC_{50}[b] = 382.0 µM	- (72 h)	[75]
	Hep-G2	in vitro	IC_{50}[b] = 56.2 µM	- (72 h)	[75]
	HEK-293[c]	in vitro	IC_{50}[b] = 78.5 µM	- (72 h)	[75]
$K_{10}[(C_4H_4FN_2O_2)Gd(PW_{11}O_{39})_2]$	HeLa	in vitro	IC_{50}[b] = 7.1 µM	- (72 h)	[75]
	Hep-G2	in vitro	IC_{50}[b] = 5.6 µM	- (72 h)	[75]
	HEK-293[c]	in vitro	IC_{50}[b] = 10.3 µM	- (72 h)	[75]
$K_{11}[Gd(PW_{11}O_{39})_2]$	HeLa	in vitro	IC_{50}[b] = 118.0 µM	- (72 h)	[75]
	Hep-G2	in vitro	IC_{50}[b] = 51.4 µM	- (72 h)	[75]
	HEK-293[c]	in vitro	IC_{50}[b] = 27.6 µM	- (72 h)	[75]

$K_{10}[(C_4H_4FN_2O_2)Dy(PW_{11}O_{39})_2]$	HeLa	in vitro	IC_{50}[b] = 6.2 µM	- (72 h)	[75]
	Hep-G2	in vitro	IC_{50}[b] = 5.7 µM	- (72 h)	[75]
	HEK-293[c]	in vitro	IC_{50}[b] = 10.8 µM	- (72 h)	[75]
$K_{11}[Dy(PW_{11}O_{39})_2]$	HeLa	in vitro	IC_{50}[b] = 111.0 µM	- (72 h)	[75]
	Hep-G2	in vitro	IC_{50}[b] = 44.7 µM	- (72 h)	[75]
	HEK-293[g]	in vitro	IC_{50}[b] = 19.3 µM	- (72 h)	[75]
$K_9[(C_4H_4FN_2O_2)_2Sm(PW_{11}O_{39})_2]$	HeLa	in vitro	IC_{50}[b] = 4.2 µM	- (72 h)	[76]
	Hep-G2	in vitro	IC_{50}[b] = 4.6 µM	- (72 h)	[76]
	HEK-293[c]	in vitro	IC_{50}[b] = 9.1 µM	- (72 h)	[76]
$K_9H[(C_4H_4FN_2O_2)Eu(PW_{11}O_{39})_2]$	HeLa	in vitro	IC_{50}[b] = 3.5 µM	- (72 h)	[76]
	Hep-G2	in vitro	IC_{50}[b] = 7.2 µM	- (72 h)	[76]
	HEK-293[c]	in vitro	IC_{50}[b] = 17.9 µM	- (72 h)	[76]
5-FU ($C_4H_4FN_2O_2$)	HeLa	in vitro	IC_{50}[b] = 10.8 µM	- (72 h)	[40]
	Hep-G2	in vitro	IC_{50}[b] = 9.1 µM	- (72 h)	[40]
	SSMC-7721	in vitro	IC_{50}[b] = 24.1 µM	- (n.d.)	[29]
	HEK-293[c]	in vitro	IC_{50}[b] = 13.2 µM	- (72 h)	[40]
$(C_6H_9N_2O_2S)_5HP_2Mo_{18}O_{62}$	PC-3m	in vitro	IC_{50}[b] = 38.0 µgmL^{-1} (~ 9.7 µM)*	- (72 h)	[77]
$(C_6H_9N_2O_2S)H_8P_2V_3Mo_{15}O_{62}$	PC-3m	in vitro	IC_{50}[b] = 11.0 µgmL^{-1} (~ 3.7 µM)*	- (72 h)	[77]

[a] the value in parentheses indicates at which time (after addition of POM) the antitumor effect was measured, that is, in in vivo studies it is the time at which the tumor size was measured (d = days) and in in vitro studies it is the time at which the inhibitory effect was determined (h = hours). [b] IC_{50} = the dose required to inhibit or kill 50% of the tested cells. [c] these cells are normal noncancerous cells to validate toxicity. [d] these values were calculated by us based on the IC_{50} value and the therapeutic index of the compound. * the values in parentheses indicate the IC_{50} value in µM unit, note that this is a rough estimation as in most cases the water content of the structure was not provided by the authors and therefore the molecular weight of the respective POM could not be defined accurately. n.d. = not defined.

Table S6. Anticancer activity of POM-bisphosphonate complexes.

POM-drug hybrid	Cell line	Exp.	Activity	Dose (time)[a]	Ref.
$Li_8[(Mo^V{}_2O_4(H_2O))_4(O_3PC(C_3H_6NH_3)OPO_3)_4]$	NCI-H460	in vitro	IC_{50}[b] = 109.0 µM	- (96 h)	[8]
= $Li_8[Mo^V{}_8Ale_4]$	MCF-7	in vitro	IC_{50}[b] = 107.0 µM	- (96 h)	[8]
	SF-268	in vitro	IC_{50}[b] = 365.0 µM	- (96 h)	[8]
$[(C_2H_5)_2NH_2]_4[Mo^V{}_4O_8(O_3PC(C_3H_6NH_3)OPO_3)_2]$	NCI-H460	in vitro	IC_{50}[b] = 201.0 µM	- (96 h)	[8]
= $[(C_2H_5)_2NH_2]_4[Mo^V{}_6Ale_2]$	MCF-7	in vitro	IC_{50}[b] = 171.0 µM	- (96 h)	[8]
	SF-268	in vitro	IC_{50}[b] = 105.0 µM	- (96 h)	[8]
$[(C_2H_5)_2NH_2]_6[Mo^V{}_4O_8(O_3PC(C_{10}H_{14}NO)OPO_3)_2]$	NCI-H460	in vitro	IC_{50}[b] = 443.0 µM	- (96 h)	[8]
= $[(C_2H_5)_2NH_2]_6[Mo^V{}_4Ale_2]$	MCF-7	in vitro	IC_{50}[b] = 126.0 µM	- (96 h)	[8]
	SF-268	in vitro	IC_{50}[b] = 39.0 µM	- (96 h)	[8]
$Na_2Rb_6[(Mo_3O_8)_4(O_3PC(C_3H_6NH_3)OPO_3)_4]$	NCI-H460	in vitro	IC_{50}[b] = 9.2 - 12.0 µM	- (96 h)	[8,55]
= $Na_2Rb_6[Mo_{12}Ale_4]$	MCF-7	in vitro	IC_{50}[b] = 5.6 - 11.0 µM	- (96 h)	[8,55]
	SF-268	in vitro	IC_{50}[b] = 5.0 - 7.0 µM	- (96 h)	[8,55]
$Na_3\{Na(Mo_3O_8)_4[O_3PCC_3H_6NH_2CH_2(C_5H_5N)OPO_3]_4\}$	MCF-7	in vitro	IC_{50}[b] = 54.0 µM	- (96 h)	[78]
= $Na_3[Mo_{12}(AlePy)_4]$					
$Na_8\{Pt_4Cl_8(Mo_3O_8)_4[O_3PCC_3H_6NH_2CH_2(C_5H_4N)OPO_3]_4\}$	MCF-7	in vitro	IC_{50}[b] = 100.0 µM	- (96 h)	[78]
= $Na_8[Mo_{12}(AlePy)_3Pt_4]$					
$Rb_{0.25}(NH_4)_{5.75}[(Mo_3O_8)_2O(O_3PC(C_3H_6NH_3)OPO_3)_2]$	NCI-H460	in vitro	IC_{50}[b] = 48.0 µM	- (96 h)	[55]
= $Rb_{0.25}(NH_4)_{5.75}[Mo_6Ale_2]$	MCF-7	in vitro	IC_{50}[b] = 56.0 µM	- (96 h)	[55]
	SF-268	in vitro	IC_{50}[b] = 130. µM	- (96 h)	[55]
$(NH_4)_6[(Mo_3O_8)_2O(O_3PC(C_4H_6N_2)OPO_3)_2]$	NCI-H460	in vitro	IC_{50}[b] = 2.4 - 10.0 µM	- (96 h)	[55,79]
= $(NH_4)_6[Mo_6Zol_2]$	MCF-7	in vitro	IC_{50}[b] = 2.2 µM	- (96 h)	[55]
	SF-268	in vitro	IC_{50}[b] = 3.0 µM	- (96 h)	[55]
$(NH_4)_6[(Mo_3O_8)_2O(O_3PC(C_4H_5N_2)(C_6H_{13})OPO_3)_2]$	NCI-H460	in vitro	IC_{50}[b] = 3.5 µM	- (96 h)	[79]
= $(NH_4)_6[Mo_6(ZolC_6)_2]$					
$(NH_4)_6[(Mo_3O_8)_2O(O_3PC(C_4H_5N_2)(C_8H_{17})OPO_3)_2]$	NCI-H460	in vitro	IC_{50}[b] = 1.9 µM	- (96 h)	[79]
= $(NH_4)_6[Mo_6(ZolC_8)_2]$					
$Rb_{0.25}(NH_4)_{5.75}[(Mo_3O_8)_2O(O_3PC(CH_2S(CH_3)_2)OPO_3)_2]$	NCI-H460	in vitro	IC_{50}[b] = 46.0 µM	- (96 h)	[55]
= $Rb_{0.25}(NH_4)_{5.75}[Mo_6Sul_2]$	MCF-7	in vitro	IC_{50}[b] = 35.0 µM	- (96 h)	[55]
	SF-268	in vitro	IC_{50}[b] = 100.0 µM	- (96 h)	[55]
$(NH_4)_{5.5}Na_{0.5}[(Mo_2O_6)_2(O_3PC(C_3H_6NH_3)OPO_3)_2Mn^{II}]$	MCF-7	in vitro	IC_{50}[b] = 5.7 µM	- (96 h)	[78]
= $(NH_4)_{5.5}Na_{0.5}[Mo_4Ale_2Mn^{II}]$					
$(NH_4)_5[(Mo_2O_6)_2(O_3PC(C_3H_6NH_3)OPO_3)_2Fe^{III}]$	MCF-7	in vitro	IC_{50}[b] = 55.0 µM	- (96 h)	[78]
= $(NH_4)_5[Mo_4Ale_2Fe^{III}]$					
$(NH_4)_5[(Mo^{VI}{}_2O_6)_2(O_3PC(C_4H_6N_2)OPO_3)_2Mn^{III}]$	**SK-ES-1**	**in vivo**	**TWI[d] = ~ 85.0 %[e]**	**5 µg/body (28 d)**	**[79]**
= $Rb(NH_4)_4[Mo_4Zol_2Mn^{III}]$	NCI-H460	in vitro	IC_{50}[b] = 2.6 µM	- (96 h)	[79]
	MCF-7	in vitro	IC_{50}[b] = 1.3 µM	- (96 h)	[78]

$(NH_4)_5(H_3O)[(Mo^{VI}_2O_6)_2(O_3PC(C_4H_6N_2)OPO_3)_2Mn^{II}]$<br>= $(NH_4)_5(H_3O)[(Mo_4Zol_2Mn^{II}]$	NCI-H460	in vitro	IC_{50}[b] = 2.0 µM	- (96 h)	[79]
$(NH_4)_5[(Mo_2O_6)_2(O_3PC(CH_2C_3H_4N_2)OPO_3)_2Fe^{III}]$<br>= $(NH_4)_5[(Mo_4Zol_2Fe^{III}]$	MCF-7	in vitro	IC_{50}[b] = 6.6 µM	- (96 h)	[78]
$Li_2(NH_4)_3[(Mo^{VI}_2O_6)_2(O_3PC(C_4H_5N_2)(C_8H_{17})OPO_3)_2Mn^{III}]$<br>= $Li_2(NH_4)_3[Mo_4(ZolC_8)_2Mn^{III}]$	NCI-H460	in vitro	IC_{50}[b] = 2.9 µM	- (96 h)	[79]
$(NH_4)_5[(Mo_2O_6)_2(O_3PC(C_7H_{14}NH_3)OPO_3)_2Fe^{III}]$<br>= $(NH_4)_5[Mo_4(BPC_8NH_2)_2Fe^{III}]$	MCF-7	in vitro	IC_{50}[b] = 200.0 µM	- (96 h)	[78]
$Na_{0.5}(NH_4)_{6.5}[(Mo_2O_6)_2(O_3PC(C_8H_{17})OPO_3)_2Fe^{III}]$<br>= $Na_{0.5}(NH_4)_{6.5}[Mo_4(BPC_9)_2Fe^{III}]$	MCF-7	in vitro	IC_{50}[b] = 87.0 µM	- (96 h)	[78]
$Na_6Rb_2[(V_6O_{10}(H_2O)_2(O_3PC(C_3H_6NH_3)OPO_3)_4]$	NCI-H460	in vitro	IC_{50}[b] = 0.4 µM	- (96 h)	[55]
= $Na_6Rb_2[V_6Ale_4]$	MCF-7	in vitro	IC_{50}[b] = 0.5 µM	- (96 h)	[55]
	SF-268	in vitro	IC_{50}[b] = 0.5 µM	- (96 h)	[55]
$Na_3[V_3O_3(H_2O)(O_3PC(C_4H_6N_2)(OH)PO_3)_3]$	NCI-H460	in vitro	IC_{50}[b] = 0.3 µM	- (96 h)	[55]
= $Na_3[V_3Zol_3]$	MCF-7	in vitro	IC_{50}[b] = 0.3 µM	- (96 h)	[55]
	SF-268	in vitro	IC_{50}[b] = 0.3 µM	- (96 h)	[55]
$(NH_4)_2Rb_2[(V_5O_9(OH)_2(H_2O)(O_3PC(C_3H_6NH_3)OPO_3)_2]$	NCI-H460	in vitro	IC_{50}[b] = 0.5 µM	- (96 h)	[55]
= $(NH_4)_2Rb_2[V_5Ale_2]$	MCF-7	in vitro	IC_{50}[b] = 0.5 µM	- (96 h)	[55]
	SF-268	in vitro	IC_{50}[b] = 0.8 µM	- (96 h)	[55]
$(NH_4)_{2.5}K_{1.5}[(V_5O_9(OH)_2(H_2O)(O_3PC(C_4H_6N_2)OPO_3)_2]$	NCI-H460	in vitro	IC_{50}[b] = 0.5 µM	- (96 h)	[55]
= $(NH_4)_{2.5}K_{1.5}[V_5Zol_2]$	MCF-7	in vitro	IC_{50}[b] = 0.4 µM	- (96 h)	[55]
	SF-268	in vitro	IC_{50}[b] = 0.4 µM	- (96 h)	[55]

[a] the value in parentheses indicates at which time (after addition of POM) the antitumor effect was measured, that is, in in vivo studies it is the time at which the tumor size was measured (d = days) and in in vitro studies it is the time at which the inhibitory effect was determined (h = hours). [b] IC_{50} = the dose required to inhibit or kill 50% of the tested cells. [c] these cells are normal noncancerous cells to validate toxicity. [d] TWI = % tumor weight inhibition (in comparison to the control). [e] only the graph plotting the antitumor activity was provided by the authors and therefore the values were read from the graph. Ale = alendronate, AlePy = 2-pyridyl alendronate, BPC_9 = n-alkyl bisphosphonate, BPC_8NH_2 = aminoalkyl bisphosphonate, Sul = (2-Hydroxy-2,2-bis-phosphono-ethyl)-dimethyl-sulfonium, Zol = zoledronate, $ZolC_6$ = zoledronate with attached C_6 alkyl chain, $ZolC_8$ = zoledronate with attached C_8 alkyl chain. **In vivo results are highlighted in green**.

Table S7. Anticancer activity of POM-quinolone antibiotic complexes.

POM-drug hybrid	Cell line	Exp.	Activity	Dose (time)[a]	Ref.
$\{[Co(PPA)_2]H_2[SiW_{12}O_{40}]\}$	MCF-7	in vitro	IC_{50}[b] = 92.0 µgmL^{-1} (~ 23.6 µM)*	- (n.d.)	[80]
$\{[Ni(PPA)_2]H_4[SiW_{12}O_{40}]\}$	MCF-7	in vitro	no activity	100.0 µgmL^{-1} (48 h)	[81]
$\{[Zn(PPA)_2]_2H_4[SiW_{12}O_{40}]\}$	MCF-7	in vitro	IC_{50}[b] = 19.0 µgmL^{-1} (~ 4.4 µM)*	- (48 h)	[81]
$\{[Cu_2(Enro)_3H_2O][SiW_{12}O_{40}]\}_2$	SGC-7901	in vitro	no activity	100.0 µgmL^{-1} (72 h)	[82]
	SMMC-7721	in vitro	no activity	100.0 µgmL^{-1} (72 h)	[82]
$\{[Cu(Norf)_2]\}_2[SiW_{12}O_{40}]$	SGC-7901	in vitro	no activity	100.0 µgmL^{-1} (72 h)	[82]
	SMMC-7721	in vitro	no activity	100.0 µgmL^{-1} (72 h)	[82]
$H_2[Ni(Enro)_2][SiW_{12}O_{40}]$	SGC-7901	in vitro	IC_{50}[b] = 144.0 µgmL^{-1} (~ 32.6 µM)*	- (72 h)	[82]
	SMMC-7721	in vitro	IC_{50}[b] = 320.0 µgmL^{-1} (~ 72.6 µM)*	- (72 h)	[82]
$H_4[SiW_{12}O_{40}]$[e]	MCF-7	in vitro	no activity	100.0 µgmL^{-1} (48 h)	[80,81]
$[Cu(PPA)_2]_2[PW_{12}O_{40}]$	PC-3	in vitro	IE[c] ~ 68.0 %[d]	50.0 µgmL^{-1} (72 h)	[28]
	HeLa	in vitro	no activity	n.d. (72 h)	[28]
	Hep-G2	in vitro	no activity	n.d. (72 h)	[28]
$[HPPA]_5[CdPW_{11}O_{39}]$	PC-3	in vitro	IE[c] ~ 88.0 %[d]	50.0 µgmL^{-1} (72 h)	[28]
	HeLa	in vitro	IE[c] ~ 56.0 %[d]	50.0 µgmL^{-1} (72 h)	[28]
	Hep-G2	in vitro	no activity	n.d. (72 h)	[28]
$[HPPA]_3[PW_{12}O_{40}]$	PC-3	in vitro	IE[c] ~ 78.0 %[d]	50.0 µgmL^{-1} (72 h)	[28]
	HeLa	in vitro	no activity	n.d. (72 h)	[28]
	Hep-G2	in vitro	no activity	n.d. (72 h)	[28]
$H_3[PW_{12}O_{40}]$[e]	PC-3	in vitro	IE[c] ~ 55.0 %[d]	50.0 µgmL^{-1} (72 h)	[28]
	HeLa	in vitro	no activity	n.d. (72 h)	[28]
	Hep-G2	in vitro	no activity	n.d. (72 h)	[28]
$\{[Cu(PPA)_2]_2[H_3PMo_{12}O_{40}]\}$	SGC-7901	in vitro	IC_{50}[b] = 831.0 µgmL^{-1} (~ 251.4 µM)*	- (48 h)	[14]
$\{[Zn(PPA)_2][H_3PMo_{12}O_{40}]\}$	SGC-7901	in vitro	IC_{50}[b] = 438.0 µgmL^{-1} (~ 153.6 µM)*	- (48 h)	[14]
$H_3[PMo_{12}O_{40}]$[e]	SGC-7901	in vitro	IC_{50}[b] = 78.0 µgmL^{-1} (~ 42.7 µM)*	- (48 h)	[14]
$[Zn(PPA)_2(H_2O)]_2[Mo_8O_{26}]$	SGC-7901	in vitro	IC_{50}[b] = 124.5 µgmL^{-1} (~ 23.7 µM)*	- (72 h)	[13]
	SMMC-7721	in vitro	IC_{50}[b] = 175.2 µgmL^{-1}	- (72 h)	[13]

			(~ 33.4 µM)*		
$[Cu(Enro)_2(H_2O)_2]H_2[\beta\text{-}Mo_8O_{26}]$	SGC-7901	in vitro	IC_{50}[b] = 138.0 µgmL^{-1} (~ 66.5 µM)*	- (72 h)	[12]
$[Cu_2(PPA)_4][\delta\text{-}Mo_8O_{26}]$	SGC-7901	in vitro	no activity	100.0 µgmL^{-1} (72 h)	[12]
$[Cu_2(Norf)_2(H_2O)_2][\beta\text{-}Mo_8O_{26}]$	SGC-7901	in vitro	IC_{50}[b] = 643.0 µgmL^{-1} (~ 323.9 µM)*	- (72 h)	[12]
$[Cu_2(Enro)_2(H_2O)_4][\beta\text{-}Mo_8O_{26}]$	SGC-7901	in vitro	IC_{50}[b] = 167.0 µgmL^{-1} (~ 81.1 µM)*	- (72 h)	[12]
$[TBA]_4[Mo_8O_{26}]$[e]	SGC-7901	in vitro	IC_{50}[b] = 100.0 - 300.0 µgmL^{-1} (~ 70.1 - 210.4 µM)*	- (72 h)	[12,13]
	SMMC-7721	in vitro	no activity	200.0 µgmL^{-1} (72 h)	[13]
PPA[f]	MCF-7	in vitro	IC_{50}[b] = 217.0 µgmL^{-1} (~ 715.4 µM)*	- (n.d.)	[80]

[a] the value in parentheses indicates at which time (after addition of POM) the antitumor effect was measured, that is, in in vivo studies it is the time at which the tumor size was measured (d = days) and in in vitro studies it is the time at which the inhibitory effect was determined (h = hours). [b] IC_{50} = the dose required to inhibit or kill 50% of the tested cells. [c] IE = inhibitory effect describing the % inhibition of cells in in vitro studies. [d] only the graph plotting the antitumor activity was provided by the authors and therefore the values were read from the graph. [e] the activity of the parent POM is provided for comparison reasons. [f] the activity of pipemidic acid is provided for comparison reasons. * the values in parentheses indicate the IC_{50} value in µM unit, note that this is a rough estimation as in most cases the water content of the structure was not provided by the authors and therefore the molecular weight of the respective POM could not be defined accurately. PPA = pipemidic acid, Enro = enrofloxacin, Norf = norfloxacin, Eno = enoxacin, TBA = tetra-*n*-butyl ammonium, n.d. = not defined.

Table S8. Anticancer activity of POM-biomolecule hybrids.

POM-drug hybrid	Cell line	Exp.	Activity	Dose (time)[a]	Ref.
$\{MnMo_6O_{18}[(OCH_2)_3CNH_2]_2\}^{3-}$	MCF-7	in vitro	IC_{50}[b] = 291.7 µM	- (24 h)	[83]
(tris-POM-tris)	MDA-MB-231	in vitro	IC_{50}[b] = 216.3 µM	- (24 h)	[83]
	MCF-10A[c]	in vitro	IC_{50}[b] = 245.1 µM	- (24 h)	[83]
$\{MnMo_6O_{18}[(OCH_2)_3CNHC_6H_9O_3]_2\}^{3-}$	MCF-7	in vitro	IC_{50}[b] = 287.9 µM	- (24 h)	[83]
(AA-POM-AA)[d]	MDA-MB-231	in vitro	IC_{50}[b] > 400.0 µM	- (24 h)	[83]
	MCF-10A[c]	in vitro	IC_{50}[b] = 311.3 µM	- (24 h)	[83]
$\{MnMo_6O_{18}[(OCH_2)_3CNHC_{24}H_{39}O_4]_2\}^{3-}$	MCF-7	in vitro	IC_{50}[b] = 55.9 µM	- (24 h)	[83]
(CA-POM-CA)	MDA-MB-231	in vitro	IC_{50}[b] = 37.9 µM	- (24 h)	[83]
	MCF-10A[c]	in vitro	IC_{50}[b] = 278.2 µM	- (24 h)	[83]
$\{MnMo_6O_{18}[(OCH_2)_3CNHC_{24}H_{33}O_4]_2\}^{3-}$	MCF-7	in vitro	IC_{50}[b] = 112.7 µM	- (24 h)	[83]
(DHCA-POM-DHCA)	MDA-MB-231	in vitro	IC_{50}[b] = 149.0 µM	- (24 h)	[83]
	MCF-10A[c]	in vitro	IC_{50}[b] > 400.0 µM	- (24 h)	[83]
$\{MnMo_6O_{18}[(OCH_2)_3CNHC_{31}H_{49}O_3]_2\}^{3-}$	MCF-7	in vitro	IC_{50}[b] = 263.1 µM	- (24 h)	[83]
(CHOL-POM-CHOL)	MDA-MB-231	in vitro	IC_{50}[b] = 257.9 µM	- (24 h)	[83]
	MCF-10A[c]	in vitro	IC_{50}[b] = 395.6 µM	- (24 h)	[83]
$\{MnMo_6O_{18}[(OCH_2)_3CNHC_{16}H_{23}O_8]_2\}^{3-}$	MCF-7	in vitro	IC_{50}[b] = 243.3 µM	- (24 h)	[83]
(GAL-POM-GAL)	MDA-MB-231	in vitro	IC_{50}[b] = 203.7 µM	- (24 h)	[83]
	MCF-10A[c]	in vitro	IC_{50}[b] = 217.5 µM	- (24 h)	[83]

[a] the value in parentheses indicates at which time (after addition of POM) the antitumor effect was measured, that is, in in vivo studies it is the time at which the tumor size was measured (d = days) and in in vitro studies it is the time at which the inhibitory effect was determined (h = hours). [b] IC_{50} = the dose required to inhibit or kill 50% of the tested cells. [c] these cells are normal noncancerous cells to validate toxicity. [d] the adipic acid (AA) in AA-POM-AA is an organic molecule (not a biomolecule) and was used to evaluate the difference between organic and bioligands.

Table S9. Anticancer activity of POM-amino acid hybrids.

POM-drug hybrid	Cell line	Exp.	Activity	Dose (time)[a]	Ref.
$(HAla)_8(H_3O)_{10}[PMo_{12}O_{40}]_6$	HeLa	in vitro	IC_{50}[b] = 16.2 µM	- (n.d.)	[84]
	PC-3m	in vitro	IC_{50}[b] = 12.3 µM	- (n.d.)	[84]
$(Horn)_2(H_3O)[PMo_{12}O_{40}]$	HeLa	in vitro	IC_{50}[b] = 17.8 µM	- (72 h)	[85]
	PC-3m	in vitro	IC_{50}[b] = 20.6 µM	- (72 h)	[85]
$(Horn)_2[SiMo_{12}O_{40}]$	HeLa	in vitro	IC_{50}[b] = 41.9 µM	- (72 h)	[85]
	PC-3m	in vitro	IC_{50}[b] = 79.9 µM	- (72 h)	[85]
$(Horn)_2[GeMo_{12}O_{40}]$	HeLa	in vitro	IC_{50}[b] = 12.5 µM	- (72 h)	[85]
	PC-3m	in vitro	IC_{50}[b] = 12.7 µM	- (72 h)	[85]
$Na_4[Mo_8O_{26}(alaO)_2]$	MCF-7	in vitro	IC_{50}[b] = 69.0 µM	- (72 h)	[86]
	Hep-G2	in vitro	IC_{50}[b] = 31.0 µM	- (72 h)	[86]
	HeLa	in vitro	IC_{50}[b] > 100.0 µM	- (72 h)	[86]
	Hep-2	in vitro	IC_{50}[b] > 100.0 µM	- (72 h)	[86]
	SW-620	in vitro	IC_{50}[b] > 100.0 µM	- (72 h)	[86]
	WI-38[y]	in vitro	IC_{50}[b] > 100.0 µM	- (72 h)	[86]
$Na_4[Mo_8O_{26}(glyglyO)_2]\cdot 15\ H_2O$[c]	MCF-7	in vitro	IC_{50}[b] = 52.0 µM	- (72 h)	[86]
	Hep-G2	in vitro	IC_{50}[b] = 23.0 µM	- (72 h)	[86]
	HeLa	in vitro	IC_{50}[b] = 69.0 µM	- (72 h)	[86]
	Hep-2	in vitro	IC_{50}[b] > 100.0 µM	- (72 h)	[86]
	SW-620	in vitro	IC_{50}[b] > 100.0 µM	- (72 h)	[86]
	WI-38[e]	in vitro	IC_{50}[b] > 100.0 µM	- (72 h)	[86]
$Na_4[Mo_8O_{26}(glyglyO)_2]\cdot 12\ H_2O$[c]	MCF-7	in vitro	IC_{50}[b] > 100.0 µM	- (72 h)	[86]
	Hep-G2	in vitro	IC_{50}[b] = 56.0 µM	- (72 h)	[86]
	HeLa	in vitro	IC_{50}[b] > 100.0 µM	- (72 h)	[86]
	Hep-2	in vitro	IC_{50}[b] > 100.0 µM	- (72 h)	[86]
	SW-620	in vitro	IC_{50}[b] > 100.0 µM	- (72 h)	[86]
	WI-38[e]	in vitro	IC_{50}[b] > 100.0 µM	- (72 h)	[86]
$[Hmorph]_4[Mo_8O_{24}(OH)_2(metO)_2]\cdot 4\ H_2O$[d]	MCF-7	in vitro	IC_{50}[b] = 54.0 µM	- (72 h)	[86]
	Hep-G2	in vitro	IC_{50}[b] = 35.0 µM	- (72 h)	[86]
	HeLa	in vitro	IC_{50}[b] > 100.0 µM	- (72 h)	[86]
	Hep-2	in vitro	IC_{50}[b] > 100.0 µM	- (72 h)	[86]
	SW-620	in vitro	IC_{50}[b] > 100.0 µM	- (72 h)	[86]
	WI-38[e]	in vitro	IC_{50}[b] > 100.0 µM	- (72 h)	[86]
$[Hmorph]_4[Mo_8O_{24}(OH)_2(metO)_2]\cdot 4\ CH_3OH$[d]	MCF-7	in vitro	IC_{50}[b] = 70.0 µM	- (72 h)	[86]
	Hep-G2	in vitro	IC_{50}[b] = 47.0 µM	- (72 h)	[86]
	HeLa	in vitro	IC_{50}[b] > 100.0 µM	- (72 h)	[86]
	Hep-2	in vitro	IC_{50}[b] > 100.0 µM	- (72 h)	[86]
	SW-620	in vitro	IC_{50}[b] > 100.0 µM	- (72 h)	[86]
	WI-38[e]	in vitro	IC_{50}[b] > 100.0 µM	- (72 h)	[86]

$[Hmorph]_4[Mo_8O_{24}(OH)_2(alaO)_2]$	MCF-7	in vitro	IC_{50}[b] = 32.0 µM	- (72 h)	[86]
	Hep-G2	in vitro	IC_{50}[b] = 27.0 µM	- (72 h)	[86]
	HeLa	in vitro	IC_{50}[b] > 100.0 µM	- (72 h)	[86]
	Hep-2	in vitro	IC_{50}[b] > 100.0 µM	- (72 h)	[86]
	SW-620	in vitro	IC_{50}[b] > 100.0 µM	- (72 h)	[86]
	WI-38[y]	in vitro	IC_{50}[b] > 100.0 µM	- (72 h)	[86]
$[Cu(H_2O)_3]_2[Mo_8O_{26}(pro)_2]$	MCF-7	in vitro	IC_{50}[b] = 30.8 µM	- (24 h)	[87]
	Hep-G2	in vitro	IC_{50}[b] = 60.2 µM	- (24 h)	[87]
$[Zn(H_2O)_3]_2[Mo_8O_{26}(pro)_2]$	MCF-7	in vitro	IC_{50}[b] = 97.2 µM	- (24 h)	[87]
	Hep-G2	in vitro	IC_{50}[b] = 84.3 µM	- (24 h)	[87]
$[Co(H_2O)_3]_2[Mo_8O_{26}(pro)_2]$	MCF-7	in vitro	IC_{50}[b] = 600.0 µM	- (24 h)	[87]
	Hep-G2	in vitro	IC_{50}[b] = 88.0 µM	- (24 h)	[87]
$[Cu_3(H_2O)_8(lys)_2][Mo_8O_{27}]$	MCF-7	in vitro	IC_{50}[b] = 80.6 µM	- (24 h)	[87]
	Hep-G2	in vitro	IC_{50}[b] = 26.3 µM	- (24 h)	[87]
$[Zn(H_2O)_6][Mo_8O_{26}(lysH)_2]$	MCF-7	in vitro	IC_{50}[b] = 155.1 µM	- (24 h)	[87]
	Hep-G2	in vitro	IC_{50}[b] = 111.5 µM	- (24 h)	[87]
$[Co(H_2O)_6][Mo_8O_{26}(lysH)_2]$	MCF-7	in vitro	IC_{50}[b] = 560.0 µM	- (24 h)	[87]
	Hep-G2	in vitro	IC_{50}[b] = 129.8 µM	- (24 h)	[87]
$Na_4[Mo_8O_{26}(pro)_2]$	MCF-7	in vitro	IC_{50}[b] = 450.0 µM	- (24 h)	[87]
	Hep-G2	in vitro	IC_{50}[b] = 1460.0 µM	- (24 h)	[87]
$Na_2[Mo_8O_{26}(lysH)_2]$	MCF-7	in vitro	IC_{50}[b] = 190.3 µM	- (24 h)	[87]
	Hep-G2	in vitro	IC_{50}[b] = 207.3 µM	- (24 h)	[87]
$K_2Na[AsMo_6O_{21}(gly)_3]$	A-549	in vitro	IC_{50}[b] = 180.4 µM	- (72 h)	[88]
	HL-60	in vitro	IC_{50}[b] = 8.6 µM	- (24 h)	[89]
	U937	in vitro	IC_{50}[b] = 14.5 µM	- (24 h)	[89]
	HUVEC[e]	in vitro	IC_{50}[b] = 889.2 µM	- (24 h)	[89]
γ-$K_2Na_2[Mo_8O_{26}(gly)_2]$	A-549	in vitro	IC_{50}[b] = 330.2 µM	- (72 h)	[88]

[a] the value in parentheses indicates at which time (after addition of POM) the antitumor effect was measured, that is, in in vivo studies it is the time at which the tumor size was measured (d = days) and in in vitro studies it is the time at which the inhibitory effect was determined (h = hours). [b] IC_{50} = the dose required to inhibit or kill 50% of the tested cells. [c] these compounds differ in the conformation of their glygly-ligands, which has an impact on the biological activity. [d] these compounds differ in their counter cation, which has an impact on their biological activity. [e] these cells are normal noncancerous cells to validate toxicity. ala = alanine, orn = ornithine, glygly = glycylglycine, gly = glycine, met = methionine, morph = morpholine, pro = proline, lys = lysine, n.d. = not defined.

Table S10. Anticancer activity of organically functionalized Strandberg-type POMs.

POM-drug hybrid	Cell line	Exp.	Activity	Dose (time)[a]	Ref.
$[Hbiz]_5[HMo_5P_2O_{23}]$	SHY5Y	in vitro	IC_{50}[b] = 43.0 µM	- (24 h)	[22]
	SKOV-3	in vitro	IC_{50}[b] > 100.0 µM	- (24 h)	[22]
	HeLa	in vitro	IC_{50}[b] > 100.0 µM	- (24 h)	[22]
	Hep-G2	in vitro	IC_{50}[b] > 100.0 µM	- (24 h)	[22]
	EVC-304[c]	in vitro	IC_{50}[b] > 100.0 µM	- (24 h)	[22]
$[Cu(C_6H_6N_2O)_2(H_2O)_2]_2H_2[P_2Mo_5O_{23}]$	Hep-G2	in vitro	IC_{50}[b] ~ 35.5 µM[d]	- (n.d.)	[23]
$= [Cu(pia)_2(H_2O)_2]_2H_2[P_2Mo_5O_{23}]$	HCT-116	in vitro	IC_{50}[b] > 100.0 µM[d]	- (n.d.)	[23]
	SMMC-7721	in vitro	IC_{50}[b] ~ 18.0µM[d]	- (n.d.)	[23]
	HL-7702[c]	in vitro	IC_{50}[b] ~ 45.0 µM[d]	- (n.d.)	[23]
$[Cu(C_6H_6N_2O)_2(H_2O)]H_2[Cu(C_6H_6N_2O)_2(P_2Mo_5O_{23})]$	Hep-G2	in vitro	IC_{50}[b] ~ 13.0 µM[d]	- (n.d.)	[23]
$= [Cu(pia)_2(H_2O)]H_2[Cu(pia)_2(P_2Mo_5O_{23})]$	HCT-116	in vitro	IC_{50}[b] ~ 2.7 µM[d]	- (n.d.)	[23]
	SMMC-7721	in vitro	IC_{50}[b] ~ 17.0 µM[d]	- (n.d.)	[23]
	HL-7702[c]	in vitro	IC_{50}[b] ~ 45.5 µM[d]	- (n.d.)	[23]
$[Cd(C_6H_6N_2O)_2(H_2O)_2]_2H_2[P_2Mo_5O_{23}]$	Hep-G2	in vitro	IC_{50}[b] ~ 3.0 µM[d]	- (n.d.)	[23]
$= [Cd(pia)_2(H_2O)_2]_2H_2[P_2Mo_5O_{23}]$	HCT-116	in vitro	IC_{50}[b] ~ 26.0 µM[d]	- (n.d.)	[23]
	SMMC-7721	in vitro	IC_{50}[b] ~ 14.5 µM[d]	- (n.d.)	[23]
	HL-7702[c]	in vitro	IC_{50}[b] ~ 17.0 µM[d]	- (n.d.)	[23]
$Na_6[P_2Mo_5O_{23}]$[e]	SHY5Y	in vitro	IC_{50}[b] = 93.8 µM	- (24 h)	[22]
	EVC-304[c]	in vitro	IC_{50}[b] = 80.7 µM	- (24 h)	[22]
$[K_3Ca(H_2O)_4(HP_2Mo_5O_{23})]$[e]	Hep-G2	in vitro	IC_{50}[b] > 100.0 µM[d]	- (n.d.)	[23]
	HCT-116	in vitro	IC_{50}[b] > 100.0 µM[d]	- (n.d.)	[23]
	SMMC-7721	in vitro	IC_{50}[b] > 100.0 µM[d]	- (n.d.)	[23]
	HL-7702[c]	in vitro	IC_{50}[b] > 100.0 µM[d]	- (n.d.)	[23]

[a] the value in parentheses indicates at which time (after addition of POM) the antitumor effect was measured, that is, in in vivo studies it is the time at which the tumor size was measured (d = days) and in in vitro studies it is the time at which the inhibitory effect was determined (h = hours). [b] IC_{50} = the dose required to inhibit or kill 50% of the tested cells. [c] these cells are normal noncancerous cells to validate toxicity. [d] only a bar graph depicting the IC_{50} values was provided and therefore the values were read from the graph. [e] the activity of the parent structure is provided for comparison reasons. biz = benzimidazole. n.d. = not defined.

Table S11. Anticancer activity of organoimido and benzoyldiazenido functionalized hexamolybdates,

POM-drug hybrid	Cell line	Exp.	Activity	Dose (time)[a]	Ref.
$(TBA)_2[Mo_6O_{18}(\equiv NC_6H_4\text{-}3\text{-}NO_2)]$	K-562	in vitro	IE[b] = 48.4 %	100.0 µgmL^{-1} (24 h)	[90]
$(TBA)_2[Mo_6O_{18}(\equiv NC_6H_3\text{-}2\text{-}CH_3\text{-}4\text{-}NO_2)]$	K-562	in vitro	IE[b] = 49.3 %	100.0 µgmL^{-1} (24 h)	[90]
$(TBA)_2[Mo_6O_{18}(\equiv NC_6H_3\text{-}2\text{-}CH_3\text{-}5\text{-}NO_2)]$	K-562	in vitro	IE[b] = 44.4 %	100.0 µgmL^{-1} (24 h)	[90]
$(TBA)_2[Mo_6O_{18}(\equiv NC_6H_4\text{-}4\text{-}C_2H_5)]$	K-562	in vitro	IE[b] = 53.4 %	100.0 µgmL^{-1} (24 h)	[91]
$(TBA)_2[Mo_6O_{18}(\equiv NC_{10}H_{15})]$	MCF-7	in vitro	IE[b] = 48.3 %	100.0 µgmL^{-1} (24 h)	[92]
	U-251	in vitro	IC_{50}[c] = 31.1 µM	- (24 h)	[93]
$[Mo_6O_{18}(\equiv NC_6H_4\text{-}2\text{-}CH_3\text{-}6\text{-}CON(Cy)\text{-}CO\text{-}NH\text{-}Cy)]^{2-}$ (=POM-AMB-acy)	U-251	in vitro	IC_{50}[c] = 24.8 µM	- (24 h)	[93]
$(TBA)_3[Mo_6O_{18}(=N=NCOC_6H_5)]$	K-562	in vitro	IE[b] = 44.3 %	100.0 µgmL^{-1} (24 h)	[94]
$(TBA)_3[Mo_6O_{18}(=N=NCOC_6H_4\text{-}2\text{-}Cl)]$	K-562	in vitro	IE[b] = 40.0 %	100.0 µgmL^{-1} (24 h)	[94]
$(TBA)_3[Mo_6O_{18}(=N=NCOC_6H_4\text{-}3\text{-}Cl)]$	K-562	in vitro	IE[b] = 35.7 %	100.0 µgmL^{-1} (24 h)	[94]
$(TBA)_3[Mo_6O_{18}(=N=NCOC_6H_4\text{-}4\text{-}Cl)]$	K-562	in vitro	IE[b] = 40.7 %	100.0 µgmL^{-1} (24 h)	[94]
$(TBA)_3[Mo_6O_{18}(=N=NCOC_6H_3\text{-}2,4\text{-}Cl_2)]$	K-562	in vitro	IE[b] = 36.6 %	100.0 µgmL^{-1} (24 h)	[94]
$(TBA)_3[Mo_6O_{18}(=N=NCOC_6H_4\text{-}4\text{-}Br)]$	K-562	in vitro	IE[b] = 34.1 %	100.0 µgmL^{-1} (24 h)	[94]
$(TBA)_3[Mo_6O_{18}(=N=NCOC_6H_4\text{-}3\text{-}NO_2)]$	K-562	in vitro	IE[b] = 40.6 %	100.0 µgmL^{-1} (24 h)	[94]
$(TBA_3[Mo_6O_{18}(=N=NCOC_6H_4\text{-}4\text{-}NO_2)]$	K-562	in vitro	IE[b] = 44.3 %	100.0 µgmL^{-1} (24 h)	[94]
$(TBA)_3[Mo_6O_{18}(=N=NCOC_6H_4\text{-}4\text{-}OCH_3)]$	K-562	in vitro	IE[b] = 33.4 %	100.0 µgmL^{-1} (24 h)	[94]
$(TBA)_2[Mo_6O_{19}]$[d]	K-562	in vitro	IE[b] = 15.4 - 28.7 %	100.0 µgmL^{-1} (24 h)	[92,94]
	U-251	in vitro	IC_{50}[c] = 32.4 µM	- (24 h)	[93]
	MCF-7	in vitro	IE[b] ~ 30.0 %[e]	100.0 µgmL^{-1} (24 h)	[92]

[a] the value in parentheses indicates at which time (after addition of POM) the antitumor effect was measured, that is, in in vivo studies it is the time at which the tumor size was measured (d = days) and in in vitro studies it is the time at which the inhibitory effect was determined (h = hours). [b] IE = inhibitory effect describing the % inhibition of cells in in vitro studies. [c] IC_{50} = the dose required to inhibit or kill 50% of the tested cells. [c] these cells are normal noncancerous cells to validate toxicity. [d] the activity of the parent structure is provided for comparison reasons. [e] only a bar graph depicting the IC_{50} values was provided and therefore the values were read from the graph. TBA = tetra-*n*-butyl ammonium, Me = methyl.

Table S12. Anticancer activity of organically functionalized polyoxoniobates (PONbs) and other inorganic-organic hybrid POMs.

POM-drug hybrid	Cell line	Exp.	Activity	Dose (time)[a]	Ref.
$K_3Na_2[\{Cu(H_2O)(phen)\}_2\{CuNb_{11}O_{35}H_4\}]$	K-562	in vitro	IC_{50}[b] = 0.4 µM	- (48 h)	[57]
$K_4Na[\{Cu(H_2O)(2,2'\text{-bipy})\}_2\{CuNb_{11}O_{35}H_4\}]$	K-562	in vitro	IC_{50}[b] = 0.1 µM	- (48 h)	[57]
$\{Ni(en)_3\}_5H\{V^{V}Nb_8V^{IV}_8O_{44}\}$	SGC-7901	in vitro	IC_{50}[b] = 0.7 µM	- (48 h)	[58]
	SC-1680	in vitro	IC_{50}[b] = 20.5 µM	- (48 h)	[58]
	MG-63	in vitro	IC_{50}[b] = 15.8 µM	- (48 h)	[58]
$(H_2en)Na_2[\{Zn(en)_2(Hen)\}\{Zn(en)_2(H_2O)\}_2\{PNb_8V^{IV}_8O_{44}\}]$	SGC-7901	in vitro	IC_{50}[b] = 1.2 µM	- (48 h)	[58]
	SC-1680	in vitro	IC_{50}[b] = 19.8 µM	- (48 h)	[58]
	MG-63	in vitro	IC_{50}[b] = 5.4 µM	- (48 h)	[58]
$K_7H[Nb_6O_{19}]$[c]	K-562	in vitro	IC_{50}[b] > 100.0 µM	- (48 h)	[57]
	SGC-7901	in vitro	IC_{50}[b] = 14.4 µM	- (48 h)	[58]
$(chlorin)_4[SiMo_{12}O_{40}]$	A-549	in vitro	IC_{50}[b] = 6.6 µM (photo)[d]	- (48 h)	[15]
	A-549	in vitro	IE[d] = 11.0 % (dark)[d]	20.0 µM (48 h)	[15]
	A-549	in vitro	IE[d] = 86.0 % (photo)[d]	20.0 µM (48 h)	[15]
$Na_4[SiMo_{12}O_{40}]$[c]	A-549	in vitro	IE[e] = 11.0 % (dark)[d]	20.0 µM (48 h)	[15]
	A-549	in vitro	IE[e] = 31.0 % (photo)[d]	20.0 µM (48 h)	[15]
$(NH_4)_{15}\{CoSb_6O_4(H_2O)_3[Co(hmta)SbW_8O_{31}]_3\}$[f]	A2780	in vitro	IC_{50}[b] = 0.8 µM	- (72 h)	[52]
	A2780cisR	in vitro	IC_{50}[b] = 4.4 µM	- (72 h)	[52]
	OVGAR-3	in vitro	IC_{50}[b] = 1.8 µM	- (72 h)	[52]
	SKOV-3	in vitro	IC_{50}[b] = 15.0 µM	- (72 h)	[52]
	CT-26	in vitro	IC_{50}[b] = 14.7 µM	- (72 h)	[52]
	HT-29	in vitro	IC_{50}[b] = 15.6 µM	- (72 h)	[52]
	A-549	in vitro	IC_{50}[b] = 12.7 µM	- (72 h)	[52]
	MCF-7	in vitro	IC_{50}[b] = 12.2 µM	- (72 h)	[52]
	HEK-293[g]	in vitro	IC_{50}[b] = 16.2 µM	- (72 h)	[52]
$(NH_4)_8(Hhmta)_5[\Delta\text{-}Co(H_2O)_6\{CoSb_6O_4(H_2O)_3[Co(hmta)SbW_8O_{31}]_3\}]$	A2780	in vitro	IC_{50}[b] = 0.8 µM	- (72 h)	[52]
	A2780cisR	in vitro	IC_{50}[b] = 4.5 µM	- (72 h)	[52]
	OVGAR-3	in vitro	IC_{50}[b] = 1.8 µM	- (72 h)	[52]
	SKOV-3	in vitro	IC_{50}[b] = 14.8 µM	- (72 h)	[52]
	CT-26	in vitro	IC_{50}[b] = 13.0 µM	- (72 h)	[52]
	HT-29	in vitro	IC_{50}[b] = 13.5 µM	- (72 h)	[52]
	A-549	in vitro	IC_{50}[b] = 11.0 µM	- (72 h)	[52]
	MCF-7	in vitro	IC_{50}[b] = 11.1 µM	- (72 h)	[52]
	HEK-293[g]	in vitro	IC_{50}[b] = 16.1 µM	- (72 h)	[52]
$(NH_4)_8(Hhmta)_5[\Lambda\text{-}Co(H_2O)_6\{CoSb_6O_4(H_2O)_3[Co(hmta)SbW_8O_{31}]_3\}]$	A2780	in vitro	IC_{50}[b] = 0.8 µM	- (72 h)	[52]
	A2780cisR	in vitro	IC_{50}[b] = 4.4 µM	- (72 h)	[52]
	OVGAR-3	in vitro	IC_{50}[b] = 1.8 µM	- (72 h)	[52]
	SKOV-3	in vitro	IC_{50}[b] = 15.7 µM	- (72 h)	[52]
	CT-26	in vitro	IC_{50}[b] = 10.7 µM	- (72 h)	[52]

	HT-29	in vitro	IC_{50}[b] = 14.9 µM	- (72 h)	[52]
	A-549	in vitro	IC_{50}[b] = 10.3 µM	- (72 h)	[52]
	MCF-7	in vitro	IC_{50}[b] = 10.0 µM	- (72 h)	[52]
	HEK-293[g]	in vitro	IC_{50}[b] = 16.2 µM	- (72 h)	[52]
$(NH_4)_{18}[NaSb_9W_{21}O_{86}]$[c]	A2780	in vitro	IC_{50}[b] = 4.4 µM	- (72 h)	[52]
	A2780cisR	in vitro	IC_{50}[b] = 29.0 µM	- (72 h)	[52]
	OVGAR-3	in vitro	IC_{50}[b] = 8.8 µM	- (72 h)	[52]
	SKOV-3	in vitro	IC_{50}[b] > 50.0 µM	- (72 h)	[52]
	CT-26	in vitro	IC_{50}[b] > 50.0 µM	- (72 h)	[52]
	HT-29	in vitro	IC_{50}[b] > 50.0 µM	- (72 h)	[52]
	A-549	in vitro	IC_{50}[b] > 50.0 µM	- (72 h)	[52]
	MCF-7	in vitro	IC_{50}[b] = 43.7 µM	- (72 h)	[52]
	HEK-293[g]	in vitro	IC_{50}[b] = 34.5 µM	- (72 h)	[52]
$(NH_4)_8[Fe_2(AsMo_7O_{27})_2(H_2en)_2]$	Hep-G2	in vitro	IC_{50}[b] = 220.0 µgmL^{-1} (~ 77.4 µM)*	- (n.d.)	[10]
$(NH_4)_{12}[Fe_2(AsMo_7O_{27})_2]$[c]	Hep-G2	in vitro	IC_{50}[b] = 310 µgmL^{-1} (~ 106.8 µM)*	- (n.d.)	[10]
$(PPh_4)_4[Mo_8O_{26}]$	HL-60	in vitro	NE[h] = 100.0 %	10.0 µgmL^{-1} (48 h)	[95]
	K-562	in vitro	NE[h] = 78.7 %	10.0 µgmL^{-1} (48 h)	[95]
$(H_2tmen)_3[V_{10}O_{28}]$	A-549	in vitro	IC_{50}[b] = 4.3 µM	- (48 h)	[96]
	P388	in vitro	IC_{50}[b] = 1.5 µM	- (48 h)	[96]
	L-02[g]	in vitro	IC_{50}[b] = 6.5 µM	- (48 h)	[96]
$(H_2en)_3[V_{10}O_{28}]$	A-549	in vitro	IC_{50}[b] = 20.0 µM	- (48 h)	[96]
	P388	in vitro	IC_{50}[b] = 59.0 µM	- (48 h)	[96]
	L-02[g]	in vitro	IC_{50}[b] = 7.2 µM	- (48 h)	[96]
$K_2[(CH_3N^+C_5H_4COOH)_2][V_{10}O_{28}H_2]$	MCF-7	in vitro	IE[d] = 37.0 %	200.0 µM (24 h)	[97]
	A-549	in vitro	IE[d] = 42.0 %	200.0 µM (24 h)	[97]
	MCF-10A[g]	in vitro	IE[d] = 6.0 %	200.0 µM (24 h)	[97]
$[((CH_3)_3N^+CH_2COOH)_4][V_{10}O_{28}H_2][(CH_3)_3N^+CH_2COO^-]_2$	MCF-7	in vitro	IE[d] = 60.0 %	200.0 µM (24 h)	[97]
	A-549	in vitro	IE[d] = 76.0 %	200.0 µM (24 h)	[97]
	MCF-10A[g]	in vitro	IE[d] = 4.0 %	200.0 µM (24 h)	[97]
$Na_6[V_{10}O_{28}]$[c]	MCF-7	in vitro	IC_{50}[b] = 0.3 µM	- (96 h)	[55]

[a] the value in parentheses indicates at which time (after addition of POM) the antitumor effect was measured, that is, in in vivo studies it is the time at which the tumor size was measured (d = days) and in in vitro studies it is the time at which the inhibitory effect was determined (h = hours). [b] IC_{50} = the dose required to inhibit or kill 50% of the tested cells. [c] the activity of the parent structure is provided for comparison reasons. [d] (dark) and (photo) indicate the measured cytotoxicity without and with photo irradiation. [e] IE = inhibitory effect describing the % inhibition of cells in in vitro studies. [f] this compound is a racemic solution of its Δ- and Λ-enantiomer. [g] these cells are normal noncancerous cells to validate toxicity. [h] NE = necrotic effect describing the % necrosis of cells in in vitro studies. * the values in parentheses indicate the IC_{50} value in µM unit, note that this is a rough estimation as in most cases the water content of the structure was not provided by the authors and therefore the molecular weight of the respective POM could not be defined accurately. hmta = hexamethylenetetramine, en = 1,2-diaminoethane, phen = 1,10-phenanthroline, bipy = 2,2’-bipyridine, PPh_4 = tetraphenyl-phosphonium, tmen = *N*,*N*,*N*′,*N*′-tetramethylethylenediammonium, H_2en = ethylenediammonium, n.d. = not defined.

2.3. Anticancer activity of POM-based nanocomposites

Table S13. Anticancer activity of POM-chitosan nanocomposites.

POM-drug nanocomposite	Cell line	Exp.	Activity	Dose (time)[a]	Ref.
$[Co_4(H_2O)_2(PW_9O_{34})_2]$-CMC	HeLa	in vitro	no activity	2 $mgmL^{-1}$ (48 h)	[41]
$K_{10}[Co_4(H_2O)_2(PW_9O_{34})_2]$[c]	HeLa	in vitro	IC_{50}[b] = 160.0 µM	- (48 h)	[41]
$[Eu(SiW_{11}O_{39})_2]$-CMC	HeLa	in vitro	no activity	2.0 $mgmL^{-1}$ (48 h)	[98]
$K_{13}[Eu(SiW_{11}O_{39})_2]$[c]	HeLa	in vitro	IE[d] ~ 20.0 %[e]	2.0 $mgmL^{-1}$ (48 h)	[98]
$[CoTiW_{11}O_{40}]$-TMC	HeLa	in vitro	IE[d] ~ 50.0 %	12.5 $µgmL^{-1}$ (5 h)	[33]
$K_6H_2[CoTiW_{11}O_{40}]$[c]	HeLa	in vitro	IE[d] ~ 10.0 %[e]	50.0 $µgmL^{-1}$ (24 h)	[33]
$[P_2W_{17}(NbO_2)O_{61}]$-TMC	HeLa	in vitro	IE[d] ~ 5.0 %[e]	50.0 $µgmL^{-1}$ (24 h)	[33]
$K_7[P_2W_{17}(NbO_2)O_{61}]$[c]	HeLa	in vitro	IE[d] ~ 10.0 %[e]	50.0 $µgmL^{-1}$ (24 h)	[33]
$[(\eta^5\text{-}C_5H_5Ti)CoTiW_{11}O_{39}]$-CT	A-549	in vitro	IC_{50}[b] = 4900.0 µM	- (24 h)	[67]
	C2C12[f]	in vitro	IC_{50}[b] = 5300.0 µM	- (24 h)	[67]
$K_6H[(\eta^5\text{-}C_5H_5Ti)CoTiW_{11}O_{39}]$[c]	A-549	in vitro	IC_{50}[b] = 900.0 µM	- (24 h)	[67]
	C2C12[f]	in vitro	IC_{50}[b] = 4800.0 µM	- (24 h)	[67]
$[Cs\subset Eu_6As_6W_{63}O_{218}(H_2O)_{14}(OH)_4]$-CT	KB	in vitro	IC_{50}[b] = 79.0 $µgmL^{-1}$	- (24 h)	[54]
	MCF-7	in vitro	IC_{50}[b] = 46.0 $µgmL^{-1}$	- (24 h)	[54]
	PC-3	in vitro	IC_{50}[b] = 67.0 $µgmL^{-1}$	- (24 h)	[54]
	A-549	in vitro	IC_{50}[b] = 50.0 $µgmL^{-1}$	- (24 h)	[54]
$Na_{22}Cs_3[Cs\subset Eu_6As_6W_{63}O_{218}(H_2O)_{14}(OH)_4]$[c]	KB	in vitro	IC_{50}[b] = 522.0 $µgmL^{-1}$	- (24 h)	[54]
	MCF-7	in vitro	IC_{50}[b] = 608.0 $µgmL^{-1}$	- (24 h)	[54]
	PC-3	in vitro	IC_{50}[b] = 613.0 $µgmL^{-1}$	- (24 h)	[54]
	A-549	in vitro	IC_{50}[b] = 629.0 $µgmL^{-1}$	- (24 h)	[54]
$[TeW_6O_{24}]$-CT	HeLa	in vitro	IE[d] = 84.0 %	10.0 µM (48 h)	[49]
	Vero[f]	in vitro	IE[d] = 11.0 %	10.0 µM (48 h)	[49]
$Na_6[TeW_6O_{24}]$[c]	HeLa	in vitro	IE[d] = 69.0 %	10.0 µM (48 h)	[49]
	Vero[f]	in vitro	IE[d] = 16.0 %	10.0 µM (48 h)	[49]
$[NaP_5W_{30}O_{110}]$-CT	HeLa	in vitro	IE[d] = 88.0 %	10.0 µM (48 h)	[49]
	Vero[f]	in vitro	IE[d] = 12.0 %	10.0 µM (48 h)	[49]
$(NH_4)_{14}[NaP_5W_{30}O_{110}]$[c]	HeLa	in vitro	IE[d] = 74.0 %	10.0 µM (48 h)	[49]
	Vero[f]	in vitro	IE[d] = 15.0 %	10.0 µM (48 h)	[49]
$[V_{10}O_{28}]$-CT	HeLa	in vitro	IE[d] = 81.0 %	10.0 µM (48 h)	[49]
	Vero[f]	in vitro	IE[d] = 11.0 %	10.0 µM (48 h)	[49]
$Na_6[V_{10}O_{28}]$[c]	HeLa	in vitro	IE[d] = 66.0 %	10.0 µM (48 h)	[49]
	Vero[f]	in vitro	IE[d] = 16.0 %	10.0 µM (48 h)	[49]

$[PW_{12}O_{40}]$-CT	HeLa	in vitro	IC_{50}[b] = 8.4 µM	- (24 h)	[99]
	Vero[f]	in vitro	IC_{50}[b] = 15.0 µM	- (24 h)	[99]
$Na_3[PW_{12}O_{40}]$[c]	HeLa	in vitro	IC_{50}[b] = 18.5 µM	- (24 h)	[99]
	Vero[f]	in vitro	IC_{50}[b] = 18.0 µM	- (24 h)	[99]
$[Ti_2PW_{10}O_{40}]$-CT	HeLa	in vitro	IC_{50}[b] = 5.4 µM	- (24 h)	[99]
	Vero[f]	in vitro	IC_{50}[b] = 13.0 µM	- (24 h)	[99]
$K_4H_3[Ti_2PW_{10}O_{40}]$[c]	HeLa	in vitro	IC_{50}[b] = 16.0 µM	- (24 h)	[99]
	Vero[f]	in vitro	IC_{50}[b] = 14.0 µM	- (24 h)	[99]
$[CoTiW_{11}O_{40}]$-CT	HeLa	in vitro	IC_{50}[b] = 1.1 µM	- (24 h)	[99]
	Vero[f]	in vitro	IC_{50}[b] = 9.0 µM	- (24 h)	[99]
$K_6H_2[CoTiW_{11}O_{40}]$[c]	HeLa	in vitro	IC_{50}[b] = 9.2 µM	- (24 h)	[99]
	Vero[f]	in vitro	IC_{50}[b] = 11.0 µM	- (24 h)	[99]
$Na_9[Gd(W_5O_{18})_2]$-CT[h]	**BEL-7402**	**in vivo**	**TWI[i] ~ 10.0 %[e]**	**n.d. (25 d)**	**[100]**
	BEL-7402	in vitro	IE[d] ~ 10.0 %[e, g]	100.0 µgmL^{-1} (24 h)	[100]
	HeLa	in vitro	IE[d] ~ 10.0 %[e, g]	100.0 µgmL^{-1} (24 h)	[100]
$Na_9[Gd(W_5O_{18})_2]$-CT + X-rays[h]	**BEL-7402**	**in vivo**	**TWI[i] ~ 60.0 %[e]**	**n.d. (25 d)**	**[100]**
$Na_9[Gd(W_5O_{18})_2]$-CT_{siRNA}[h]	**BEL-7402**	**in vivo**	**TWI[i] ~ 53.0 %[e]**	**n.d. (25 d)**	**[100]**
$Na_9[Gd(W_5O_{18})_2]$-CT_{siRNA}[h] + X-rays[h]	**BEL-7402**	**in vivo**	**TWI[i] ~ 90.0 %[e]**	**n.d. (25 d)**	**[100]**
$[P_2W_{15}O_{56}]$-CTCl-PMMA	MCF-7	in vitro	IE[d] = 77.0 %	35.0 mgmL^{-1} (24 h)	[20]
	HeLa	in vitro	IE[d] = 75.0 %[e]	35.0 mgmL^{-1} (24 h)	[20]
	Vero[f]	in vitro	IE[d] ~ 15.0 %[e]	35.0 mgmL^{-1} (24 h)	[20]
$[P_2W_{15}O_{56}]$-CMC-PMMA	MCF-7	in vitro	IE[d]] ~ 72.0 %[e]	35.0 mgmL^{-1} (24 h)	[101]
	HeLa	in vitro	IE[d] ~ 68.0 %[e]	35.0 mgmL^{-1} (24 h)	[101]
	Vero[f]	in vitro	IE[d] ~ 10.0 %[e]	35.0 mgmL^{-1} (24 h)	[101]
$Na_{12}[P_2W_{15}O_{56}]$[c]	MCF-7	in vitro	IE[d] = 79.0 %	35.0 mgmL^{-1} (24 h)	[20]
	HeLa	in vitro	IE[d] = 85.0 %	35.0 mgmL^{-1} (24 h)	[20]
	Vero[f]	in vitro	IE[d] ~ 37.0 %[e]	35.0 mgmL^{-1} (24 h)	[20]

[a] the value in parentheses indicates at which time (after addition of POM) the antitumor effect was measured, that is, in in vivo studies it is the time at which the tumor size was measured (d = days) and in in vitro studies it is the time at which the inhibitory effect was determined (h = hours). [b] IC_{50} = the dose required to inhibit or kill 50% of the tested cells. [c] the activity of the parent structure is provided for comparison reasons. [d] IE = inhibitory effect describing the % inhibition of cells in in vitro studies. [e] only the graph plotting the antitumor activity was provided by the authors and therefore the values were read from the graph. [f] these cells are normal noncancerous cells to validate toxicity. [g] this nanocomposite has radiosensitizing effects and exerts significant activity only after X-ray exposure (please see main text), the activity was measured under both normoxic and hypoxic conditions with similar results for both cells. [h] this nanocomposite showed only bioactivity after treatment with siRNA and X-rays (for more details please see main text). [i] TWI = % tumor weight inhibition (in comparison to the control). CT = chitosan, CMC = carboxymethyl chitosan, CTCl = chitosan hydrochloride, PMMA = poly(methacrylic acid), n.d. = not defined. **In vivo results are highlighted in green**.

Table S14. Anticancer activity of POM-starch nanocomposites.

POM-drug nanocomposite	Cell line	Exp.	Activity	Dose (time)[a]	Ref.
$[CoTiW_{11}O_{40}]$-SEP	HeLa	in vitro	IC_{50}[b] = 3.8 µgmL^{-1} (28.9 µgmL^{-1})[d]	- (72 h)	[32]
	HL-60	in vitro	IC_{50}[b] = 6.2 µgmL^{-1} (46.7 µgmL^{-1})[d]	- (72 h)	[32]
$K_6H_2[CoTiW_{11}O_{40}]$[c]	HeLa	in vitro	IC_{50}[b] = 19.8 µgmL^{-1}	- (72 h)	[32]
	HL-60	in vitro	IC_{50}[b] = 20.9 µgmL^{-1}	- (72 h)	[32]
$[Si_2Ti_6W_{18}O_{77}]$-SEP	**H22**	**in vivo**	**TWI[e] = 44.2 %**	**96.0 mgkg^{-1} (11 d) (800.0 mgkg^{-1})[d]**	**[44]**
	HeLa	in vitro	IC_{50}[b] = 3.2 µgmL^{-1} (26.3 µgmL^{-1})[d]	- (24 h)	[44]
	HL.60	in vitro	IC_{50}[b] = 8.1 µgmL^{-1} (67.4 µgmL^{-1})[d]	- (24 h)	[44]
$K_8H_6[Si_2W_{18}Ti_6O_{77}]$[c]	**H22**	**in vivo**	**TWI[e] = 40.0 %**	**200.0 mgkg^{-1} (11 d)**	**[44]**
	HeLa	in vitro	IC_{50}[b] = 46.4 µgmL^{-1}	- (n.d.)	[44]
	HL.60	in vitro	IC_{50}[b] = 60.5 µgmL^{-1}	- (n.d.)	[44]
$[Co(H_2O)SiW_{11}O_{39}]$-SEP	MCF-7	in vitro	IC_{50}[b] = 6.0 µM	- (24 h)	[35]
	HEK-293	in vitro	IC_{50}[b] = 5.2 µM	- (24 h)	[35]
$K_6[Co(H_2O)SiW_{11}O_{39}]$[c]	MCF-7	in vitro	IC_{50}[b] = 6.4 µM	- (24 h)	[35]
	HEK-293	in vitro	IC_{50}[b] = 6.2 µM	- (24 h)	[35]
$[PV_2Mo_{10}O_{40}]$-SEP	MCF-7	in vitro	IC_{50}[b] = 12.1 µM	- (24 h)	[18]
	HEK-293	in vitro	IC_{50}[b] = 10.8 µM	- (24 h)	[18]
$Na_5[PV_2Mo_{10}O_{40}]$[c]	MCF-7	in vitro	IC_{50}[b] = 20.6 µM	- (24 h)	[18]
	HEK-293	in vitro	IC_{50}[b] = 12.9 µM	- (24 h)	[18]
$[Co(H_2O)SiMo_{11}O_{39}]$-SEP	MCF-7	in vitro	IC_{50}[b] = 14.5 µM	- (24 h)	[16]
	HEK-293	in vitro	IC_{50}[b] = 12.2 µM	- (24 h)	[16]
	NHF[f]	in vitro	IE[f] = 42.8 %	100.0 µM (24 h)	[16]
$K_6[Co(H_2O)SiMo_{11}O_{39}]$[c]	MCF-7	in vitro	IC_{50}[b] = 16.2 µM	- (24 h)	[16]
	HEK-293	in vitro	IC_{50}[b] = 11.0 µM	- (24 h)	[16]
	NHF[f]	in vitro	IE[f] = 1.7 %	100.0 µM (24 h)	[16]

[a] the value in parentheses indicates at which time (after addition of POM) the antitumor effect was measured, that is, in in vivo studies it is the time at which the tumor size was measured (d = days) and in in vitro studies it is the time at which the inhibitory effect was determined (h = hours). [b] IC_{50} = the dose required to inhibit or kill 50% of the tested cells. [c] the activity of the parent structure is provided for comparison reasons. [d] the value in parentheses shows the inhibitory effect (IC_{50} or TWI) based on the complete SEP, whereas the value above is based on the real POM concentration within the nanosphere. [e] TWI = % tumor weight inhibition (in comparison to the control). [f] IE = inhibitory effect describing the % inhibition of cells in in vitro studies. SEP = starch encapsulated POM. **In vivo results are highlighted in green**.

Table S15. Anticancer activity of POM-liposome nanocomposites.

POM-drug nanocomposite	Cell line	Exp.	Activity	Dose (time)[a]	Ref.
$[Si_2Ti_6W_{18}O_{77}]$-LEP (150 mm)[c]	HeLa	in vitro	IC_{50}[b] = 4.4 µgmL^{-1} (48.6 µgmL^{-1})[d]	- (n.d.)	[45]
	HL-60	in vitro	IC_{50}[b] = 5.2 µgmL^{-1} (56.6 µgmL^{-1})[d]	- (n.d.)	[45]
$[Si_2Ti_6W_{18}O_{77}]$-LEP (60 mm)[c]	HeLa	in vitro	IC_{50}[b] = 3.2 µgmL^{-1} (35.6 µgmL^{-1})[d]	- (n.d.)	[45]
	HL-60	in vitro	IC_{50}[b] = 4.6 µgmL^{-1} (50.7 µgmL^{-1})[d]	- (n.d.)	[45]
$K_8H_6[Si_2Ti_6W_{18}O_{77}]$[e]	HeLa	in vitro	IC_{50}[b] = 26.3 µgmL^{-1}	- (n.d.)	[45]
	HL-60	in vitro	IC_{50}[b] = 23.3 µgmL^{-1}	- (n.d.)	[45]
$[Co(H_2O)SiW_{11}O_{39}]$-LEP	MCF-7	in vitro	IC_{50}[b] = 6.3 µM	- (24 h)	[35]
	HEK-293	in vitro	IC_{50}[b] = 4.5 µM	- (24 h)	[35]
$K_6[Co(H_2O)SiW_{11}O_{39})]$[e]	MCF-7	in vitro	IC_{50}[b] = 6.4 µM	- (24 h)	[35]
	HEK-293	in vitro	IC_{50}[b] = 6.2 µM	- (24 h)	[35]
$[PV_2Mo_{10}O_{40}]$-LEP	MCF-7	in vitro	IC_{50}[b] = 9.5 µM	- (24 h)	[18]
	HEK-293	in vitro	IC_{50}[b] = 10.3 µM	- (24 h)	[18]
$Na_5[PV_2Mo_{10}O_{40}]$[e]	MCF-7	in vitro	IC_{50}[b] = 20.6 µM	- (24 h)	[18]
	HEK-293	in vitro	IC_{50}[b] = 12.9 µM	- (24 h)	[18]
$[Co(H_2O)SiMo_{11}O_{39}]$-LEP	MCF-7	in vitro	IC_{50}[b] = 13.4 µM	- (24 h)	[16]
	HEK-293	in vitro	IC_{50}[b] = 8.9 µM	- (24 h)	[16]
	NHF[f]	in vitro	IE[g] = 58.4 %	100.0 µM (24 h)	[16]
$K_6[Co(H_2O)SiMo_{11}O_{39}]$[e]	MCF-7	in vitro	IC_{50}[b] = 16.2 µM	- (24 h)	[16]
	HEK-293	in vitro	IC_{50}[b] = 11.0 µM	- (24 h)	[16]
	NHF[f]	in vitro	IE[g] = 1.6 %	100.0 µM (24 h)	[16]
$[CoTiW_{11}O_{40}]$-LEP	SSMC-7221	in vitro	IC_{50}[b] = 3.5 µM (15.1 µM)[d]	- (24 h)	[102]
	HL-60	in vitro	IC_{50}[b] = 3.6 µM (15.5 µM)[d]	- (24 h)	[102]
$K_6H_2[CoTiW_{11}O_{40}]$[e]	SSMC-7221	in vitro	IC_{50}[b] = 6.6 µM	- (24 h)	[102]
	HL-60	in vitro	IC_{50}[b] = 6.9 µM	- (24 h)	[102]
$[(\eta^5-C_5H_5Ti)CoW_{11}O_{39}]$-LEP	SSMC-7721	in vitro	IC_{50}[b] = 9.1 µgmL^{-1}	- (24 h)	[103]
$K_6H[(\eta^5-C_5H_5Ti)CoW_{11}O_{39}]$[e]	SSMC-7721	in vitro	IC_{50}[b] = 9.8 µgmL^{-1}	- (24 h)	[103]
$[SiTiW_{11}O_{40}]$-LEP	**HL-60**	**in vivo**	**TWI[h] = 42.0 %**	**26.4 mgkg^{-1} (n.d.) (200 mgkg^{-1})[d]**	**[104]**

	HeLa	in vitro	IC_{50}[b] = 2.2 µgmL^{-1} (16.4 µgmL^{-1})[d]	- (24 h)	[104]
	KB	in vitro	IC_{50}[b] = 2.3 µgmL^{-1} (17.2 µgmL^{-1})[d]	- (24 h)	[104]
	HUVEC[f]	in vitro	IE[g] = 50.0 %	13.2 µgmL^{-1} (24 h)	[104]
$K_6[SiTiW_{11}O_{40}]$[e]	**HL-60**	**in vivo**	**TWI[h] = 13.0 %**	**200.0 mgkg^{-1} (n.d.)**	**[104]**
	HeLa	in vitro	IC_{50}[b] = 34.5 µgmL^{-1}	- (24 h)	[104]
	KB	in vitro	IC_{50}[b] = 47.3 µgmL^{-1}	- (24 h)	[104]
	HUVEC	in vitro	IE[g] = 70.0 %	100.0 µgmL^{-1} (24 h)	[104]
$(CH_3)_{56}N_{14}[(C_{16}H_{33})_2NCONH(CH_2)_3SiNaP_5W_{29}O_{110}]$	HT-29	in vitro	IC_{50}[b] = 2.1 µM	- (36 h)	[48]
	HUVEC[f]	in vitro	IC_{50}[b] = 30.8 µM	- (36 h)	[48]
$K_{12.5}Na_{1.5}[NaP_5W_{30}O_{110}]$[e]	HT-29	in vitro	IC_{50}[b] = 3.6 µM	- (36 h)	[48]
	HUVEC[f]	in vitro	IC_{50}[b] = 34.2 µM	- (36 h)	[48]

[a] the value in parentheses indicates at which time (after addition of POM) the antitumor effect was measured, that is, in in vivo studies it is the time at which the tumor size was measured (d = days) and in in vitro studies it is the time at which the inhibitory effect was determined (h = hours). [b] IC_{50} = the dose required to inhibit or kill 50% of the tested cells. [c] the nanocomposite was synthesized in different sizes (60 and 150 mm) showing nanoparticle size dependent activity. [d] the value in parentheses shows the inhibitory effect based on the concentration of the LEP, whereas the value above is based on the real POM concentration within the nanosphere. [e] the activity of the parent structure is provided for comparison reasons. [f] these cells are normal noncancerous cells to validate toxicity. [g] IE = inhibitory effect describing the % inhibition of cells in in vitro studies.[h] TWI = % tumor weight inhibition (in comparison to the control). LEP = liposome encapsulated POM, n.d. = not defined. **In vivo results are highlighted in green**.

Table S16. Anticancer activity of POM-AuNPs, POM-silicate and other nanocomposites.

POM-drug nanocomposite	Cell line	Exp.	Activity	Dose (time)[a]	Ref.
AuNPs$^{Tyr@PTA}$ (PTA= $H_3PW_{12}O_{40}$)	A-549	in vitro	IE[b] ~ 20.0 %[c]	10.0 µM (24 h)	[105]
AuNPs$^{Tyr@PTA\text{-}Lys}$ (PTA= $H_3PW_{12}O_{40}$)	A-549	in vitro	IE[b] ~ 40.0 %[c]	10.0 µM (24 h)	[105]
AuNPs@POM (POM = see below)	B16	in vitro	IE[b] = 28.0 %	0.2 µgmL^{-1} (24 h) (0.1 mgmL^{-1})[e]	[21]
	Vero[d]	in vitro	IE[b] = 10.0 %	0.2 µgmL^{-1} (24 h) (0.1 mgmL^{-1})[e]	[21]
$(NH_4)_{15}[Na\{(Mo^V{}_2O_4)_3(\mu_2\text{-}O)_3(\mu_2\text{-}SO_3)_3(\mu_6\text{-}SO_3)\}_2]$[f]	B16	in vitro	IE[b] = 19.0 %	23.3 µgmL^{-1} (24 h)	[21]
	Vero[d]	in vitro	IE[b] = 14.0 %	23.3 µgmL^{-1} (24 h)	[21]
$[GeV_3W_9O_{40}]$-MSN-dye loaded with DOX	U-87	in vitro	IE[b] = 70.0 %	2.0 mgmL^{-1}[h] (48 h)	[30]
	normal[g]	in vitro	IE[b] ~ 40.0 %[c]	2.0 mgmL^{-1}[h] (48 h)	[30]
$(TBA)_4H_3[GeV_3W_9O_{40}]$[f]	U-87	in vitro	IE[b] = 44.0 %	50 µgmL^{-1} (48 h)	[30]
$[AsMo_6O_{21}(O_2CCH_2NH_3)_3]$@$SiO_2$	MCF-7	in vitro	IC_{50}[i] = 40.0 µgmL^{-1}	- (24 h)	[106]
$K_2Na[AsMo_6O_{21}(O_2CCH_2NH_3)_3]$[f]	MCF-7	in vitro	IC_{50}[i] = 20000.0 µgmL^{-1} (~ 15233.1 µM)*	- (24 h)	[106]
$[Mo_7O_{24}]$-gelatin	**H22**	**in vivo**	**TWI[j] ~ 70.0 %[c]**	**100.0 mgkg^{-1} (9 d)**	**[7]**
	BGC-823	in vitro	IE[b] = 75.0 %	0.5 mgmL^{-1} (44 h)	[7]
$[NH_4]_6[Mo_7O_{24}]$[f]	**H22**	**in vivo**	**TWI[j] ~ 25.0 %[c]**	**100.0 mgkg^{-1} (9 d)**	**[7]**
	BGC-823	in vitro	IE[b] = 20.0 %	0.5 mgmL^{-1} (44 h)	[7]
Pt^{IV}-PW_{11}-DSPE-PEG_{2000}	**HT-29**	**in vivo**	**TWI[j] ~ 100.0 %[c]**	**2 mgkg^{-1} (9 d)**	**[107]**
	HT-29	in vitro	IC_{50}[i] = 7.4 µM	- (48 h)	[107]
	HUVEC[d]	in vitro	IE[b] ~ 30.0 %[c]	50.0 µM (48 h)	[107]
$[PW_{11}O_{40}(SiC_3H_6NH_2)_2Pt(NH_3)_2Cl_2]^{3-}$[f]	HT-29	in vitro	IE[b] ~ 40.0 %[c]	20.0 µM (48 h)	[107]
	HUVEC[d]	in vitro	IE[b] ~ 35.0 %[c]	50.0 µM (48 h)	[107]
$Na_7[PW_{11}O_{39}]$[f]	HT-29	in vitro	IE[b] ~ 35.0 %[c]	20.0 µM (48 h)	[107]
	HUVEC[d]	in vitro	IE[b] ~ 32.0 %[c]	50.0 µM (48 h)	[107]

[a] the value in parentheses indicates at which time (after addition of POM) the antitumor effect was measured, that is, in in vivo studies it is the time at which the tumor size was measured (d = days) and in in vitro studies it is the time at which the inhibitory effect was determined (h = hours). [b] IE = inhibitory effect describing the % inhibition of cells in in vitro studies. [c] only the graph plotting the antitumor activity was provided by the authors and therefore the values were read from the graph. [d] these cells are normal noncancerous cells to validate toxicity. [e] the value in parentheses shows the inhibitory effect based on concentration of the nanocomposite, whereas the value above is based on the real POM concentration within the nanosphere. [f] the activity of the parent structure is provided for comparison reasons. [g] the normal noncancerous cells were not defined by the authors. [h] this is the concentration of the applied DOX, not of the applied POM or nanocomposite. [i] IC_{50} = the dose required to inhibit or kill 50% of the tested cells. [j] TWI = % tumor weight inhibition (in comparison to the control). AuNPs = gold nanoparticles (functionalized with POMs), AuNPs^{Tyr} = tyrosine capped gold nano particles, DOX = doxorubicin, POM@SiO_2 = POM doped silica nanoparticles, DSPE-PEG_{2000} = 1,2-distearoyl-*sn*-glycero-3-phosphoethanolamine-N-[methoxy(polyethylene glycol)-2000], n.d. = not defined. * the values in parentheses indicate the IC_{50} value in µM unit, note that this is a rough estimation as in most cases the water content of the structure was not provided by the authors and therefore the molecular weight of the respective POM could not be defined accurately. **In vivo results are highlighted in green**.

3. Supporting figures

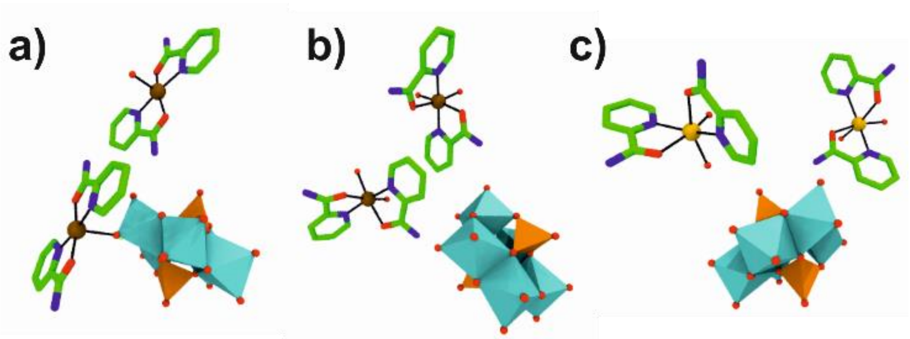

Figure S1. Structures of Strandberg-type inorganic-organic hybrids. a) $[Cu(pia)_2(H_2O)_2]_2H_2[P_2Mo_5O_{23}]$, b) $[Cu(pia)_2(H_2O)]H_2[Cu(pia)_2(P_2Mo_5O_{23})]$ and c) $[Cd(pia)_2(H_2O)_2]_2H_2[P_2Mo_5O_{23}]$. Color code: cyan polyhedra, $\{MoO_6\}$; orange polyhedra, $\{PO_4\}$; green sticks, carbon; dark blue sticks, nitrogen; brown spheres, copper; yellow spheres, cadmium; red sticks and spheres, oxygen. Black lines indicate coordination.

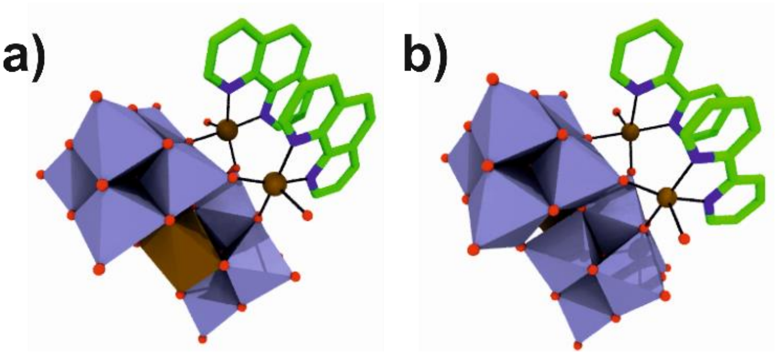

Figure S2. Structure of two PONb-based organometallo hybrids. a) $[\{Cu(H_2O)$ 1,10-phenanthroline$\}_2\{CuNb_{11}O_{35}H_4\}]^{5-}$. b) $[\{Cu(H_2O)$ 2,2′-bipyridine$\}_2\{CuNb_{11}O_{35}H_4\}]^{5-}$. Color code: lilac polyhedra, $\{NbO_6\}$; brown polyhedra and spheres, copper; green sticks, carbon; dark blue sticks, nitrogen; red spheres, oxygen. Black lines indicate coordination

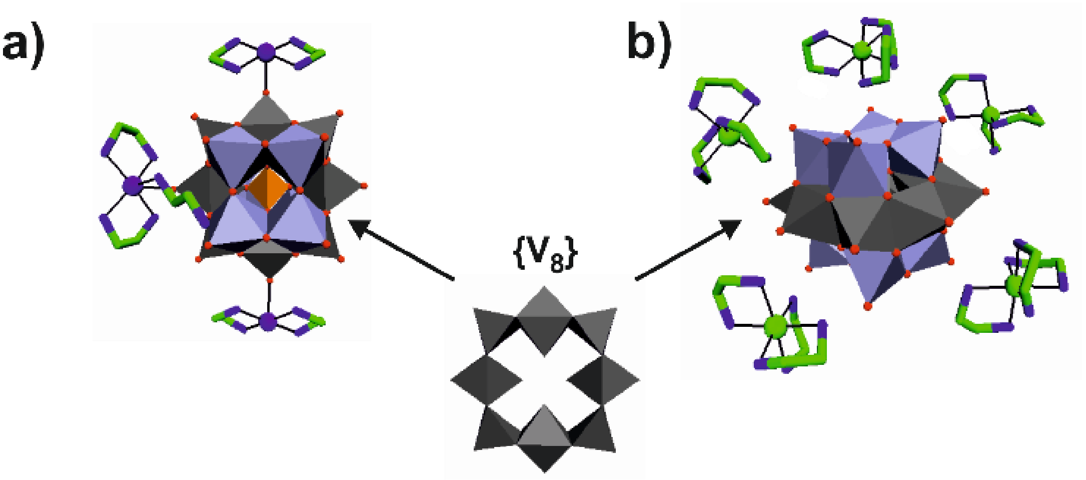

Figure S3. Structure of vanadium substituted PONb based hybrids. a) $(H_2en)Na_2[\{Zn(en)_2(Hen)\}\{Zn(en)_2(H_2O)\}_2\{PNb_8V^{IV}{}_8O_{44}\}]$. b) $\{Ni(en)_3\}_5H\{V^VNb_8V^{IV}{}_8O_{44}\}$. Both structures are obtained by the incorporation of a $\{V_8\}$ fragment into the $[XNb_8O_{36}]^{27-}$ core which is indicated by the depiction of the $\{V_8\}$ in the middle of the figure. Color code: lilac polyhedra; grey polyhedra, $\{VO_x\}$; orange polyhedra, phosphorous; green sticks, carbon; dark blue sticks, nitrogen; violet spheres, zinc; green spheres, nickel; red spheres oxygen. Black lines indicate coordination.

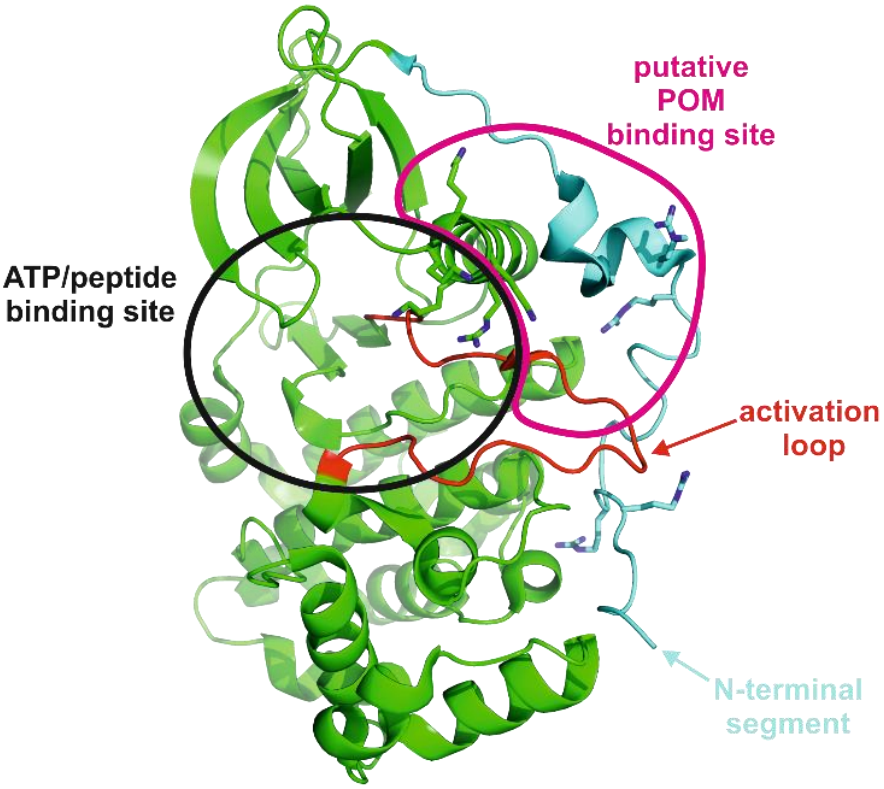

Figure S4. Representation of the putative POM binding site on protein kinase CK2. The protein structure is depicted as green cartoon and was taken from PDB entry 4GRB[108]. The ATP/peptide binding site of the kinase is marked by a black circle with the activation loop being colored in red and the N-terminal segment, which embraces the structure and the activation loop, in cyan. The suggested POM binding site is located in close proximity to the ATP/peptide binding site (labelled in pink). Moreover, potential contributors (positively charged amino acids, lysines and arginines) to POM binding are shown as sticks. Color code: green/cyan sticks, carbon; dark blue sticks, nitrogen

4. Potential of polyoxometalates as ectonucleotidase inhibitors

Table S17. Potency of POMs at ectonucleotidase inhibitors.

POM	Enzyme	K_i[a] (µM)	Ref.
$Na_6[H_2W_{12}O_{40}]$	rat NTPDase1 / 2 / 3	2.6 / 28.8 / 3.3	[109]
	human NTPDase1 / 2 / 3	0.8 / 3.3 / 0.7	[110]
	human NPP1 / 2 / 3	0.01 / > 10.0 / > 10.0	[110]
	rat eN	> 100.0	[110]
	human TSNAP	> 10.0	[110]
	calf TSAP	0.4	[26]
	porcine TNSAP	0.3	[26]
$Na_{10}[H_2W_{12}O_{40}]$	calf TSAP	0.3	[26]
	porcine TNSAP	0.5	[26]
$H_3[PW_{12}O_{40}]$	rat NTPDase1 / 2 / 3	3.5 / 6.2 / 8.7	[109]
	human NTPDase1 / 2 / 3	6.9 / 6.6 / 1.1	[110]
	human NPP1 / 2 / 3	0.04 / > 10.0 / > 10.0	[110]
	rat eN	689.0	[110]
	human TSNAP	3.5	[110]
$Na_3[PW_{12}O_{40}]$	human NTPDase1 / 2 / 3	3.7 / 3.7 / 0.8	[110]
	human NPP1 / 2 / 3	0.03 / > 10.0 / > 10.0	[110]
	rat eN	> 100.0	[110]
	human TSNAP	3.9	[110]
$K_7[Ti_2PW_{10}O_{40}]$	rat NTPDase1 / 2 / 3	2.0 / 37.4 / 4.0	[109]
	human NTPDase1 / 2 / 3	1.3 / 3.0 / 0.6	[110]
	human NPP1 / 2 / 3	0.03 / > 10.0 / > 10.0	[110]
	rat eN	> 100.0	[110]
	human TSNAP	> 10.0	[110]
$K_6H_2[TiCoW_{11}O_{40}]$	rat NTPDase1 / 2 / 3	0.1 / 0.9 / 0.6	[109]
	human NTPDase1 / 2 / 3	0.2 / 0.1 / 0.1	[110]
	human NPP1 / 2 / 3	0.001 / 12.0 / 1.4	[110]
	rat eN	14.1	[110]
	human TSNAP	5.4	[110]
$K_{10}[Co_4(H_2O)(PW_9O_{34})_2]$	rat NTPDase1 / 2 / 3	0.5 / 1.5 / 2.6	[109]
	human NTPDase1 / 2 / 3	0.004 / 0.02 / 0.1	[110]
	human NPP1 / 2 / 3	0.1 / > 10.0 / > 10.0	[110]
	rat eN	> 100.0	[110]
	human TSNAP	> 10.0	[110]
$(NH_4)_{18}[NaSb_9W_{21}O_{86}]$	rat NTPDase1 / 2 / 3	> 1000.0 / 3.9 / 3.8	[109]
	human NTPDase1 / 2 / 3	> 20.0 / 3.8 / 0.8	[110]
	human NPP1 / 2 / 3	0.005 / > 10.0 / > 10.0	[110]
	rat eN	> 100.0	[110]

	human TSNAP	> 10.0	[110]
$Na_{14}[NaP_5W_{30}O_{110}]$	human NTPDase1 / 2 / 3	0.03 / 0.02 / 0.1	[110]
	human NPP1 / 2 / 3	0.03 / > 10.0 / > 10.0	[110]
	rat eN	> 100.0	[110]
	human TSNAP	> 10.0	[110]
$Na_{20}[P_6W_{18}O_{79}]$	human NTPDase1 / 2 / 3	7.9 / 4.2 / 2.0	[110]
	human NPP1 / 2 / 3	0.1 / > 10.0 / 3.7	[110]
	rat eN	> 100.0	[110]
	human TSNAP	2.3	[110]
	calf TSAP	0.5	[26]
	porcine TNSAP	0.3	[26]
$Na_{33}[H_7P_8W_{48}O_{184}]$	human NTPDase1 / 2 / 3	0.6 / 0.2 / 0.3	[110]
	human NPP1 / 2 / 3	0.01/ 16.8 / 0.2	[110]
	rat eN	> 100.0	[110]
	human TSNAP	> 10.0	[110]
	calf TSAP	0.5	[26]
	porcine TNSAP	0.1	[26]
$Na_{16}[(O_3POPO_3)_4W_{12}O_{36}]$	human NTPDase1 / 2 / 3	8.8 / 10.4 / 3.3	[110]
	human NPP1 / 2 / 3	0.02 / > 10.0 / > 10.0	[110]
	rat eN	> 100.0	[110]
	human TSNAP	> 3.1	[110]
	calf TSAP	0.4	[26]
	porcine TNSAP	0.3	[26]
$Na_{16}[(O_3PCH_2PO_3)_4W_{12}O_{36}]$	human NTPDase1 / 2 / 3	15.6 / 9.6 / 18.5	[110]
	human NPP1 / 2 / 3	0.04 / > 10.0 / > 10.0	[110]
	rat eN	11.4	[110]
	human TSNAP	3.3	[110]
	calf TSAP	0.5	[26]
	porcine TNSAP	0.2	[26]
$Na_6[TeW_6O_{24}]$	calf TSAP	0.6	[26]
	porcine TNSAP	0.9	[26]

[a] K_i = inhibition constant; inhibitor concentration at which 50 % enzyme inhibition is observed. NTPDase = ectonucleoside triphosphate diphosphohydrolase, NPP = ectonucleotide pyrophosphatase, eN = ecto-5’-nucleotidase, TSNAP = tissue non-specific alkaline phosphatase (porcine), TSAP = tissue specific alkaline phosphatase (calf intestine).

5. References

[1] T. Yamase, H. Fujita, K. Fukushima, *Inorg. Chim. Acta* **1988**, *151*, 15–18.
[2] H. Fujita, T. Fujita, T. Sakurai, Y. Seto, *Chemotherapy* **1992**, *40*, 173–178.
[3] H. Fujita, T. Fujita, T. Sakurai, T. Yamase, Y. Seto, *Tohoku J. Exp. Med.* **1992**, *168*, 421–426.
[4] S. Mitsui, A. Ogata, H. Yanagie, H. Kasano, T. Hisa, T. Yamase, M. Eriguchi, *Biomed. Pharmacother.* **2006**, *60*, 353–358.
[5] A. Ogata, S. Mitsui, H. Yanagie, H. Kasano, T. Hisa, T. Yamase, M. Eriguchi, *Biomed. Pharmacother.* **2005**, *59*, 240–244.
[6] Y. Liu, S. Tian, S. Liu, E. Wang, *Transit. Met. Chem.* **2005**, *30*, 113–117.
[7] R. Guo, Y. Cheng, D. Ding, X. Li, L. Zhang, X. Jiang, B. Liu, *Macromol. Biosci.* **2011**, *11*, 839–847.
[8] J.-D. Compain, P. Mialane, J. Marrot, F. Sécheresse, W. Zhu, E. Oldfield, A. Dolbecq, *Chem. – Eur. J.* **2010**, *16*, 13741–13748.
[9] A. Ogata, H. Yanagie, E. Ishikawa, Y. Morishita, S. Mitsui, A. Yamashita, K. Hasumi, S. Takamoto, T. Yamase, M. Eriguchi, *Br. J. Cancer* **2008**, *98*, 399–409.
[10] Q. Wu, X. Miao, H. Wang, Y. Wu, J. Li, J. Lu, Q. Zhou, H. Ju, *Z. Naturforsch. B* **2016**, *71*, 783–788.
[11] Z. Wenxiu, W. Chuanhua, D. Shunfu, L. Yan, Z. Duo, H. Liqin, in *Proc. 2011 Int. Conf. Hum. Health Biomed. Eng.*, **2011**, pp. 594–597.
[12] J.-Q. Sha, L.-Y. Liang, P.-F. Yan, G.-M. Li, C. Wang, D.-Y. Ma, *Polyhedron* **2012**, *31*, 422–430.
[13] Q.-N. Zhang, J.-Q. Sha, H. Yan, Q.-S. Yang, Y. Zhang, L. Li, D.-W. Wang, X.-N. Yang, *Solid State Sci.* **2014**, *32*, 20–25.
[14] J. Sha, T. Zheng, E. Zhang, H. Qiu, M. Liu, H. Zhao, H. Yuan, *J. Coord. Chem.* **2013**, *66*, 977–985.
[15] I. Yoon, J. H. Kim, J. Z. Li, W. K. Lee, Y. K. Shim, *Inorg. Chem.* **2014**, *53*, 3–5.
[16] S. Dianat, A. K. Bordbar, S. Tangestaninejad, S. H. Zarkesh-Esfahani, P. Habibi, A. A. Kajani, *J. Iran. Chem. Soc.* **2016**, *13*, 1895–1904.
[17] R. Xing, F. Wang, L. Dong, A.-P. Zheng, L. Wang, W.-J. Su, T. Lin, *Food Chem.* **2016**, *197*, 205–211.
[18] S. Dianat, A.-K. Bordbar, S. Tangestaninejad, B. Yadollahi, R. Amiri, S.-H. Zarkesh-Esfahani, P. Habibi, *J. Inorg. Biochem.* **2015**, *152*, 74–81.
[19] W. Qi, B. Zhang, Y. Qi, S. Guo, R. Tian, J. Sun, M. Zhao, *Molecules* **2017**, *22*, 1535.
[20] Azizullah, Nisar-ur-Rehman, A. Haider, U. Kortz, S. Afridi, M. Sohail, S. A. Joshi, J. Iqbal, *Int. J. Pharm.* **2017**, *533*, 125–137.
[21] I. Maicas Gabas, G. Stepien, M. Moros, S. G. Mitchell, J. M. de la Fuente, *New J. Chem.* **2016**, *40*, 1039–1047.
[22] X. Qu, H. Feng, C. Ma, Y. Yang, X. Yu, *Inorg. Chem. Commun.* **2017**, *81*, 22–26.
[23] Y.-M. Ji, Y. Fang, P.-P. Han, M.-X. Li, Q.-Q. Chen, Q.-X. Han, *Inorg. Chem. Commun.* **2017**, *86*, 22–25.
[24] W. Zheng, L. Yang, Y. Liu, X. Qin, Y. Zhou, Y. Zhou, J. Liu, *Sci. Technol. Adv. Mater.* **2014**, *15*, 035010.
[25] X. Sun, Y. Wu, W. Gao, K. Enjyoji, E. Csizmadia, C. E. Müller, T. Murakami, S. C. Robson, *Gastroenterology* **2010**, *139*, 1030–1040.
[26] R. Raza, A. Matin, S. Sarwar, M. Barsukova-Stuckart, M. Ibrahim, U. Kortz, J. Iqbal, *Dalton Trans.* **2012**, *41*, 14329–14336.
[27] H. U. V. Gerth, A. Rompel, B. Krebs, J. Boos, C. Lanvers-Kaminsky, *Anticancer. Drugs* **2005**, *16*, 101–106.
[28] J.-Q. Sha, L.-Y. Liang, X. Li, Y. Zhang, H. Yan, G. Chen, *Polyhedron* **2011**, *30*, 1657–1662.
[29] L. I. Juan, L. I. Jing, Q. I. Yan-Fei, W. Hong-Fang, W. En-Bo, H. U. Chang-Wen, X. U. Lin, W. U. Xin-Yu, *Chem. J. Chin. Univ.* **2004**, *25*, 1010–1012.
[30] D. Karimian, B. Yadollahi, V. Mirkhani, *Microporous Mesoporous Mater.* **2017**, *247*, 23–30.
[31] X. Wang, J. Liu, J. Li, Y. Yang, J. Liu, B. Li, M. T. Pope, *J. Inorg. Biochem.* **2003**, *94*, 279–284.
[32] X. Wang, J. Liu, M. T. Pope, *Dalton Trans.* **2003**, *1*, 957–960.
[33] G. Geisberger, E. B. Gyenge, C. Maake, G. R. Patzke, *Carbohydr. Polym.* **2013**, *91*, 58–67.
[34] J. Li, R. Tan, R. Li, X. Wang, E. Li, F. Zhai, S. Zhang, *Inorg. Chem. Commun.* **2007**, *10*, 216–219.
[35] S. Dianat, A. K. Bordbar, S. Tangestaninejad, B. Yadollahi, S. H. Zarkesh-Esfahani, P. Habibi, *Chem. Biol. Interact.* **2014**, *215*, 25–32.
[36] H.-Y. Liu, X.-L. Pan, J.-N. Tian, H. Sun, Q. Huan, Y.-L. Huang, J.-Q. Liu, *Oncol. Lett.* **2017**, *13*, 2418–2424.
[37] Z. Zhou, D. Zhang, L. Yang, P. Ma, Y. Si, U. Kortz, J. Niu, J. Wang, *Chem. Commun.* **2013**, *49*, 5189–5191.
[38] Q. Wu, H. Ju, J. Tao, Z. Chen, J. Li, F. Wang, Q. Cai, L. Sun, X. Pan, *J. Clust. Sci.* **2015**, *26*, 1811–1820.
[39] R. Tan, X. Pang, H. Wang, S. Cui, Y. Jiang, C. Wang, X. Wang, W. Song, *Inorg. Chem. Commun.* **2012**, *25*, 70–73.
[40] X. Liu, Q. Gan, C. Feng, *J. Rare Earths* **2012**, *30*, 604–608.
[41] G. Geisberger, S. Paulus, M. Carraro, M. Bonchio, G. R. Patzke, *Chem. – Eur. J.* **2011**, *17*, 4619–4625.
[42] R. Tan, C. Wang, S. Cui, H. Wang, J. Han, R. Xie, *J. Macromol. Sci. Part A* **2014**, *51*, 33–36.
[43] I. E. León, V. Porro, S. Astrada, M. G. Egusquiza, C. I. Cabello, M. Bollati-Fogolin, S. B. Etcheverry, *Chem. Biol. Interact.* **2014**, *222*, 87–96.
[44] F. Zhai, D. Li, C. Zhang, X. Wang, R. Li, *Eur. J. Med. Chem.* **2008**, *43*, 1911–1917.
[45] X. Wang, L. Wang, J. Liu, *Chem. J. Chin. Univ.* **2003**, *24*, 17–20.
[46] L. Wang, B.-B. Zhou, K. Yu, Z.-H. Su, S. Gao, L.-L. Chu, J.-R. Liu, G.-Y. Yang, *Inorg. Chem.* **2013**, *52*, 5119–5127.
[47] L. Wang, K. Yu, J. Zhu, B. B. Zhou, J. R. Liu, G. Y. Yang, *Dalton Trans.* **2017**, *46*, 2874–2883.
[48] L. Fu, H. Gao, M. Yan, S. Li, X. Li, Z. Dai, S. Liu, *Small* **2015**, *11*, 2938–2945.
[49] H. S. Shah, R. Al-Oweini, A. Haider, U. Kortz, J. Iqbal, *Toxicol. Rep.* **2014**, *1*, 341–352.
[50] B. Zhang, J. Qiu, C. Wu, Y. Li, Z. Liu, *Int. Immunopharmacol.* **2015**, *29*, 293–301.
[51] L. Wang, K. Yu, B.-B. Zhou, Z.-H. Su, S. Gao, L.-L. Chu, J.-R. Liu, *Dalton Trans.* **2014**, *43*, 6070–6078.
[52] Z.-M. Zhang, X. Duan, S. Yao, Z. Wang, Z. Lin, Y.-G. Li, L.-S. Long, E.-B. Wang, W. Lin, *Chem. Sci.* **2016**, *7*, 4220–4229.
[53] J.-F. Liu, Y.-G. Chen, L. Meng, J. Guo, Y. Liu, M. T. Pope, *Polyhedron* **1998**, *17*, 1541–1546.
[54] D. Menon, R. T. Thomas, S. Narayanan, S. Maya, R. Jayakumar, F. Hussain, V.-K. Lakshmanan, S. V. Nair, *Carbohydr. Polym.* **2011**, *84*, 887–893.
[55] H. El Moll, W. Zhu, E. Oldfield, L. M. Rodriguez-Albelo, P. Mialane, J. Marrot, N. Vila, I. M. Mbomekallé, E. Rivière, C. Duboc, A. Dolbecq, *Inorg. Chem.* **2012**, *51*, 7921–7931.
[56] F. Zhai, X. Wang, D. Li, H. Zhang, R. Li, L. Song, *Biomed. Pharmacother.* **2009**, *63*, 51–55.

[57] J.-Y. Niu, G. Chen, J.-W. Zhao, P.-T. Ma, S.-Z. Li, J.-P. Wang, M.-X. Li, Y. Bai, B.-S. Ji, *Chem. – Eur. J.* **2010**, *16*, 7082–7086.
[58] J.-Q. Shen, Q. Wu, Y. Zhang, Z.-M. Zhang, Y.-G. Li, Y. Lu, E.-B. Wang, *Chem. – Eur. J.* **2014**, *20*, 2840–2848.
[59] X. Wang, J. Liu, R. Zhang, B. Li, J. Liu, *Main Group Met. Chem.* **2002**, *25*, 535–540.
[60] X. H. Wang, J. F. Liu, *J. Coord. Chem.* **2000**, *51*, 73–82.
[61] X. Wang, J. Liu, J. Li, J. Liu, *Inorg. Chem. Commun.* **2001**, *4*, 372–374.
[62] X.-H. Wang, H.-C. Dai, J.-F. Liu, *Transit. Met. Chem.* **1999**, *24*, 600–604.
[63] X. H. Wang, H. C. Dai, J. F. Liu, *Polyhedron* **1999**, *18*, 2293–2300.
[64] J. Liu, Y. Lu, S. Fan, *Spectrosc. Spectr. Anal.* **2012**, *32*, 2512–2514.
[65] X. Wang, J. Li, J. He, J. Liu, *Met.-Based Drugs* **2001**, *8*, 179–182.
[66] S. Dianat, A. K. Bordbar, S. Tangestaninejad, B. Yadollahi, S. H. Zarkesh-Esfahani, P. Habibi, *J. Photochem. Photobiol. B* **2013**, *124*, 27–33.
[67] V. M. Pandya, S. A. Joshi, *Asian J. Pharm. Clin. Res.* **2017**, *10*, 278–282.
[68] X.-H. Wang, J.-F. Liu, Y.-G. Chen, Q. Liu, J.-T. Liu, M. T. Pope, *Dalton Trans.* **2000**, *5*, 1139–1142.
[69] W. Xiao-Hong, H. Jiang-Hua, Z. Rong-Chang, L. Jing-Fu, *Chin. J. Chem.* **2003**, *21*, 415–418.
[70] Z. Dong, R. Tan, J. Cao, Y. Yang, C. Kong, J. Du, S. Zhu, Y. Zhang, J. Lu, B. Huang, S. Liu, *Eur. J. Med. Chem.* **2011**, *46*, 2477–2484.
[71] B. Hasenknopf, *Front. Biosci. J.* **2005**, *10*, 275–287.
[72] X. Liu, S. Wang, C. Feng, *J. Rare Earths* **2010**, *28*, 965–968.
[73] C. G. Feng, S. S. Wang, X. Liu, *Pol. J. Chem.* **2009**, *83*, 2079–2087.
[74] X. Liu, S. Wang, C. Feng, *Spectrosc. Spectr. Anal.* **2012**, *32*, 3309–3314.
[75] X. Liu, Q. Gan, C. Feng, *Inorg. Chim. Acta* **2016**, *450*, 299–303.
[76] C. Feng, Q. Gan, X. Liu, H. He, *Chin. J. Chem.* **2012**, *30*, 1589–1593.
[77] X. Liu, J. Zhao, C. G. Feng, *Acta Chim. Sin. -Chin. Ed.-* **2006**, *64*.
[78] A. Saad, W. Zhu, G. Rousseau, P. Mialane, J. Marrot, M. Haouas, F. Taulelle, R. Dessapt, H. Serier-Brault, E. Rivière, T. Kubo, E. Oldfield, A. Dolbecq, *Chem. – Eur. J.* **2015**, *21*, 10537–10547.
[79] A. Boulmier, X. Feng, O. Oms, P. Mialane, E. Rivière, C. J. Shin, J. Yao, T. Kubo, T. Furuta, E. Oldfield, A. Dolbecq, *Inorg. Chem.* **2017**, *56*, 7558–7565.
[80] C. Li, J. Lu, F. Tu, J. Chen, Y. Li, *Inorg. Chem. Commun.* **2011**, *14*, 1192–1195.
[81] J.-Q. Sha, X. Li, Y.-H. Zhou, P.-F. Yan, G.-M. Li, C. Wang, *Solid State Sci.* **2011**, *13*, 1972–1977.
[82] J. Sha, L. Sun, E. Zheng, H. Qiu, M. Liu, H. Zhao, H. Yuan, *J. Coord. Chem.* **2013**, *66*, 602–611.
[83] H.-K. Yang, Y.-X. Cheng, M.-M. Su, Y. Xiao, M.-B. Hu, W. Wang, Q. Wang, *Bioorg. Med. Chem. Lett.* **2013**, *23*, 1462–1466.
[84] Z. B. Han, E. B. Wang, G. Y. Luan, C. W. Hu, Y. P. Chang, J. Li, N. H. Hu, H. Q. Jia, *Chem. Res. Chin. Univ.* **2001**, *17*, 356–361.
[85] J. Li, Y. Qi, J. Li, H. Wang, X. Wu, L. Duan, E. Wang, *J. Coord. Chem.* **2004**, *57*, 1309–1319.
[86] M. Cindrić, T. K. Novak, S. Kraljević, M. Kralj, B. Kamenar, *Inorg. Chim. Acta* **2006**, *359*, 1673–1680.
[87] Z. Chen, H. An, H. Zhang, Y. Hu, *CrystEngComm* **2013**, *15*, 4711–4720.
[88] C. Li, W. Qi, H. Cao, Y. Qi, S. Zhang, S. Xu, J. Sun, S. Guo, *Biomed. Pharmacother.* **2016**, *79*, 78–86.
[89] C. Li, H. Cao, J. Sun, R. Tian, D. Li, Y. Qi, W. Yang, J. Li, *J. Inorg. Biochem.* **2017**, *168*, 67–75.
[90] S. Xue, A. Chai, Z. Cai, Y. Wei, C. Xiang, W. Bian, J. Shen, *Dalton Trans.* **2008**, *35*, 4770–4775.
[91] H. Yu, S. Le, X. Zeng, J. Zhang, J. Xie, *Inorg. Chem. Commun.* **2014**, *39*, 135–139.
[92] S. She, S. Bian, J. Hao, J. Zhang, J. Zhang, Y. Wei, *Chem. – Eur. J.* **2014**, *20*, 16987–16994.
[93] S. She, S. Bian, R. Huo, K. Chen, Z. Huang, J. Zhang, J. Hao, Y. Wei, *Sci. Rep.* **2016**, *6*, 33529.
[94] L. Wang, P. Yin, J. Zhang, F. Xiao, Z. Fang, W. Fu, Y. Wei, S. Xue, *Eur. J. Inorg. Chem.* **2017**, *2017*, 5475–5484.
[95] H. Thomadaki, A. Karaliota, C. Litos, A. Scorilas, *J. Med. Chem.* **2007**, *50*, 1316–1321.
[96] Y.-T. Li, C.-Y. Zhu, Z.-Y. Wu, M. Jiang, C.-W. Yan, *Transit. Met. Chem.* **2010**, *35*, 597–603.
[97] E. Kioseoglou, C. Gabriel, S. Petanidis, V. Psycharis, C. P. Raptopoulou, A. Terzis, A. Salifoglou, *Z. Anorg. Allg. Chem.* **2013**, *639*, 1407–1416.
[98] G. Geisberger, S. Paulus, E. B. Gyenge, C. Maake, G. R. Patzke, *Small* **2011**, *7*, 2808–2814.
[99] H. S. Shah, S. A. Joshi, A. Haider, U. Kortz, N. ur-Rehman, J. Iqbal, *RSC Adv.* **2015**, *5*, 93234–93242.
[100] Y. Yong, C. Zhang, Z. Gu, J. Du, Z. Guo, X. Dong, J. Xie, G. Zhang, X. Liu, Y. Zhao, *ACS Nano* **2017**, *11*, 7164–7176.
[101] Azizullah, M. Al-Rashida, A. Haider, U. Kortz, S. A. Joshi, J. Iqbal, *ChemistrySelect* **2018**, *3*, 1472–1479.
[102] Y. Yang, J. He, X. Wang, B. Li, J. Liu, *Transit. Met. Chem.* **2004**, *29*, 96–99.
[103] Y. Yang, X.-H. Wang, H. Xü, X. Chen, G.-M. Liang, J. Gou, B. Li, J.-F. Liu, X.-W. Du, *Chin. J. Chem.* **2005**, *23*, 1397–1399.
[104] X. Wang, F. Li, S. Liu, M. T. Pope, *J. Inorg. Biochem.* **2005**, *99*, 452–457.
[105] H. K. Daima, P. R. Selvakannan, R. Shukla, S. K. Bhargava, V. Bansal, *PLoS ONE* **2013**, *8*, e0079676.
[106] H. Cao, C. Li, W. Qi, X. Meng, R. Tian, Y. Qi, W. Yang, J. Li, *PLOS ONE* **2017**, *12*, e0181018.
[107] T. Sun, W. Cui, M. Yan, G. Qin, W. Guo, H. Gu, S. Liu, Q. Wu, *Adv. Mater.* **2016**, *28*, 7397–7404.
[108] J. E. Dowling, M. Alimzhanov, L. Bao, M. H. Block, C. Chuaqui, E. L. Cooke, C. R. Denz, A. Hird, S. Huang, N. A. Larsen, B. Peng, T. W. Pontz, C. Rivard-Costa, J. C. Saeh, K. Thakur, Q. Ye, T. Zhang, P. D. Lyne, *ACS Med. Chem. Lett.* **2013**, *4*, 800–805.
[109] C. E. Müller, J. Iqbal, Y. Baqi, H. Zimmermann, A. Röllich, H. Stephan, *Bioorg. Med. Chem. Lett.* **2006**, *16*, 5943–5947.
[110] S.-Y. Lee, A. Fiene, W. Li, T. Hanck, K. A. Brylev, V. E. Fedorov, J. Lecka, A. Haider, H.-J. Pietzsch, H. Zimmermann, J. Sevigny, U. Kortz, H. Stephan, C. E. Müller, *Biochem. Pharmacol.* **2015**, *93*, 171–181.
